# Direct C(sp^2^)–H alkylation of unactivated arenes enabled by photoinduced Pd catalysis

**DOI:** 10.1038/s41467-020-19038-8

**Published:** 2020-10-19

**Authors:** Daeun Kim, Geun Seok Lee, Dongwook Kim, Soon Hyeok Hong

**Affiliations:** 1 Department of Chemistry, Korea Advanced Institute of Science and Technology (KAIST), Daejeon, 34141 Republic of Korea; 2 Department of Chemistry, College of Natural Sciences, Seoul National University, Seoul, 08826 Republic of Korea; 3 Center for Catalytic Hydrocarbon Functionalizations, Institute for Basic Science (IBS), Daejeon, 34141 Republic of Korea

**Keywords:** Homogeneous catalysis, Photocatalysis, Synthetic chemistry methodology

## Abstract

Despite the fundamental importance of efficient and selective synthesis of widely useful alkylarenes, the direct catalytic C(sp^2^)–H alkylation of unactivated arenes with a readily available alkyl halide remains elusive. Here, we report the catalytic C(sp^2^)–H alkylation reactions of unactivated arenes with alkyl bromides via visible-light induced Pd catalysis. The reaction proceeds smoothly under mild conditions without any skeletal rearrangement of the alkyl groups. The direct syntheses of structurally diverse linear and branched alkylarenes, including the late-stage phenylation of biologically active molecules and an orthogonal one-pot sequential Pd-catalyzed C–C bond-forming reaction, are achieved with exclusive chemoselectivity and exceptional functional group tolerance. Comprehensive mechanistic investigations through a combination of experimental and computational methods reveal a distinguishable Pd(0)/Pd(I) redox catalytic cycle and the origin of the counter-intuitive reactivity differences among alkyl halides.

## Abstract (web summary)

Direct catalytic C(sp^2^)–H alkylation of unactivated arenes with alkyl halides remains elusive despite the progress in C-H functionalization. Here, the authors report the catalytic C(sp^2^)–H alkylation of unactivated arenes with alkyl bromides via visible-light induced Pd catalysis.

## Introduction

**Fig. 1:**
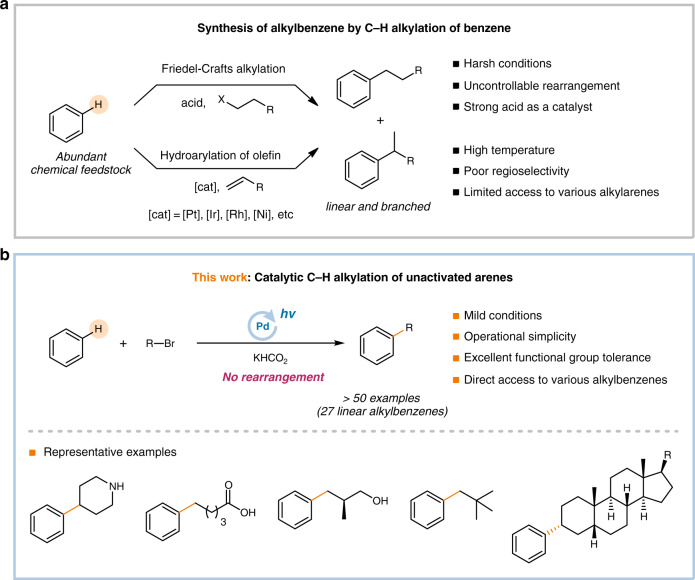
Synthesis of alkylbenzenes. **a** Synthesis of alkylbenzene by C–H alkylation of benzene. **b** Catalytic C–H alkylation of unactivated arenes.

Alkylarenes are essential scaffolds in a wide range of commodity chemicals, including surfactants and detergents, and some biologically active molecules^1,2^. The Friedel-Crafts alkylation is a textbook reaction for the synthesis of alkylarenes; however, this traditional approach has a number of drawbacks including the requirement for a strong acid catalyst, harsh reaction conditions, and the generation of undesired Wagner-Meerwein rearrangement products, which lead to complex isomeric mixtures (Fig. 1a)^3–5^. Therefore, the development of an efficient and selective synthetic method for alkylarenes is a longstanding challenge in catalysis.

In this context, the direct catalytic C–H alkylation of unactivated arenes is an ideal synthetic method to prepare alkylarenes, maximizing atom- and step-economies. For example, the transition metal-catalyzed C(sp^2^)–H alkylation of unactivated arenes with alkenes, namely the hydroarylation of alkenes, is one such approach^6,7^; however, this usually requires regioselective control and harsh reaction conditions, which limit the utility of the reaction (Fig. 1a). Recently, Hartwig and Nakao succeeded in the development of a hydroarylation reaction of unactivated arenes with alkenes in high linear/branched selectivities (>50:1). This reaction was catalyzed by a Ni complex bearing a highly sterically bulky *N*-heterocyclic carbene (NHC) ligand^8^. However, the reaction is not applicable to polar functional groups, such as nitriles and esters, due to limited functional group tolerance^9^. Although they demonstrated the high selectivity for the linear products, limited examples of the hydroarylation of unactivated arenes with a high selectivity for the branch products have been reported^10–12^.

The C(sp^2^)–H alkylation of arenes with an alkyl electrophile could be an ideal synthetic method for the construction of alkylarenes, as it would avoid the regioselectivity issues associated with the olefin hydroarylation reactions. However, despite significant advances in the field of C–H alkylation, the majority of the reported transition metal-catalyzed C(sp^2^)–H alkylation reactions using alkyl electrophiles require directing groups^13,14^, or have a limited applicability to heteroarenes^15–18^, activated electron-deficient arenes^19^, and intramolecular alkylation systems^16,20,21^. This significantly limited scope is due to the challenging oxidative addition to the alkyl electrophile and the high C–H activation barrier of the unactivated arenes (C–H bond dissociation energy, ~110 kcal/mol)^22^. Such high reaction barriers inevitably lead to a requirement for harsh reaction conditions, which are often accompanied by undesirable side reactions, such as β–hydride elimination. Therefore, a catalytic system operating under versatile and mild reaction conditions is necessary to streamline the synthesis of alkylarenes via the direct alkylation of non-activated C(sp^2^)–H bonds.

To address the challenges, we envisioned that a controlled radical-mediated reaction could afford a high activity and selectivity because the rearrangement of reactive carbon-centered radicals is significantly slower than that of their cationic counterparts^23^. Although homolytic aromatic substitution (HAS), the radical analog of the electrophilic aromatic substitution, has been well developed^24,25^, the insertion of nucleophilic alkyl radicals to the electron-rich π-system of arenes is significantly more sluggish and the alkyl radicals are prone to undergo side reactions, such as homodimerization and hydrogen atom transfer (HAT)^23,26,27^. To control the reaction pathways, the recently reported photoinduced Pd catalysis approach was identified as an attractive activation mode to generate alkyl radicals from alkyl halides, since it operates under mild conditions while controlling the concentration of free radical species to suppress undesired side reactions^16,19,28–36^. Applying the photoinduced Pd catalysis, Gevorgyan and co-workers first succeeded in developing the photoinduced Pd-catalyzed Heck reaction of alkyl halides by utilizing Pd(I)/alkyl radical hybrid species generated by single-electron transfer (SET) activation^37,38^. Further elegant achievements have been reported for various synthetically useful reactions, including Heck reaction^28,34,39–41^, desaturation^31^, hydrodehalogenation^42^, C–H alkylation of (hetero)arenes^16,19^, carbonylation^36^, and 1,4-difunctionalization of conjugated dienes^32,43,44^, exploring the Pd catalysis under visible light irradiation.

Herein we report the catalytic C(sp^2^)–H alkylation of benzene with readily available alkyl bromides without any rearrangement enabled by photoinduced Pd catalysis (Fig. 1b). Exclusive chemoselectivity and excellent functional group tolerance are demonstrated by synthesizing various linear and branched alkylbenzenes including the late-stage phenylation of bioactive molecule derivatives and an orthogonal one-pot sequential Pd-catalyzed C–C bond formation reaction. Comprehensive mechanistic investigations are conducted with a combination of experimental and computational studies to construct the complete catalytic cycle. Consequently, it clarifies the catalytic turnover mechanism involving a Pd(0)/Pd(I) redox cycle through the reciprocal exchange of a bromine atom between the Pd catalyst and the alkylating species. It also accounts for the distinctive reactivity of alkyl bromides compared to other halides by disclosing the unexpected role of the formate base which reduces the off-cycle Pd(II) dibromide complex Pd(PPh_3_)_2_Br_2_ to an active Pd(0) species.

## Results

### Reaction optimization

To examine the photoinduced Pd-catalyzed C–H alkylation of unactivated arenes with alkyl halides, benzene (**1a**) was selected as an ideal aromatic substrate (Table 1). The reaction of benzene with bromocyclohexane (**2a**) in the presence of Pd(PPh_3_)_4_ and potassium formate (KHCO_2_) gave phenylcyclohexane (**3a**) in 76% yield under irradiation with 40 W blue LED (entry 1). Among the other palladium sources investigated, Pd(PPh_3_)_2_Cl_2_ and the combination of Pd(OAc)_2_ and PPh_3_ gave comparable yields of 74% and 71%, respectively (entries 2 and 3). We found that dual phosphine ligand systems, containing both monodentate and bidentate phosphines, which were frequently utilized in previously reported photoinduced Pd catalytic processes^19,31,45^, exhibited significantly lower efficiencies (entries 4 and 5), leading to undesired β-H elimination as the major side reaction (Supplementary Table 2). The use of other bases did not improve the yield (entries 6–8). In addition, a decreased product yield was observed when the reaction was carried out at room temperature (entry 9). Therefore, both a mild base and a warm temperature (50 ± 5 °C) were found to be crucial to obtaining high conversions and yields. Among the alkyl halides tested, the alkyl bromide was found to be more reactive than the alkyl chloride and the alkyl iodide (RBr >> RCl > RI, entries 10 and 11). The addition of KBr did not exhibit any increased reactivity, but that of *n*Bu_4_NBr increased the reactivity to 17% and 36% for chlorides and iodides, respectively (Supplementary Table 3). Furthermore, halving the Pd catalyst loading led to a slight increase in yield up to 80%, presumably due to the lowered concentration of alkyl radicals that could suppress the possible side reactions (entry 12). Further lowering the amount of Pd catalyst resulted in the incomplete conversion of **2a** and a diminished yield (47%, entry 13). The use of less amount of benzene or another solvent with 10 equiv benzene gave diminished yields accompanying undesired debromination and elimination of the alkyl bromide (Supplementary Tables 4 and 5). No product formation was observed when the reaction was carried out at 100 °C without visible light irradiation (entry 14). Finally, control experiments revealed that all components were necessary to complete the alkylation reaction (entry 15).

**Table 1:** Optimization of the Pd-catalyzed C(sp^2^)–H alkylation of benzene.


Entry	Variation from standard conditions	Yield^a^ (%)
1	No deviation	76
2	Pd(PPh_3_)_2_Cl_2_ instead of Pd(PPh_3_)_4_	74
3	Pd(OAc)_2_/PPh_3_ (1:4) instead of Pd(PPh_3_)_4_	71
4	Pd(PPh_3_)_2_Cl_2_/XantPhos (1:1.2) instead of Pd(PPh_3_)_4_	52
5	Pd(PPh_3_)_4_/DPEPhos (1:1.2) instead of Pd(PPh_3_)_4_	28
6	KOAc instead of KHCO_2_	74
7	K_2_CO_3_ instead of KHCO_2_	32
8	KHMDS instead of KHCO_2_	44
9	25 °C with fan cooling	27
10	With CyCl instead of CyBr	10
11	With CyI instead of CyBr	5
12	2.5 mol% of Pd(PPh_3_)_4_	82 (80^b^)
13	1 mol% of Pd(PPh_3_)_4_	47
14	At 100 °C, in the absence of light irradiation	0
15	Without Pd(PPh_3_)_4_ or KHCO_2_ or light irradiation	0

Reaction conditions: **2a** (0.1 mmol, 0.033 M), Pd(PPh_3_)_4_ (5 mol%), KHCO_2_ (2 equiv), and benzene (3 mL) under 40 W blue LED irradiation without fan cooling (50 ± 5 °C).

^a^GC yields using dodecane as an internal standard.

^b^Isolated yield.

### Substrate scope

With the optimized conditions in hand, we investigated the scope of the reaction with respect to the alkyl bromides (Table 2). Various unfunctionalized primary alkyl bromides underwent efficient alkylation (**3b**–**3d**). Excellent compatibility was observed with a diverse set of primary alkyl bromides bearing ether, fluoro, ester, cyano, and trifluoromethyl functional groups (**3e**–**3j**). To our delight, even free alcohol (**3k**) and carboxylic acid (**3l**) functional groups were well-tolerated thanks to the mildness of the Pd photocatalytic system. Completely chemoselective functionalizations of the alkyl bromides were also observed in the presence of alkyl and aryl boronates (**3m** and **3n**) or chlorides (**3o** and **3p**), thereby opening avenues to further synthetic utilizations. The presence of functionalities neighboring the alkyl bromide was also tolerated (**3q** and **3r**), and no detrimental effect stemming from sterics was observed when β,β-disubstituted bromoalkanes (**3s** and **3t**) and even neopentyl bromide (**3u**) were employed. Noticeably, the alkyl fragment bearing an α-chiral center was retained in the obtained product (**3v**).

**Table 2:** Substrate scope of alkyl bromides.

Alkyl bromide (0.1 mmol, 0.033 M), Pd(PPh_3_)_4_ (5 mol%), KHCO_2_ (2 equiv), and benzene (3 mL) under 40 W blue LED irradiation without fan cooling (50 ± 5 °C). All yields are isolated yields.

^a^Pd(PPh_3_)_4_ (2.5 mol%).

^b^GC yields using dodecane as an internal standard.

^c^Pd(PPh_3_)_4_ (10 mol%).

^d^Isolation after methylation with TMSCH_2_N_2_.

^e^48 h.

^f^KOAc instead of KHCO_2_.

^g^4 mmol scale.

Six- and five-membered cyclic alkyl bromides reacted smoothly to form the desired alkylated products in good to excellent yields. The transformation was also effective for heterocyclic bromides such as tetrahydropyran (**3w**) and protected piperidines (**3x** and **3y**), both of which are common motifs in medicinal chemistry. The reaction was proven to be easily scalable, producing the product **3x** in 58% yield on a 4.0 mmol scale (1.1 g of **2x**). Notably, piperidine, bearing a free secondary amine moiety, could be efficiently introduced into benzene using KOAc (**3z**) as the base, consistently demonstrating the exceptional functional group tolerance of the developed reaction. *N*-formylated products were obtained (78%) when KHCO_2_ was used. A pyrrolidine scaffold could also be readily constructed on benzene (**3ab**). Norbornane (**2ac**) underwent the alkylation exclusively at the *exo* face (**3ac**)^28^. Both secondary and tertiary adamantyl groups were well-tolerated (**3ae**–**3ag**). However, the reaction was less effective with linear secondary alkyl bromides (**3ad**) and no product was observed with tertiary bromides such as *tert*-butyl bromide (Supplementary Fig. 9). The excellent functional group tolerance of this methodology was further confirmed by additive-based robustness screening experiments (Supplementary Table 7)^46^.

It should be noted that no isomeric mixture caused by an alkyl radical rearrangement was observed in any case. Indeed, the conventional Friedel-Crafts alkylation is not applicable to the synthesis of linear alkylarenes (**3b**–**3v**) due to rearrangement of the alkylating group^3–5^. Furthermore, it is noteworthy that a range of products derived from alkyl bromides are inaccessible by the hydroarylation of alkenes since suitable alkenes do not exist for the hydroarylation reaction (**3u, 3v**, and **3ae**–**3ag**), and in other cases, the hydroarylation of internal alkenes furnishes a complex mixture of regioisomers through undesired isomerization processes (**3w**–**3z**)^47,48^.

Taking advantage of the excellent functional group tolerance, operative simplicity, and mild reaction conditions of the C–H alkylation protocol, the late-stage phenylations of a wide array of biologically relevant molecules were conducted (Table 3). Specifically, the phenylations of various drug derivatives, originated from aceclofenac (**3ah**), probenecid (**3ai**), indomethacin (**3aj**), and febuxostat (**3ak**), were achieved in moderate to good yields. Reactions with alkyl bromides derived from bioactive natural products, such as hippuric acid and biotin, also proceeded smoothly to afford the desired products (**3al** and **3am**). In addition, steroid derivatives, **3an** and **3ao**, were efficiently synthesized as exclusive single diastereomers from the axial alkyl bromides of androsterone and lithocholic acid, demonstrating the potential applicability of the reaction to access diverse structures originating from native complex molecules. The stereochemistry of **3ao** was unambiguously confirmed by X-ray diffraction analysis (CCDC 1952541). These results highlight the practical utility of the reaction for the late-stage phenylation of complex molecules in the presence of biologically relevant functional groups, such as anilines, sulfonamides, electron-rich thiazoles, indoles, ureas, and steroids.

**Table 3:** Synthetic applications.

Alkyl bromide (0.1 mmol, 0.033 M), Pd(PPh_3_)_4_ (5 mol%), KHCO_2_ (2 equiv), and benzene (3 mL) under 40 W blue LED irradiation without fan cooling (50 ± 5 °C). All yields are isolated yields.

^a^Pd(PPh_3_)_4_ (10 mol%).

^b^48 h.

^c^Pd(PPh_3_)_4_ (2.5 mol%).

Inspired by the superb role of Pd catalysis in cross-coupling reactions^49^ and the exceptional chemoselectivity of the developed reactions to alkyl bromides (e.g., **3m**–**3p**), an orthogonal one-pot sequential synthesis was proposed whereby the Pd complex obtained after the photoinduced reaction could adopt the additional role of a catalyst for traditional cross-coupling reactions. To our delight, following the photoinduced phenylation of **2m**, the sequential Suzuki-Miyaura cross-coupling reaction smoothly afforded the corresponding arylated product **3mʹ** in 55% yield by simply introducing a solution of aryl bromide **5a** and K_2_CO_3_ in a mixture of EtOH/H_2_O followed by heating at 100 °C. These one-pot, sequential, Pd-catalyzed reactions using this single Pd source, driven by visible light irradiation and thermal energy, respectively, are an intriguing combination of single-electron and two-electron catalysis, which can provide an operationally simple protocol to form two C–C bonds consecutively.

The scope of the arene (C–H) coupling partners was then investigated using **2x** under the optimized conditions (Table 4). Reactions employing electronically diverse arenes efficiently afforded the desired products in a regioisomeric mixture. The observed regioselectivities are in good accordance with those previously reported for HAS reactions^25,26,45,50–52^. More specifically, reaction with anisole and halobenzenes produced compounds **4b**–**4d** in yields of 64–88%, favoring the *ortho-*product, which could be rationalized by considering the inductive effect^26^. Benzonitrile also exhibited a good reactivity, favoring *ortho*- and *para*-isomers (**4e**) due to the resonance effect of the nitrile group^26^. In addition, when 1,1,1-trifluorotoluene **1f** was employed as the coupling partner, the *para*-isomer dominated (**4f**). Hyperconjugation of the fluorine atom, which favors the formation of a positive charge in the *ortho*- and *para*-positions, could drive the unusual *para*- preferred functionalization of **1f** due to steric repulsion at the *ortho*-position^26^. A lower reactivity was observed with 1,3-benzodioxole (**4g**) due to an increased electron density. When toluene or aniline was employed as the arene substrate, poor reactivity was observed, producing a large amount of debromination byproducts, presumably due to undesired HAT reactions from benzylic C–H and N–H bonds. In terms of disubstituted arene substrates, couplings to the most electron-deficient position were dominant. For example, when 4-fluoroanisole **1h** was used, the *ortho*-coupling product relative to the fluorine atom was observed as the major isomer (**4h**), and for 1,3-difluorobenzene (**1i**), the most electronically favored 2-position was majorly alkylated (**4i**). However, to our surprise, 1,3,5-trifluorobenzene (**4k**) and 1,3,4,5-tetrafluorobenzene (**4l**) exhibited lower reactivities, while pentafluorobenzene **1m** gave no reaction, despite the fact that electrophilic polyfluoroarenes should be more reactive in HAS-type reactions, providing a further distinctive mechanistic insight of the developed reaction which will be discussed below.

**Table 4:** Substrate scope of arenes.

**2x** (0.1 mmol, 0.033 M), Pd(PPh_3_)_4_ (2.5 mol%), KHCO_2_ (2 equiv), and arene (3 mL) under 40 W blue LED irradiation without fan cooling (50 ± 5 °C). All yields are isolated yields. Regioselectivity (*ortho*/*meta*/*para*, a/b, a/b/c) is measured by GC or NMR and given in the parenthesis.

^a^Pd(PPh_3_)_4_ (5 mol%).

^b^1:1 mixture of arene and veratrole as solvent.

^c^48 h.

^d^NMR yield.

### Mechanistic investigation

**Fig. 2:**
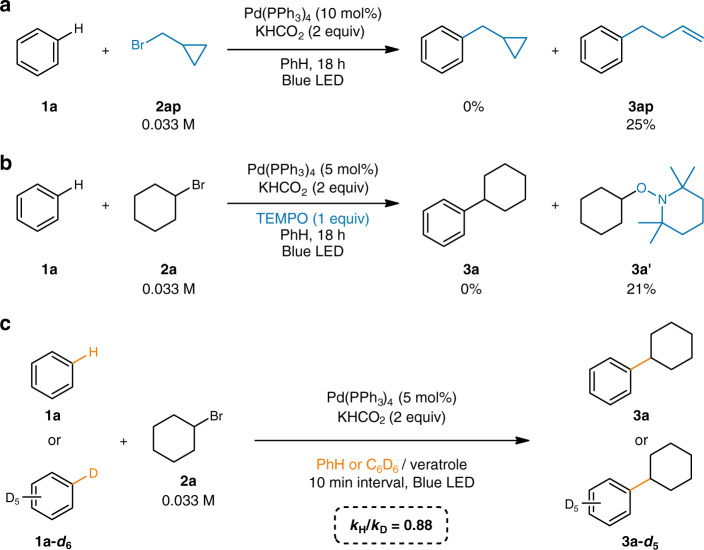
Mechanistic studies. **a** Radical clock experiment. **b** Radical scavenger experiment. **c** Kinetic isotope effect measurement.

To understand the reaction mechanism, a radical clock substrate (**2ap**) was first employed and the ring-opening product (**3ap**) was exclusively observed, indicating the involvement of a cyclopropylmethyl radical (Fig. 2a). The involvement of an alkyl radical was further confirmed by another radical scavenging experiment with TEMPO, where a cyclohexyl-TEMPO adduct (**3aʹ**) was formed (Fig. 2b). To gain additional insight in the rate-limiting step, standard kinetic isotope effect (KIE) experiments were conducted using benzene (**1a**) and benzene-*d*_6_ (**1a-*d***_**6**_) (Fig. 2c). The observed inverse secondary KIE (*k*_H_/*k*_D_ = 0.88) strongly suggests that the rate-limiting step involves a change in hybridization of the carbon atom from C(sp^2^) to C(sp^3^)^53^.

**Fig. 3:**
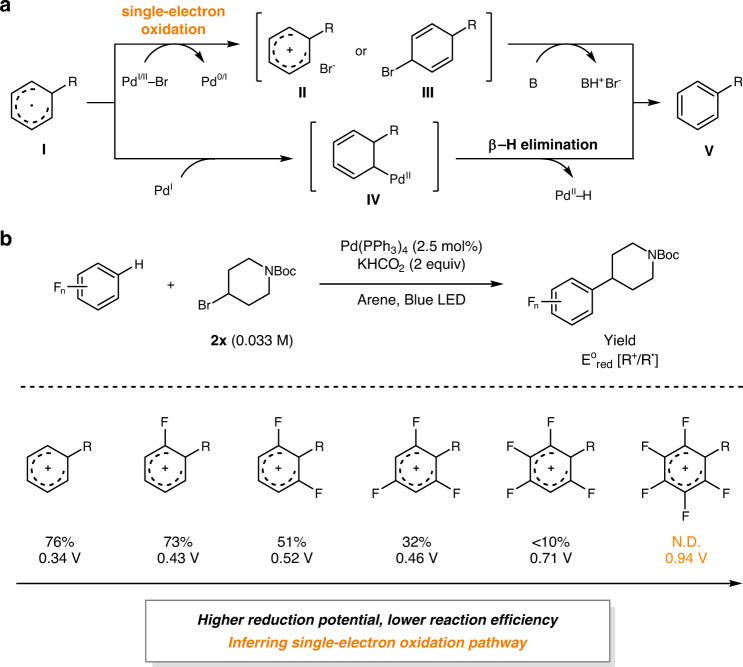
Investigation on the catalytic turnover process. **a** Two possible catalytic turnover processes. **b** Reactivity difference among polyfluoroarenes.

Following the initial mechanistic experiments, a mechanistic outline involving a Pd-mediated single-electron reduction of the alkyl bromide to generate an alkyl radical, which subsequently undergoes radical addition to the arene was constructed. To scrutinize the catalytic turnover process, i.e., regeneration of the Pd(0) species upon the formation of the desired product from the radical σ-complex (**I**), two possible pathways were proposed–(1) a single-electron oxidation of the radical σ-complex (**I**) mediated by either a Pd(I) or a Pd(II) species followed by base-assisted rearomatization (Fig. 3a, top pathway), or (2) β-hydride elimination from an Pd(II)–alkyl intermediate (**IV**) generated by the reaction between the radical σ-complex (**I**) and a Pd(I) species (Fig. 3a, bottom pathway)–based on the preceding literatures suggesting that both could be operative in photo-excited Pd catalysis^16,28,34,39,54,55^. While the formation of intermediate **IV** through spin recombination of Pd(I) species and **I** could be facile, it should be noted that the resulting Pd(II)–alkyl species could undergo triplet excitation under visible light irradiation to cleave the Pd–alkyl bond, forming a Pd(I)/alkyl radical hybrid^28,41^. This is also the case of the first step involving an alkyl bromide, as under the optimized reaction conditions, only <10% of the eliminated olefin side-products were generated albeit the generation of a Pd(I) species and the alkyl radical intermediate. Therefore, we believe that the intermediate **IV** might not undergo the β-hydride elimination effectively, allowing the reaction to go through the oxidative pathway involving **II** or **III**. To verify the photochemical behaviors of the Pd(II)–alkyl intermediates, computational analyses using time-dependent density functional theory (TD-DFT) were performed with model Pd–alkyl complexes and **IV**. The results indicated clear transitions into the Pd–C antibonding orbitals in the blue light energy region (Supplementary Table 8). Hence, although the β-hydride elimination could be thermodynamically facile, this pathway shall be kinetically inhibited by reversible formation/scission of the Pd–C bond under the visible light irradiation conditions.

Noticeably, a gradual decrease in reactivity was observed in the reactions of polyfluorinated arenes upon increasing the number of fluorine substituents (Table 4). For quantitative analysis, the calculated reduction potentials of the Wheland intermediates of the investigated polyfluorinated arenes and the related product yields are summarized in Fig. 3b. A negative correlation was observed between the reduction potential (E^o^_red_[R^+^/R·]) and the product yield, and the reactivity was completely shut down in the case of pentafluorobenzene. This illustrates that the formation of less reducing radical σ-complexes, which suppress SET to the Pd intermediate, apparently results in lowered reaction efficiencies. This observation is in clear contrast to the photoinduced Pd-catalyzed Heck reaction of alkyl bromides with styrenes recently reported by the Fu group^28^, which proposed β-hydride elimination as the product forming step, and where the electronic nature of the styrene substrate was insignificant. Possible HAT reactions from the pentafluorobenzene, which may intervene in the productive reaction pathway, were also ruled out by comparing the bond dissociation energies (BDEs) of the related C–H bonds^22,56^ and DFT computations of the HAT barrier (Supplementary Fig. 18).

With the indirect evidence of an oxidative process, we attempted to discriminate between a direct SET, to yield a cationic arenium intermediate (**II**), and a mass transfer-assisted SET^57^ to form a transient cyclohexadienyl bromide intermediate (**III**) (Fig. 3a, top pathway). To gain more information regarding the former process, we compared the DFT-calculated redox potentials of the Pd(I) bromide species and the radical σ-complexes (Supplementary Fig. 20). It revealed that the direct formation of an arenium cation intermediate (**II**) by SET without the aid of a bromine atom transfer would be highly disfavored in such nonpolar solvents, thereby indicating that a bromine atom transfer mechanism is more plausible. A similar mechanism proposed by the Zhou group, whereby single-electron oxidation occurs in a deprotonated radical anionic σ-complex (**K** to **K-anion**), was also excluded by DFT computations (Supplementary Fig. 21). While the deprotonation of the electron-poor radical σ-complexes (**K**) was feasible, deprotonation of the corresponding σ-complex derived from benzene (**E** to **E-anion**) was thermodynamically not plausible.

**Fig. 4:**
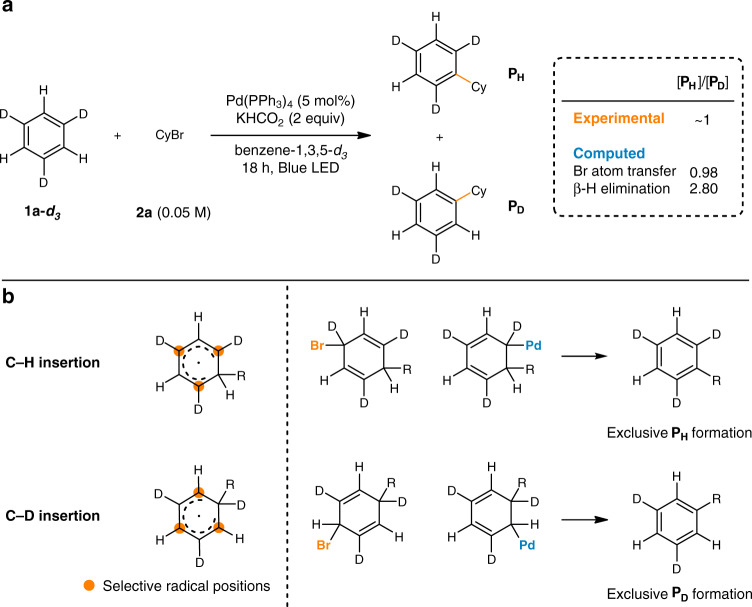
Kinetic isotope effect study with benzene-1,3,5-*d*_*3*_. **a** Intramolecular kinetic isotope effect experiment. **b** Possible intermediates and products of the reaction.

To provide a more concrete experimental evidence for the bromine atom transfer process, a kinetic experiment was designed using benzene-1,3,5-*d*_*3*_ as the arene substrate (Fig. 4). Depending on the position of alkyl radical insertion to benzene-1,3,5-*d*_*3*_, the generated radical character selectively resides on the C–H or the C–D positions and produces **P**_**H**_ and **P**_**D**_. Here, we expect significantly different KIEs as C–H bond cleavage is more involved in the β-hydride elimination pathway. The expected KIEs were first computed through DFT and was determined to be 0.98 and 2.80 for bromine atom transfer and β-hydride elimination, respectively (Supplementary Table 9). The primary KIE for β-hydride elimination is in good agreement with the C–H bond scission process. The small inverse KIE for the bromine atom transfer process could be explained because a C(sp^2^) (planar) to C(sp^3^) (tetrahedral) hybridization change is involved in the process. Moreover, the bromine atom significantly increases the reduced mass of the relevant vibrational modes, leading to a lower vibrational frequency, and hence a flat energy surface. This would result in only a small difference in the ground state zero-point energies between the protiated and deuterated substrates, thus exhibiting a negligible KIE value. An intramolecular KIE ([**P**_**H**_]/[**P**_**D**_] = ~1) was experimentally determined using benzene-1,3,5-*d*_*3*_, indicating that the bromine atom transfer is highly likely operating in the catalytic turnover process rather than the β-hydride elimination.

**Fig. 5:**
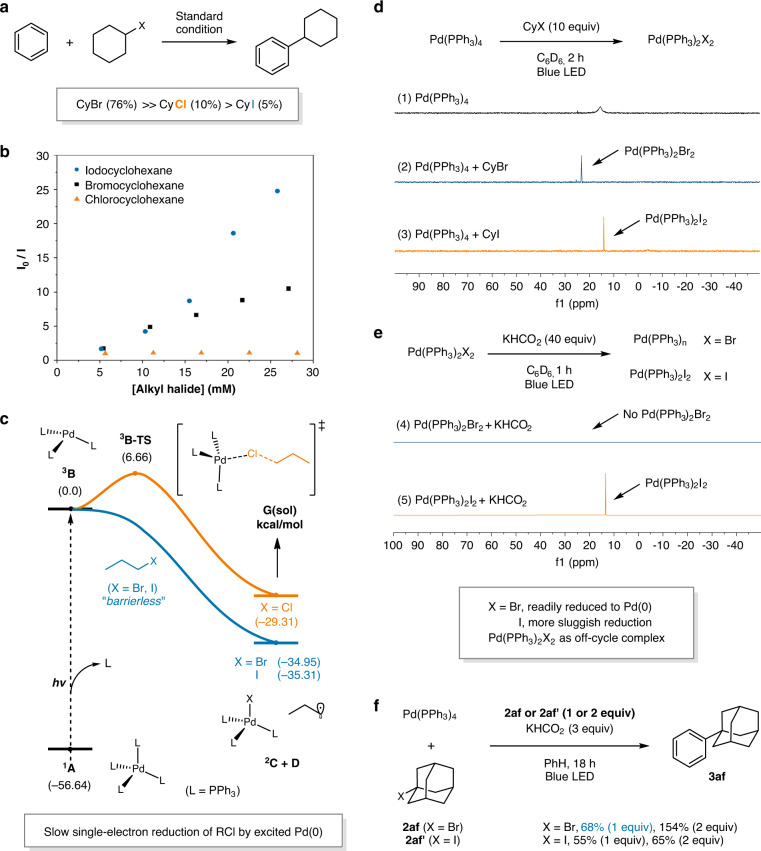
Single-electron reduction of alkyl halides by photoexcited Pd(0). **a** Reactivity difference among alkyl halides. **b** Stern-Volmer experiment. **c** DFT modeling of single-electron reduction. **d** Formation of Pd(PPh_3_)_2_X_2_ from Pd(PPh_3_)_4_ and CyX. **e** Distinguished reactivity of Pd(PPh_3_)_2_Br_2_. **f** Reactions with stoichiometric amount of Pd(PPh_3_)_4_.

Following the development of an understanding of the catalytic turnover process, we attempted to elucidate why only alkyl bromides prevailed in our reaction (Fig. 5a). In particular, the unsuitability of alkyl iodides was unexpected, since alkyl iodides are well-known common radical precursors in the reported photoexcited Pd catalysis^19,34^. The generation of alkyl radicals from alkyl chlorides was likely not as facile as from alkyl bromides and iodides, as the reduction potential of alkyl chlorides is significantly higher^58^, and this was confirmed by a Stern-Volmer quenching experiments of the photoexcited Pd(PPh_3_)_4_ with the alkyl halides under our catalytic conditions (Fig. 5b). Compared to the quenching rates of bromocyclohexane and iodocyclohexane, that of chlorocyclohexane was more sluggish with no observation of effective quenching. DFT modeling of the single-electron reduction process of the alkyl halides by a triplet excited Pd(PPh_3_)_3_ catalyst ^**3**^**B**, following the reported protocols by Cavallo and Rueping^41^, was also in good agreement with the experimental results (Fig. 5c). The reduction of alkyl chlorides was found to be associated with an energy barrier of 6.7 kcal/mol (^**3**^**B-TS**), while the reductions of alkyl bromides and iodides were barrierless. Although the reduction barrier with alkyl chlorides is not significantly high and is nearly diffusion-controlled, this reduction process is an intermolecular reaction in competition with the facile relaxation of the excited Pd(0) species ^**3**^**B**, accounting for the dramatically reduced quenching rate observed.

In contrast to the alkyl chlorides, we were puzzled as to why the alkyl iodides failed to furnish any product despite exhibiting a stronger quenching of the catalyst compared to the alkyl bromides (Fig. 5b). Since the reaction with alkyl iodides did not yield the desired product exceeding the amount of catalyst loaded (entry 11 in Table 1, and Fig. 5f), we speculated that a problem may arise in the catalytic turnover process when an alkyl iodide is subjected to the reaction. Hence, the identification of the related Pd species involved in the reaction was attempted by ^31^P NMR spectroscopy (Fig. 5d). The reaction between Pd(PPh_3_)_4_ and bromocyclohexane (10 equiv) generated Pd(PPh_3_)_2_Br_2_ (Fig. 5d), which could be readily reduced to a Pd(0) species that could re-enter the catalytic cycle with KHCO_2_ under blue light irradiation (Fig. 5e). While the formation of Pd(PPh_3_)_2_I_2_ was also observed in the reaction of Pd(PPh_3_)_4_ with iodocyclohexane (Fig. 5d), the reduction of Pd(PPh_3_)_2_I_2_ was not facile under the identical conditions (Fig. 5e), suggesting that Pd(PPh_3_)_2_I_2_ is not involved in the productive catalytic cycle. Such a Pd(II) dihalide intermediate could potentially be generated from a second SET between a photoexcited doublet Pd(I) species and an alkyl halide, as suggested by Zhao^54^ and Zhou^19^. Moreover, the DFT-computed transition states of the second single-electron reduction showed a 7.0 kcal/mol lower barrier for propyl iodide compared to propyl bromide, indicating that the inactive Pd(PPh_3_)_2_×_2_ complex can be more rapidly formed with alkyl iodides than alkyl bromides (Supplementary Fig. 22). For further confirmation, a stoichiometric experiment between Pd(PPh_3_)_4_ and **2af** or **2afʹ** was performed inspired by the studies reported by Zhao and co-workers (Fig. 5f)^54^. In their Pd-catalyzed difluoromethylation of aromatic ketones with bromodifluoroacetate (**2ar**) using Pd(PPh_3_)_4_ as the precatalyst, the desired product was not observed when the reaction was performed with a 1:1 ratio of Pd(PPh_3_)_4_ and **2ar**. The reaction only proceeded when 2 equiv of **2ar** vs Pd(PPh_3_)_4_ was used, indicating a Pd(II)-mediated SET mechanism where the generation of Pd(II) species by two consecutive single-electron reductions of the alkyl halide is critical. In clear contrast, the desired product (**3af**) was obtained in 68% yield even with 1 equiv of **2af** vs Pd(PPh_3_)_4_ under our reaction conditions (Fig. 5f). This experiment, along with the observations made by ^31^P NMR spectroscopy, therefore consistently supports that the Pd(II) species is not a catalytic intermediate, but a dormant species in our case.

**Fig. 6:**
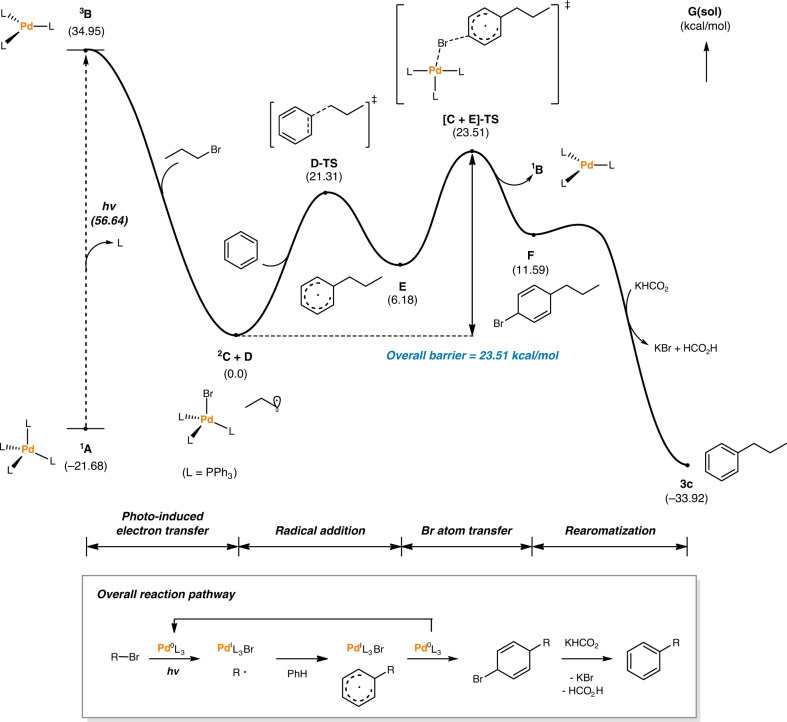
Computed energy profile of the proposed mechanism. Free energies in solution (in kcal/mol) at the B3LYP-D3(IEFPCM)/SDD/6-311++G**//B3LYP-D3/LanL2DZ/6-31G** level are displayed.

Lastly, a comprehensive DFT modeling of the overall reaction pathway was performed to verify the feasibility of the proposed mechanism using the B3LYP-D3(IEFPCM)/SDD/6-311++G**//B3LYP-D3/LanL2DZ/6-31G**^59,60^ level of theory (Fig. 6). The single-electron oxidative addition of propyl bromide to the excited Pd species ^**3**^**B**, which has 56.6 kcal/mol higher energy than the initial catalyst ^**1**^**A**, was shown to be barrierless. The generated propyl radical exists as a Pd(I)/alkyl radical hybrid species ^**2**^**C** + **D** with an energy of –35.0 kcal/mol relative to ^**3**^**B**. From the hybrid species ^**2**^**C** + **D**, an activation barrier of 21.3 kcal/mol was calculated for the transition state of the propyl radical inserting into benzene, **D-TS**. The resulting radical σ-complex **E** reacts with Pd(I)–Br species ^**2**^**C** through bromine atom transfer with a barrier of 17.3 kcal/mol to furnish the transient dearomatized cyclohexadienyl bromide **F** and the initial Pd(0) species ^**1**^**B**, which re-enters the catalytic cycle. These two elementary steps comprise the overall activation barrier of the reaction, 23.5 kcal/mol, identifying the bromine atom transfer step as the rate-limiting step, which is in good accordance with our observation that a slight warming of the reaction mixture by turning off the fan cooling was favorable to produce high conversions and yields (entry 9, Table 1). The final re-aromatization via an E2-type elimination assisted by the formate anion is highly exergonic with a downhill energy of 45.5 kcal/mol.

## Discussion

In conclusion, C(sp^2^)–H alkylation of unactivated arenes which selectively produces various linear and branched alkylarenes was developed through the use of a photoinduced Pd catalysis. The single-electron-mediated Pd catalysis controls the reactivity of the alkyl radicals generated from alkyl bromides under mild conditions, allowing the installation of various alkyl groups on arenes without the occurrence of alkyl rearrangements. This operatively simple reaction proceeds efficiently with excellent functional group tolerance, enabling the late-stage functionalization of complex molecules and a one-pot sequential Pd-catalyzed C–C bond-forming reaction. A complete Pd(0)/Pd(I) catalytic cycle was constructed with the elucidation of the origin of the counterintuitive reactivity sequence of alkyl halides through comprehensive experimental and computational studies. The developed method will streamline the synthesis of fundamentally useful alkylbenzenes.

## Methods

### General procedure for the C(sp^2^)–H alkylation of unactivated arenes

To a 4 mL vial equipped with a PTFE-coated stirrer bar were added the alkyl bromide (0.10 mmol, 1.0 equiv), Pd(PPh_3_)_4_ (5.8 mg, 0.0050 mmol, 0.050 equiv), KHCO_2_ (16.8 mg, 0.20 mmol, 2.0 equiv), and arene (3.0 mL). The resulting mixture was stirred for 18 h at ambient temperature under 40 W blue LED irradiation without fan cooling (measured reaction temperature = 50 ± 5 °C). The reaction mixture was filtered through a short pad of Celite®, eluted with CH_2_Cl_2_, and concentrated under reduced pressure. The resulting residue was purified by flash column chromatography (silica gel, hexanes/EtOAc gradient elution) to afford the desired product.

## Supplementary information

Supplementary Information

Description of Additional Supplementary Files

Supplementary Data 1

## Data Availability

The authors declare that all data supporting the findings of this study are available within the paper and its supplementary information. The supplementary information contains all experimental procedures, computational details, Cartesian coordinates of all calculated structures, characterization data (^1^H, ^13^C, ^19^F NMR, high-resolution mass spectrometry elemental analysis and crystallographic data) for all of the new compounds. The X-ray crystallographic coordinates for structure **3ao** has been deposited at the Cambridge Crystallographic Data Centre (CCDC) under the deposition number CCDC1952541 and can be obtained free of charge from The Cambridge Crystallographic Data Centre via www.ccdc.cam.ac.uk/data_request/cif.
