# Author Correction: Direct C(sp^2^)–H alkylation of unactivated arenes enabled by photoinduced Pd catalysis

**DOI:** 10.1038/s41467-020-19812-8

**Published:** 2020-11-11

**Authors:** Daeun Kim, Geun Seok Lee, Dongwook Kim, Soon Hyeok Hong

**Affiliations:** 1 Department of Chemistry, Korea Advanced Institute of Science and Technology (KAIST), Daejeon, 34141 Republic of Korea; 2 Department of Chemistry, College of Natural Sciences, Seoul National University, Seoul, 08826 Republic of Korea; 3 Center for Catalytic Hydrocarbon Functionalizations, Institute for Basic Science (IBS), Daejeon, 34141 Republic of Korea

**Keywords:** Homogeneous catalysis, Photocatalysis, Synthetic chemistry methodology

Correction to: *Nature Communications* 10.1038/s41467-020-19038-8, published online 19 October 2020.

The original version of this Article was updated shortly after publication, because an incorrect Supplementary Information file was provided. The error has now been fixed and the original Supplementary Information PDF is available to download from the HTML version of the Article and can be found as supplementary information to this correction.

