# Supplementary material for: Direct C(sp2)–H alkylation of unactivated arenes enabled by photoinduced Pd catalysis
Source: Nat Commun. 2020 Oct 19;11:5266. doi: 10.1038/s41467-020-19038-8 (PMC7572399; doi:10.1038/s41467-020-19038-8)
Supplement: Supplementary file 1 — Supplementary Information [file 41467_2020_19038_MOESM1_ESM.pdf]

Supporting Information

Palladium-Catalyzed Direct C–H Arylation of Arenes Promoted by Quaternary Ammonium Salt

Sabyuk Yang,[b] Soon Hyeok Hong*[a]

[a] Department of Chemistry, Korea Advanced Institute of Science and Technology (KAIST), Daejeon 34141, Republic of Korea

[b] Department of Chemistry, College of Natural Sciences, Seoul National University, Seoul 08826, Republic of Korea

Table of Contents

I. Experimental section

1. General information (S2)
2. Complementary reaction optimization data (S3)
3. Homo/heterogeneity studies (S7)
4. Kinetic studies (S9)
5. Gram scale reaction (S15)
6. Synthesis of compounds used in homo/heterogeneity studies (S16)
7. Characterization of products (S18)

II. References (S25)

III. NMR spectra (S26)

I. Experimental section

1. General information

Unless otherwise stated, all reactions were carried out using standard Schlenk techniques or in an argon-filled glove box. All anhydrous solvents were purchased from Aldrich and used without further purification. All 1D NMR spectroscopy experiments were conducted with a Varian 400 and 500 MHz. NMR spectra were processed with MestReNova. Chemical shifts are reported in ppm and referenced to residual solvent peaks ($CHCl_3$ in $CDCl_3$: 7.26 ppm for ^1H, 77 ppm for ^{13}C). Coupling constants are reported in Hertz. GC analyses were carried out with a 7980A GC system from Agilent Technologies, equipped with an HP-5 column and FID detector using dodecane as internal standard. FT-IR spectra were recorded on a PerkinElmer FT-IR spectrometer. Transmission electron microscopy (TEM) image was acquired on a Hitachi 7600 operating at 100 kV. Elemental analyses were conducted at the National Center for Inter-University Research Facilities of Seoul National University (NCIRF) using a Thermo Scientific Flash 2000 elemental analyzer and a Bruker Avance-600, respectively. All starting materials and reagents were purchased from Acros, Aldrich, Alfa Aesar, TCI, and Strem Chemical Inc., and used without further purification unless otherwise stated.

2. Complementary reaction optimization data

Table S1. Base screening[a]

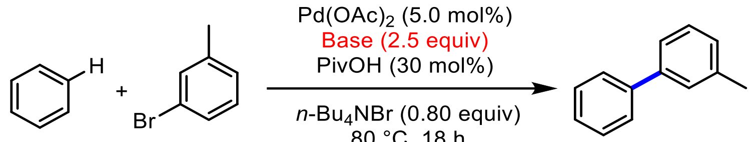

1a (1.5 mL) **2a** (0.20 mmol) **3aa**

Entry	Base	Yield (%)[b]	Entry	Base	Yield (%)[b]
1	Li_2CO_3	N. R.	6	Cs_2CO_3	61
2	Na_2CO_3	N. R.	6	K_3PO_4	44
3	K_2CO_3	25	7	CsOPiv	21
4	Rb_2CO_3	72	8	CsF	>99[c, d]

[a] Reaction conditions: **1a** (1.5 mL), **2a** (0.20 mmol), $Pd(OAc)_2$ (5.0 mol%), base (2.5 equiv), PivOH (30 mol%), *n*-Bu_4NBr (0.80 equiv), 80 °C, 18 h.
[b] Determined by gas chromatography using dodecane as an internal standard.
[c] Isolated yield.
[d] CsOPiv (0.80 equiv) used as an additional reagent.

Table S2. Ammonium salt screening[a]

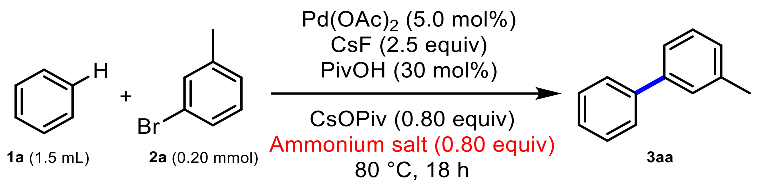

Entry	Ammonium salt	Yield (%)[b]	Entry	Ammonium salt	Yield (%)[b]
1	NH_4Br	3	6	*n*-Bu_4NOAc	66
2	Me_4NOAc	N. R.	7	*n*-Bu_4NTFA	27
3	*n*-Bu_4NCl	79	8	Me_4NBr	N. R.
4	*n*-Bu_4NBr	>99[c]	9	*n*-Hex_4NBr	77
5	*n*-Bu_4NI	3	10	*n*-Oc_4NBr	65

[a] Reaction conditions: **1a** (1.5 mL), **2a** (0.20 mmol), $Pd(OAc)_2$ (5.0 mol%), CsF (2.5 equiv), PivOH (30 mol%), CsOPiv (0.80 equiv), ammonium salt (0.80 equiv), 80 °C, 18 h.

[b] Determined by gas chromatography using dodecane as an internal standard.

[c] Isolated yield.

Table S3. Temperature screening[a]

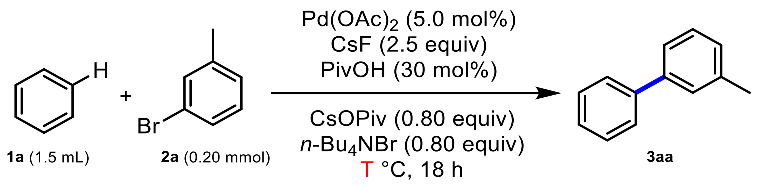

Entry	Temp (°C)	Yield (%)[b]
1	50	N. R.
2	60	36
3	65	68
4	70	90
5	80	>99[c]

[a] Reaction conditions: **1a** (1.5 mL), **2a** (0.20 mmol), $Pd(OAc)_2$ (5.0 mol%), CsF (2.5 equiv), PivOH (30 mol%), CsOPiv (0.80 equiv), *n*-Bu_4NBr (0.80 equiv), T °C, 18 h.
[b] Determined by gas chromatography using dodecane as an internal standard.
[c] Isolated yield.

Table S4. Catalyst screening[a]

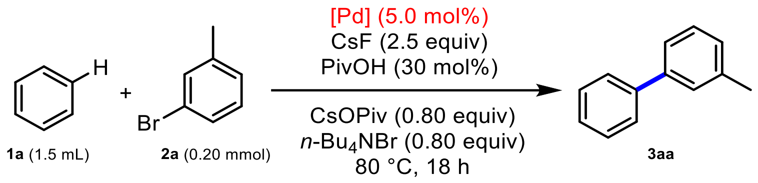

Entry	Catalyst	Yield (%)[b]	Entry	Catalyst	Yield (%)[b]
1	$PdBr_2$	91	8	$Pd(CH_3CN)_2Cl_2$	72
2	$PdCl_2$	95	9	$Pd_2(dba)_3$	33
3	$Pd(OAc)_2$	>99[c]	10	$Pd(acac)_2$	N. R.
4	$Pd(OPiv)_2$	92	11	Pd/C	N. R.
5	$PdBr_4(n\text{-}Bu_4N)_2$	92	12	$Pd(OH)_2/C$	N. R.
6	$Pd(PPh_3)_4$	N. R.	13	$Pd/CaCO_3$	N. R.
7	$PdCl_2(PPh_3)_2$	N. R.			

[a] Reaction conditions: **1a** (1.5 mL), **2a** (0.20 mmol), palladium catalyst (5.0 mol%), CsF (2.5 equiv), PivOH (30 mol%), CsOPiv (0.80 equiv), *n*-Bu_4NBr (0.80 equiv), 80 °C, 18 h.

[b] Determined by gas chromatography using dodecane as an internal standard.

[c] Isolated yield.

3. Homo/heterogeneity studies

3.1. Procedure for hot filtration

A 10 mL Schlenk tube was charged with 3-bromotoluene (0.20 mmol), cesium fluoride (2.5 equiv), cesium pivalate (0.80 equiv), palladium acetate (5.0 mol%), pivalic acid (30 mol%), tetra-*n*-butylammonium bromide (0.80 equiv), benzene (1.5 mL), and dodecane (0.20 mmol) as an internal standard inside an argon-filled glove box. The reaction vessel was placed in an oil bath and heated to 80 °C. After 3 h (condition A), a small aliquot was withdrawn and analyzed by GC, and then, the supernatant solution was filtered through a glass frit with a filter aid such as Hyflo Super Cel (Alfa Aesar), using a cannula,[1] into a new 10 mL Schlenk tube containing cesium fluoride (2.5 equiv) and cesium pivalate (0.80 equiv) (condition C). Benzene was added to the original Schlenk tube and transferred, with filtration repeated for 3–4 times. The remaining precipitate in the original Schlenk tube was charged with 3-bromotoluene (0.20 mmol), pivalic acid (30 mol%), tetra-*n*-butylammonium bromide (0.80 equiv), dodecane (0.20 mmol) containing solution in benzene (1.5 mL) (condition B). Both tubes were placed in an oil bath and stirred under 80 °C for 18 h. Then, small aliquots were withdrawn from both tubes and analyzed by GC.

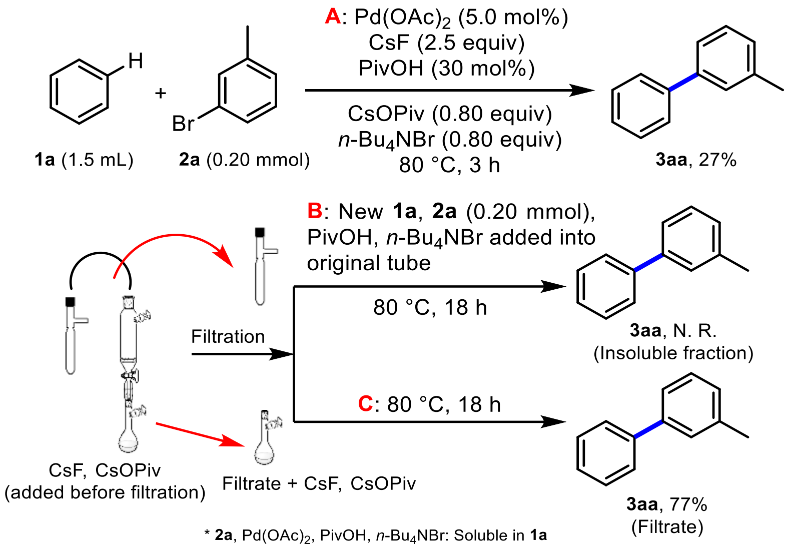

Scheme S1. Hot filtration test

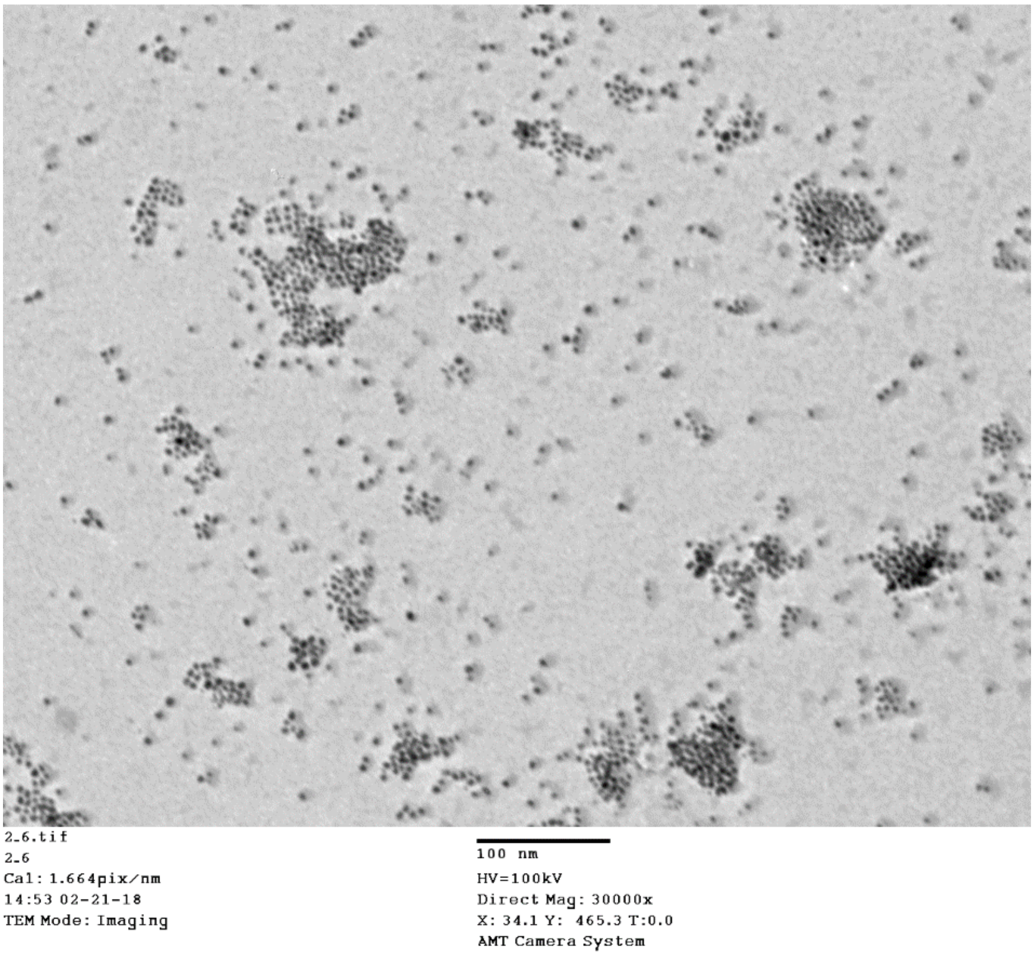

Figure S1. TEM image of the insoluble fraction after 18 h.

4. Kinetic Studies

4.1. Procedure for KIE experiments

The KIE was determined by comparing the initial reaction rates of the reactions with benzene and benzene-d_6. Both reactions were conducted using the above general procedure with the following modifications. After adding all the reagents, 3-bromotoluene (0.20 mmol) and dodecane (0.20 mmol) as an internal standard were added to a 10 mL Schlenk tube, and each tube was heated to 80 °C in an oil bath. At the required time period, each tube was immediately placed into an ice bath, and a small aliquot was withdrawn. Each aliquot was filtered through Celite and then analyzed by GC.

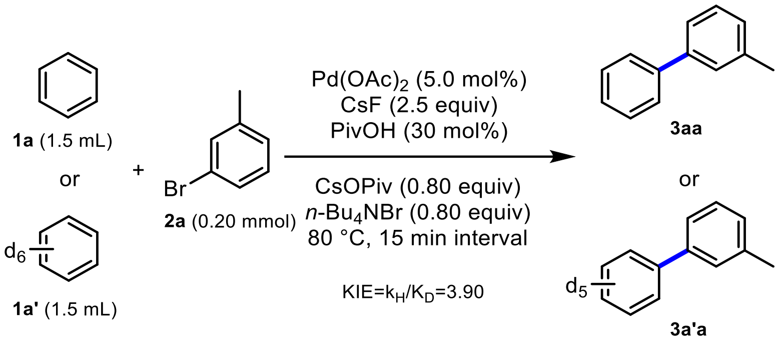

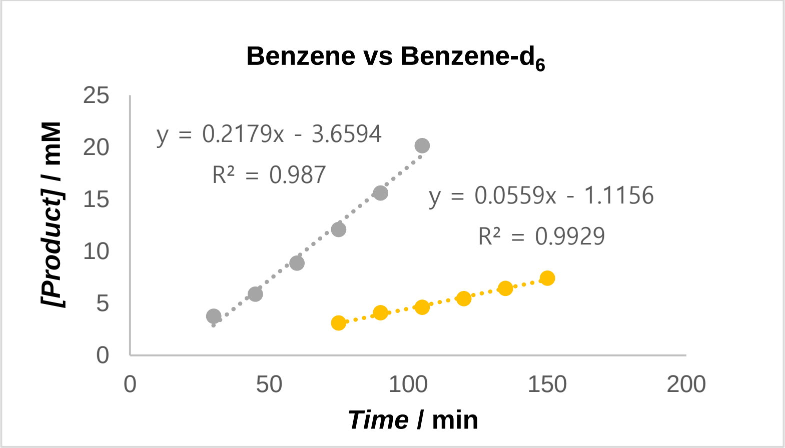

Figure S2. Initial rates of the C–H arylation of benzene and benzene-d_6

4.2. Kinetic data

4.2.1. Order in [Pd]

The order in [Pd] was determined by obtaining the initial rate of the C–H arylation with varying concentrations of Pd(OAc)$_2$. Each reaction was conducted using the general procedure with the following modifications. After adding all the reagents, varying amounts of Pd(OAc)$_2$ and dodecane (0.20 mmol) as an internal standard were added to a 10 mL Schlenk tube, and each tube was heated to 80 °C in an oil bath. At the required time period, each tube was immediately placed into an ice bath, and then a small aliquot was withdrawn. Each aliquot was filtered through Celite and then analyzed by GC.

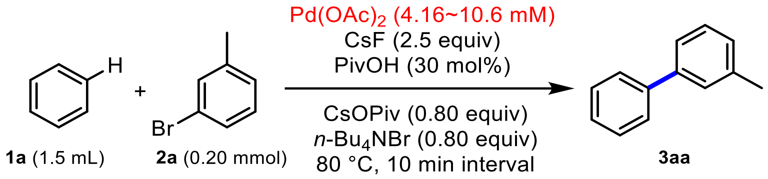

Table S5. Amounts of Pd(OAc)$_2$ and results used to determine the order in Pd(OAc)$_2$

Entry	Amount of Pd(OAc)$_2$			Rate (mM/min)
	mg	mmol	mM	
1	1.40	0.06236	4.16	0.0784
2	2.24	0.09978	6.65	0.1238
3	2.76	0.01229	8.20	0.1466
4	3.21	0.01430	9.53	0.2201
5	3.58	0.01595	10.6	0.2883

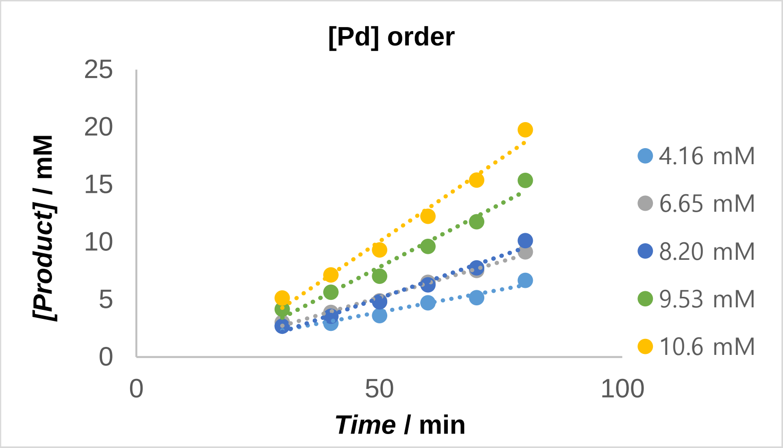

Figure S3. Initial rates of the C–H arylation with varying concentrations of $Pd(OAc)_2$

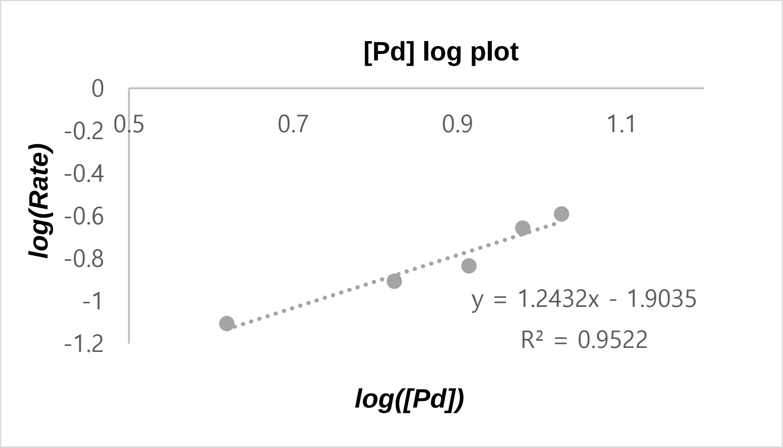

Figure S4. Log plot of initial rates with varying $Pd(OAc)_2$

4.2.2. Order in [ArBr]

The order in [ArBr] was determined by obtaining the initial rate of the C–H arylation with varying concentrations of 3-bromotoluene. Each reaction was conducted using the general procedure with the following modifications. After adding all the reagents, varying amounts of 3-bromotoluene and dodecane (0.20 mmol) as an internal standard were added to a 10 mL Schlenk tube, and each tube was heated to 80 °C in an oil bath. At the required time period, each tube was immediately placed into an ice bath, and then a small aliquot was withdrawn. Each aliquot was filtered through Celite and then analyzed by GC.

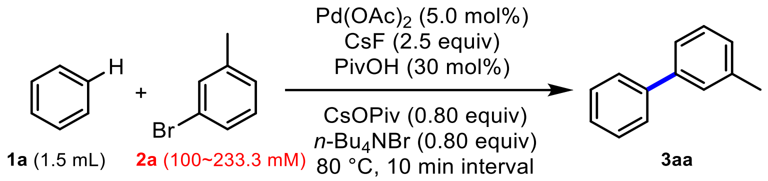

Table S6. Amounts of 3-bromotoluene and results used to determine the order in 3-bromotoluene

Entry	Amount of 3-bromotoluene			Rate (mM/min)
	μL	mmol	mM	
1	18	0.15	100	0.1469
2	24	0.20	133.3	0.2279
3	30	0.25	166.7	0.2047
4	36.3	0.30	200	0.1828
5	42.5	0.35	233.3	0.1185

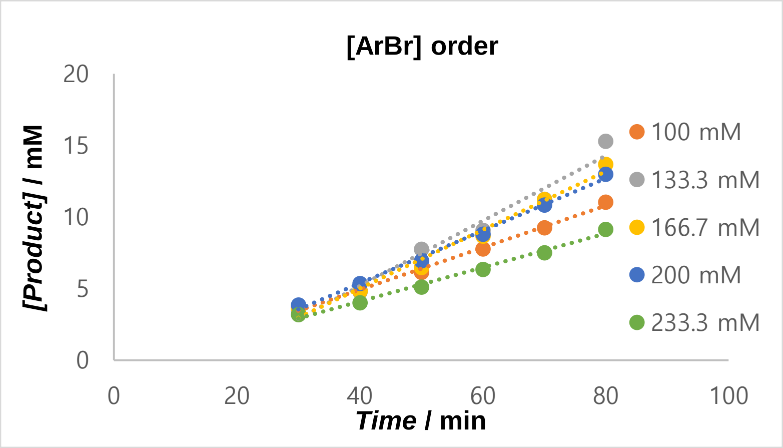

Figure S5. Initial rates of the C–H arylation with varying concentrations of 3-bromotoluene

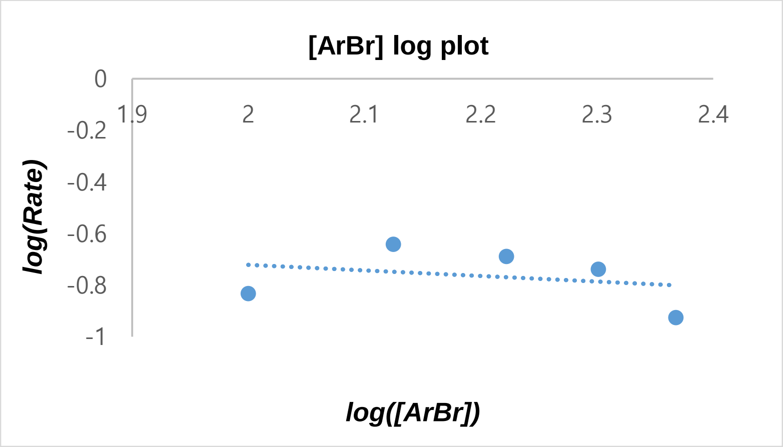

Figure S6. Log plot of initial rates with varying 3-bromotoluene

4.2.3. Competition Experiments

Set A: 3-bromotoluene (**2a**) vs. 4-bromobenzotrifluoride (**2q**)

The reaction was conducted by modifying the general procedure. Inside an argon-filled glove box, a 10 mL Schlenk tube was charged with cesium fluoride (2.5 equiv), cesium pivalate (0.80 equiv), palladium acetate (5.0 mol%), pivalic acid (0.30 equiv), *n*-tetrabutylammonium bromide (0.80 equiv), followed by benzene (1.5 mL), **2a** (0.20 mmol, 1.0 equiv), and **2q** (0.20 mmol, 1.0 equiv). The Schlenk tube was sealed and heated to 80 °C for 6 h. Upon completion of the reaction, the mixture was cooled to room temperature. It was analyzed by GC using dodecane as an internal standard.

Set B: 3-bromotoluene (**2a**) vs. 4-bromoanisole (**2g**)

The reaction was conducted according to the above procedure using **2a** (0.20 mmol, 1.0 equiv) and **2g** (0.20 mmol, 1.0 equiv) as competing substrates. Fluorobenzene (1.5 mL) was used instead of benzene.

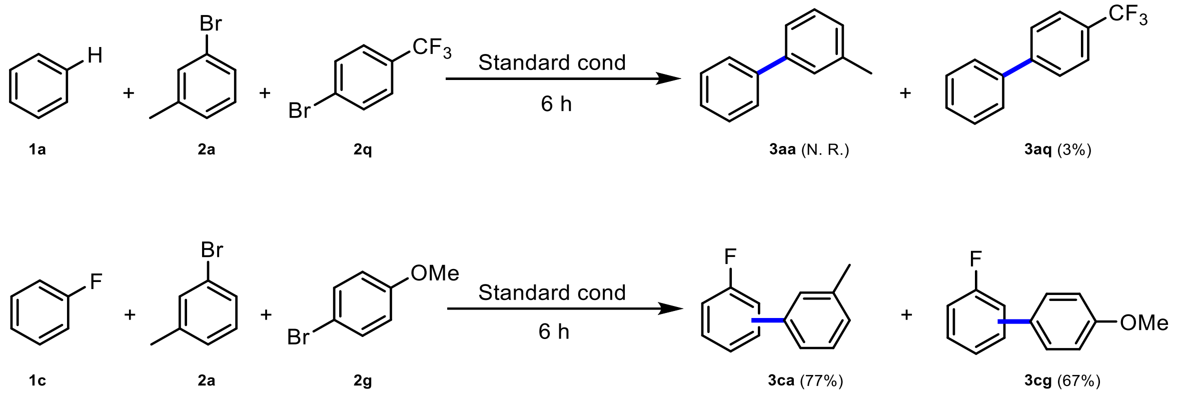

Scheme S2. Competition Experiments

5. Gram-scale reaction

General procedure for arylation reaction with ammonium salt

A Schlenk tube (500 mL) was charged with 3-bromotoluene (10 mmol), cesium fluoride (2.5 equiv), cesium pivalate (0.80 equiv), palladium acetate (5.0 mol%), pivalic acid (30 mol%), tetra-*n*-butylammonium bromide (0.80 equiv), and benzene (75 mL) in an argon-filled glove box. The reaction vessel was placed in an oil bath and heated to 80 °C. After 18 h, the reaction was cooled to room temperature, and the crude reaction mixture was concentrated using a rotary evaporator. The residue was purified by column chromatography to afford the desired product.

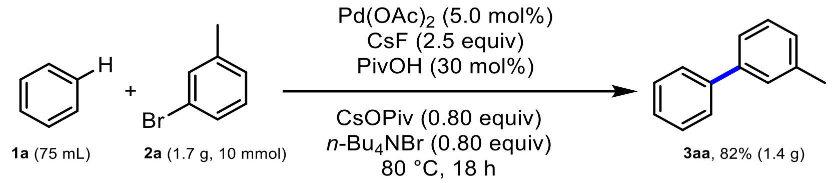

1a (75 mL) **2a** (1.7 g, 10 mmol) **3aa**, 82% (1.4 g)

Scheme S3. Gram-scale reaction of direct C–H arylation

6. Synthesis of compounds used in homo/heterogeneity studies

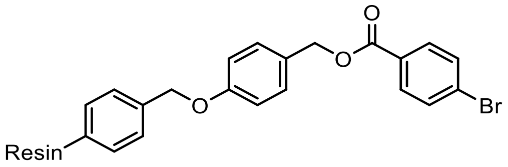

Resin-ArBr (4)

The synthesis was conducted by modifying a procedure used for an analogous complex.[2] A 50 mL Schlenk tube was charged with Wang resin (1.75 g, 2.0 mmol), 4-bromobenzoic acid (1.21 g, 3.0 equiv), *N, N'*-diisopropylcarbodiimide (0.93 mL, 3.0 equiv), and 4-dimethylaminopyridine (122.2 mg, 0.50 equiv) in CH_2Cl_2 (20 mL). The reaction was conducted under room temperature for 18 h. After the reaction, the crude mixture was filtered and washed by CH_3OH, THF, and CH_2Cl_2. The resulting pale yellow solid was dried under vacuum (1.90 g, 90%). The purity of the complex was confirmed by elemental analysis. Anal. Found. for **4** (based on Wang resin): C, 80.70; H, 6.43; O, 4.91.

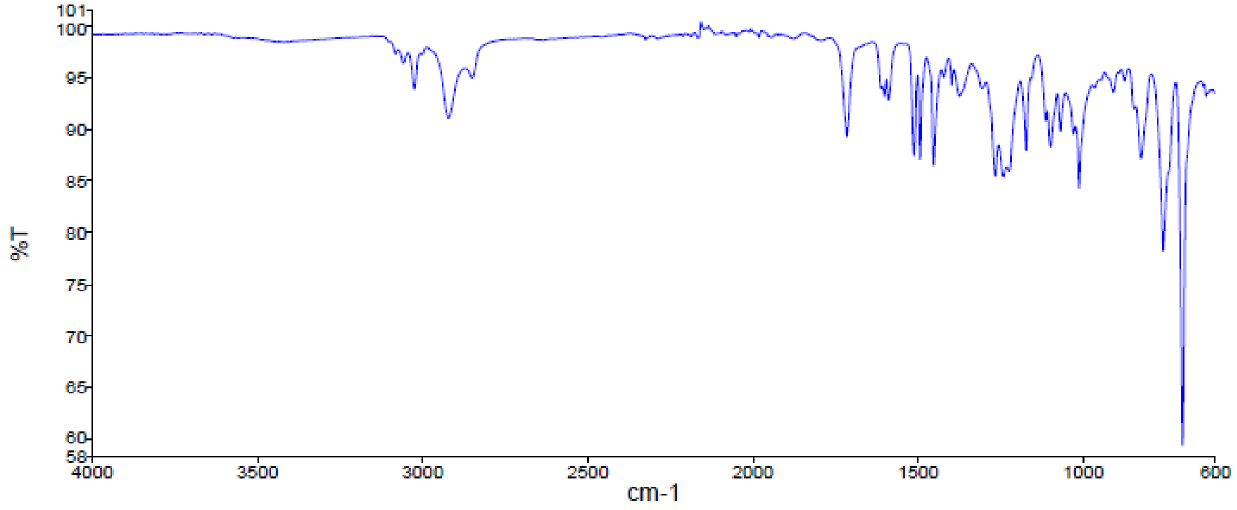

Figure S7. IR spectrum of **4**

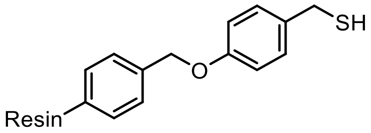

Resin-SH (5)

Synthesis was conducted by modifying a procedure used for an analogous complex.[3] A 50 mL Schlenk tube was charged with brominated Wang resin (625.0 mg, 0.50

mmol), and thiourea (380.6 mg, 10 equiv) in 1,4-dioxane/ethanol (5.0 mL/5.0 mL), and stirred under reflux for 27 h. Piperidine (0.49 mL, 10 equiv) was added and further stirring was done for 5 h. After the reaction, the crude mixture was diluted by adding H_2O and filtered, washed by H_2O, acetone, and CH_3OH. The resulting yellow solid was dried under vacuum (610.8 mg, >99%). The purity of the complex was confirmed by elemental analysis. Anal. Found. for **5** (based on Wang brominated resin): C, 84.79; H, 7.49; O, 1.59; N, 2.08; S, 2.41.

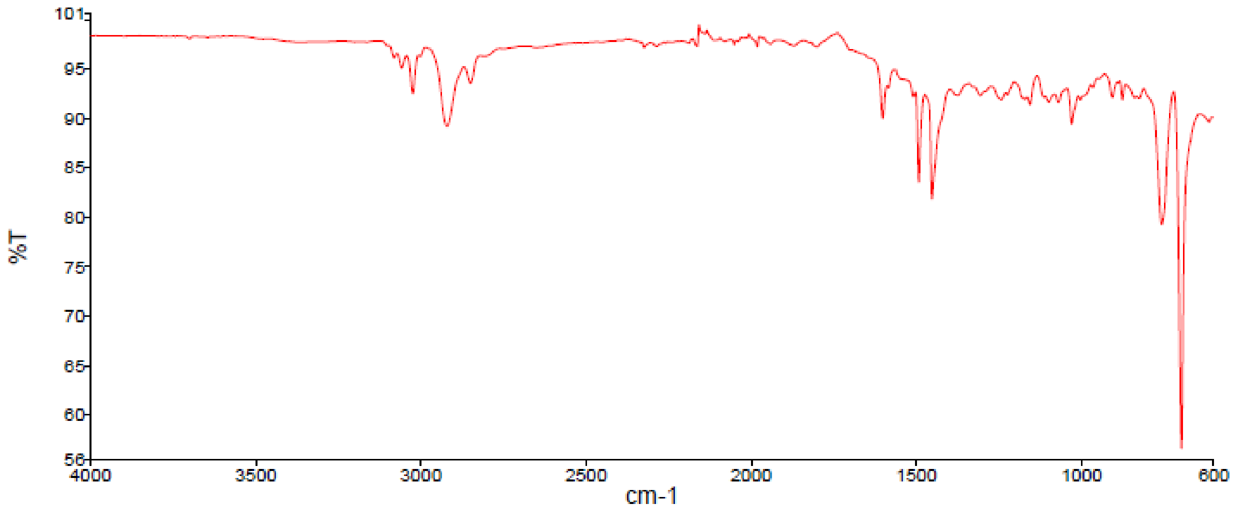

Figure S8. IR spectrum of **5**

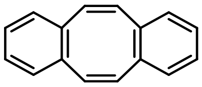

Dibenzo[*a*,*e*]cyclooctatetraene (7)

The compound was synthesized by 3 steps referring to the literature.[4]

7. Characterization of products

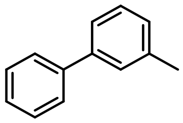

3-methylbiphenyl (3aa)

Colorless oil. The compound was identified by spectral comparison with literature data.[5]

^{1}H NMR (400 MHz, $CDCl_3$) δ 7.60 (d, *J* = 7.6 Hz, 2H), 7.46 – 7.40 (m, 4H), 7.34 (t, *J* = 7.4 Hz, 2H), 7.18 (d, *J* = 7.4 Hz, 1H), 2.43 (s, 3H).

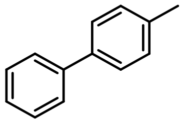

4-methylbiphenyl (3ab)

White solid. The compound was identified by spectral comparison with literature data.[6]

^{1}H NMR (499 MHz, $CDCl_3$) δ 7.50 (d, *J* = 7.6 Hz, 2H), 7.41 (d, *J* = 7.5 Hz, 2H), 7.38 – 7.32 (m, 2H), 7.27 – 7.22 (m, 1H), 7.19 – 7.14 (m, 2H), 2.31 (s, 3H).

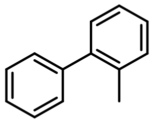

2-methylbiphenyl (3ac)

Colorless oil. The compound was identified by spectral comparison with literature data.[7]

^{1}H NMR (499 MHz, $CDCl_3$) δ 7.43 – 7.39 (m, 2H), 7.35 – 7.32 (m, 3H), 7.28 – 7.27 (m, 2H), 7.25 – 7.23 (m, 2H), 2.28 (s, 3H).

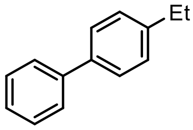

4-ethylbiphenyl (3ad)

White solid. The compound was identified by spectral comparison with literature data.[8]

^{1}H NMR (400 MHz, $CDCl_3$) δ 7.62 (d, *J* = 8.1 Hz, 2H), 7.56 (d, *J* = 7.9 Hz, 2H), 7.46 (t, *J* = 7.6 Hz, 2H), 7.35 (t, J = 7.4 Hz, 1H), 7.31 (d, J = 7.9 Hz, 2H), 2.73 (q, *J* = 7.6 Hz,

2H), 1.31 (t, J = 7.6 Hz, 3H).

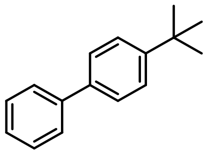

4-*tert*-butylbiphenyl (3ae)

Colorless oil. The compound was identified by spectral comparison with literature data.[9]

^{1}H NMR (400 MHz, $CDCl_3$) δ 7.62 – 7.52 (m, 4H), 7.49 – 7.40 (m, 4H), 7.36 – 7.31 (m, 1H), 1.37 (s, 9H).

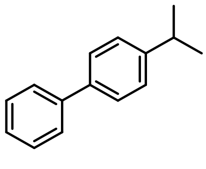

4-isopropylbiphenyl (3af)

Colorless oil. The compound was identified by spectral comparison with literature data.[10]

^{1}H NMR (400 MHz, $CDCl_3$) δ 7.57 (d, J = 5.8 Hz, 2H), 7.52 (d, J = 4.8 Hz, 2H), 7.45 – 7.38 (m, 2H), 7.35 – 7.27 (m, 3H), 3.01 – 2.89 (m, 1H), 1.30 – 1.27 (m, 6H).

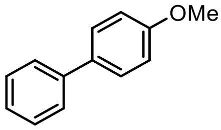

4-methoxybiphenyl (3ag)

White solid. The compound was identified by spectral comparison with literature data.[5]

^{1}H NMR (499 MHz, $CDCl_3$) δ 7.58 – 7.52 (m, 4H), 7.42 (t, J = 7.7 Hz, 2H), 7.31 (t, J = 7.4 Hz, 1H), 6.99 (d, J = 8.7 Hz, 2H), 3.86 (s, 3H).

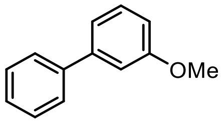

3-methoxybiphenyl (3ah)

White solid. The compound was identified by spectral comparison with literature data.[5]

^{1}H NMR (400 MHz, $CDCl_3$) δ 7.61 – 7.58 (m, 2H), 7.44 (t, *J* = 7.5 Hz, 2H), 7.39 – 7.33 (m, 2H), 7.21 – 7.17 (m, 1H), 7.14 – 7.12 (m, 1H), 6.90 (dd, *J* = 7.8, 2.1 Hz, 1H), 3.87 (s, 3H).

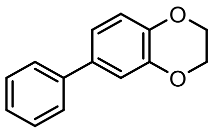

6-phenyl-2,3-dihydrobenzo[*b*][1,4]dioxine (3ai)

White solid. The compound was identified by spectral comparison with literature data.[11]

^{1}H NMR (499 MHz, $CDCl_3$) δ 7.55 – 7.52 (m, 2H), 7.41 (t, *J* = 7.7 Hz, 2H), 7.30 (t, *J* = 6.9 Hz, 1H), 7.12 (d, *J* = 2.1 Hz, 1H), 7.09 (dd, *J* = 8.3, 2.2 Hz, 1H), 6.93 (d, *J* = 8.3 Hz, 1H), 4.30 (s, 4H).

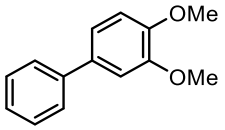

3,4-dimethoxybiphenyl (3aj)

White solid. The compound was identified by spectral comparison with literature data.[12]

^{1}H NMR (499 MHz, $CDCl_3$) δ 7.57 (d, *J* = 7.3 Hz, 2H), 7.43 (t, *J* = 7.7 Hz, 2H), 7.33 (t, *J* = 7.3 Hz, 1H), 7.16 (dd, *J* = 8.2, 1.9 Hz, 1H), 7.12 (d, *J* = 1.8 Hz, 1H), 6.96 (d, *J* = 8.3 Hz, 1H), 3.96 (s, 3H), 3.93 (s, 3H).

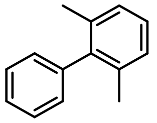

2,6-dimethylbiphenyl (3ak)

Colorless oil. The compound was identified by spectral comparison with literature data.[13] ^{1}H NMR (499 MHz, $CDCl_3$) δ 7.43 (t, *J* = 7.5 Hz, 2H), 7.34 (t, *J* = 7.4 Hz, 1H), 7.18 – 7.11 (m, 5H), 2.04 (s, 6H).

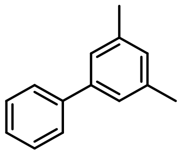

3,5-dimethylbiphenyl (3al)

Colorless oil. The compound was identified by spectral comparison with literature data.[14]

^{1}H NMR (400 MHz, $CDCl_3$) δ 7.58 (d, J = 8.2 Hz, 2H), 7.43 (t, J = 7.5 Hz, 2H), 7.33 (t, J = 7.3 Hz, 1H), 7.22 (s, 2H), 7.01 (s, 1H), 2.39 (s, 6H).

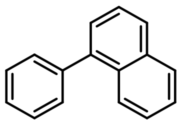

1-phenylnaphthalene (3am)

Colorless oil. The compound was identified by spectral comparison with literature data.[5]

^{1}H NMR (400 MHz, $CDCl_3$) δ 7.91 (d, J = 8.3 Hz, 2H), 7.87 (d, J = 8.2 Hz, 1H), 7.54 (d, J = 8.0 Hz, 1H), 7.49 (t, J = 5.9 Hz, 5H), 7.44 (d, J = 1.3 Hz, 3H).

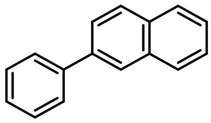

2-phenylnaphthalene (3an)

White solid. The compound was identified by spectral comparison with literature data.[5]

^{1}H NMR (499 MHz, $CDCl_3$) δ 8.06 (s, 1H), 7.95 – 7.86 (m, 3H), 7.79 – 7.72 (m, 3H), 7.54 – 7.49 (m, 4H), 7.41 – 7.38 (m, 1H).

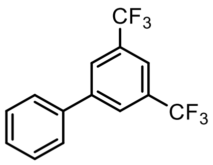

3,5-bis(trifluoromethyl)-biphenyl (3ao)

Colorless oil. The compound was identified by spectral comparison with literature data.[5]

^{1}H NMR (400 MHz, $CDCl_3$) δ 8.02 (s, 2H), 7.86 (s, 1H), 7.62 (d, J = 7.1 Hz, 2H), 7.53

– 7.44 (m, 3H).

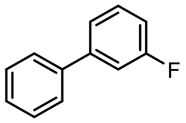

3-fluorobiphenyl (3ap)

Colorless oil. The compound was identified by spectral comparison with literature data.[15]

^{1}H NMR (499 MHz, $CDCl_3$) δ 7.55 – 7.48 (m, 2H), 7.38 (t, J = 7.6 Hz, 2H), 7.34 – 7.29 (m, 3H), 7.22 (d, J = 10.2, 1.5 Hz, 1H), 7.00 – 6.94 (m, 1H).

$PdBr_4$(*n*-$Bu_4N)_2$

Red solid.

^{1}H NMR (400 MHz, $CDCl_3$) δ 3.53 – 3.43 (m, 8H), 1.82 – 1.71 (m, 8H), 1.60 – 1.48 (m, 8H), 1.03 (t, *J* = 7.3 Hz, 12H); ^{13}C NMR (400 MHz, $CDCl_3$) δ 59.43, 24.54, 20.04, 14.01. The purity of the complex was confirmed by elemental analysis. Anal. Calcd. for $C_{32}H_{72}Br_4N_2Pd$: C, 42.19; H, 7.97; Br, 35.08; N, 3.08; Found: C, 42.15; H, 8.08; N, 3.09.

The yields and selectivities of arene substrate scopes were determined by GC using dodecane as an internal standard.

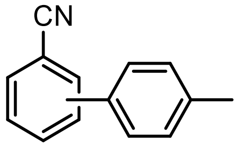

Mixture of 4'-methyl-[1,1'-biphenyl]-2-carbonitrile, 4'-methyl-[1,1'-biphenyl]-3-carbonitrile, 4'-methyl-[1,1'-biphenyl]-4-carbonitrile (3bb)

Dodecane: RT=7.26 (min)

4'-methyl-[1,1'-biphenyl]-2-carbonitrile: RT=11.41 (min)

4'-methyl-[1,1'-biphenyl]-3-carbonitrile: RT=11.77 (min)

4'-methyl-[1,1'-biphenyl]-4-carbonitrile: RT=11.90 (min)

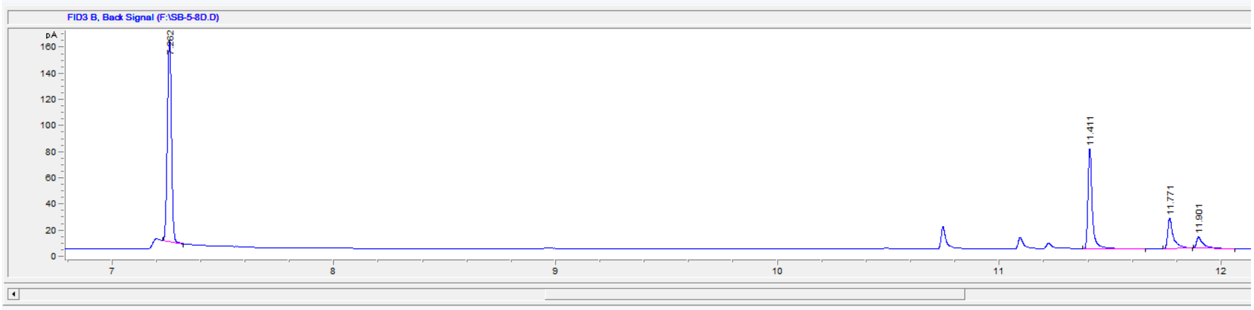

#	Time	Area	Height	Width	Area%	Symmetry
1	7.262	199	154.3	0.0202	55.229	0.999
2	11.411	102.6	76.2	0.0197	28.488	0.616
3	11.771	40.8	23.1	0.025	11.324	0.488
4	11.901	17.9	8.5	0.0289	4.959	0.407

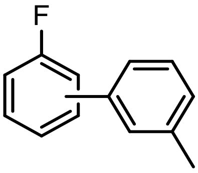

Mixture of 2-fluoro-3'-methyl-1,1'-biphenyl, 3-fluoro-3'-methyl-1,1'-biphenyl, 4'-fluoro-3-methyl-1,1'-biphenyl (3ca)

Dodecane: RT=7.26 (min)

2-fluoro-3'-methyl-1,1'-biphenyl: RT= 9.52 (min)

3-fluoro-3'-methyl-1,1'-biphenyl: RT= 9.60 (min)

4-fluoro-3'-methyl-1,1'-biphenyl: RT= 9.63 (min)

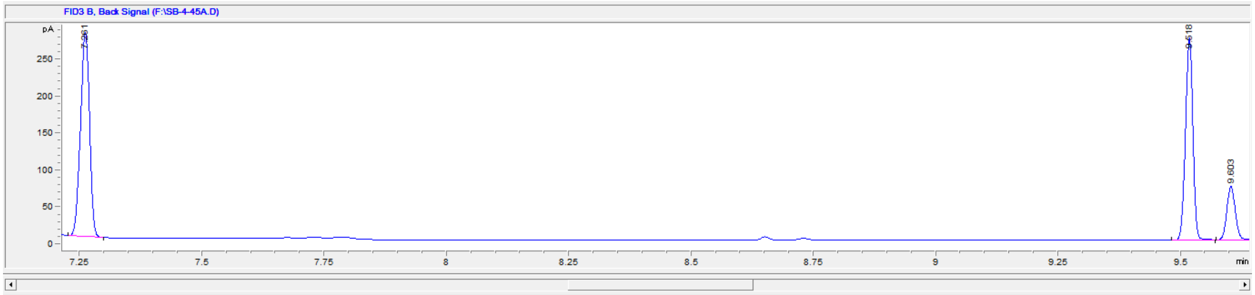

#	Time	Area	Height	Width	Area%	Symmetry
1	7.261	355.2	275.9	0.0202	48.787	1.106
2	9.518	287.3	271.9	0.0167	39.454	1.077
3	9.603	85.6	71.7	0.0191	11.759	0.881

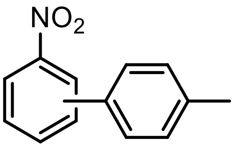

Mixture of 4'-methyl-2-nitro-1,1'-biphenyl, 4'-methyl-3-nitro-1,1'-biphenyl, 4-

methyl-4'-nitro-1,1'-biphenyl (3db)

Dodecane: RT=7.26 (min)

4'-methyl-2-nitro-1,1'-biphenyl: RT=11.67 (min)

4'-methyl-3-nitro-1,1'-biphenyl: RT=12.45 (min)

4'-methyl-4-nitro-1,1'-biphenyl: RT=12.62 (min)

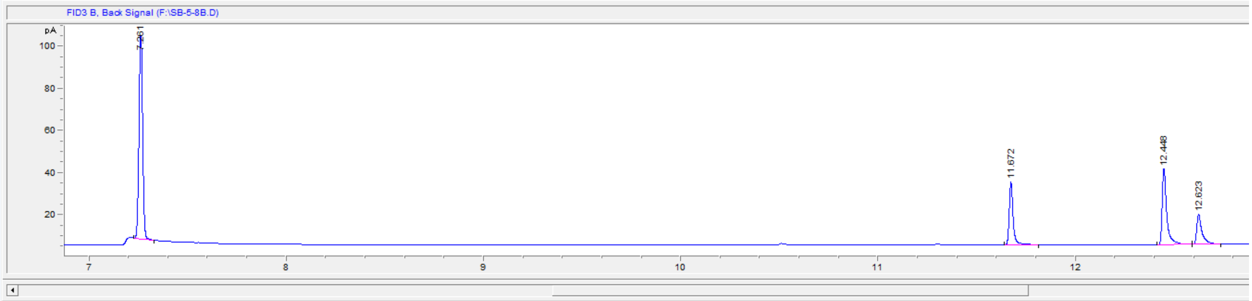

#	Time	Area	Height	Width	Area%	Symmetry
1	7.261	125.8	96.5	0.02	50.467	0.952
2	11.672	40	29.7	0.0197	16.057	0.702
3	12.448	57.1	36	0.0237	22.910	0.581
4	12.623	26.3	14.2	0.0272	10.566	0.472

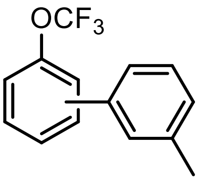

Mixture of 3'-methyl-2-(trifluoromethoxy)-1,1'-biphenyl, 3-methyl-3'-(trifluoromethoxy)-1,1'-biphenyl, 3-methyl-4'-(trifluoromethoxy)-1,1'-biphenyl (3ea)

Dodecane: RT=7.26 (min)

3'-methyl-2-(trifluoromethoxy)-1,1'-biphenyl: 8.95 (min)

3'-methyl-3-(trifluoromethoxy)-1,1'-biphenyl: 9.56 (min)

3'-methyl-4-(trifluoromethoxy)-1,1'-biphenyl: 9.70 (min)

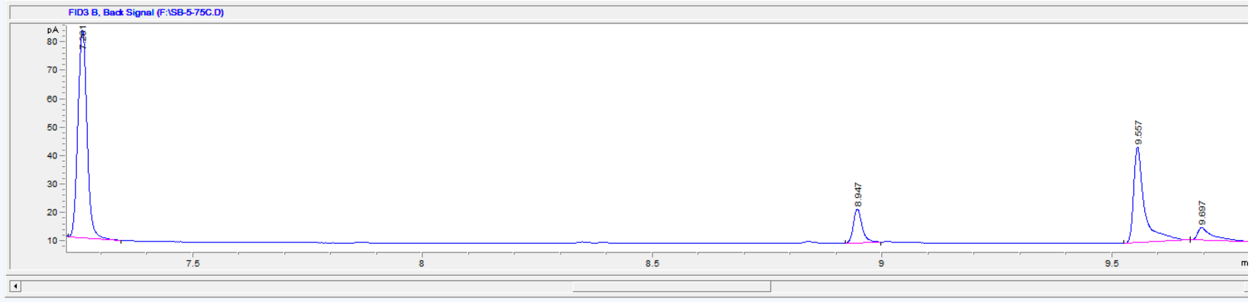

#	Time	Area	Height	Width	Area%	Symmetry
1	7.261	100.5	72.8	0.0217	55.221	0.936
2	8.947	15.7	11.9	0.0202	8.622	0.767
3	9.557	55.1	33.6	0.024	30.303	0.495
4	9.697	10.7	4.4	0.033	5.854	0.319

II. References

[1] R. H. Crabtree, *Chem. Rev.* **2012**, *112*, 1536-1554.

[2] A. La-Venia, S. A. Testero, M. P. Mischne, E. G. Mata, *Org. Biomol. Chem.* **2012**, *10*, 2514-2517.

[3] A. Lezzi, S. Cobianco, A. Roggero, *J. Polym. Sci., Part A.* **1994**, *32*, 1877-1883.

[4] S. Chaffins, M. Brettreich, F. Wudl, *Synthesis* **2002**, *9*, 1191-1194.

[5] J. Kim, S. H. Hong, *ACS Catal.* **2017**, *7*, 3336-3343.

[6] M. J. Iglesias, A. Prieto, M. C. Nicasio, *Org. Lett.* **2012**, *14*, 4318-4321.

[7] X. Li, T. Zhu, Z. Shao, Y. Li, H. Chang, W. Gao, Y. Zhang, W. Wei, *Tetrahedron* **2016**, *72*, 69-75.

[8] H. Zhang, C. Wang, Z. Li, Z. Wang, *Tetrahedron Lett.* **2015**, *56*, 5371-5376.

[9] R.-J. Tang, T. Milcent, B. Crousse, *J. Org. Chem.* **2018**, *83*, 930-938.

[10] N. S. Mahajani, J. D. Chisholm, *J. Org. Chem.* **2018**, *83*, 4131-4139.

[11] J. W. Fyfe, N. J. Fazakerley, A. J. Watson, *Angew. Chem. Int. Ed.* **2017**, *56*, 1249-1253.

[12] C. L. Ciana, R. J. Phipps, J. R. Brandt, F. M. Meyer, M. J. Gaunt, *Angew. Chem.* **2011**, *123*, 478-482.

[13] O. Y. Yuen, C. M. So, H. W. Man, F. Y. Kwong, *Chem. Eur. J.* **2016**, *22*, 6471-6476.

[14] K. Tanimoro, M. Ueno, K. Takeda, M. Kirihata, S. Tanimori, *J. Org. Chem.* **2012**, *77*, 7844-7849.

[15] C. Pan, J. Zhu, R. Chen, J.-T. Yu, *Org. Biomol. Chem.* **2017**, *15*, 6467-6469.

III. NMR spectra

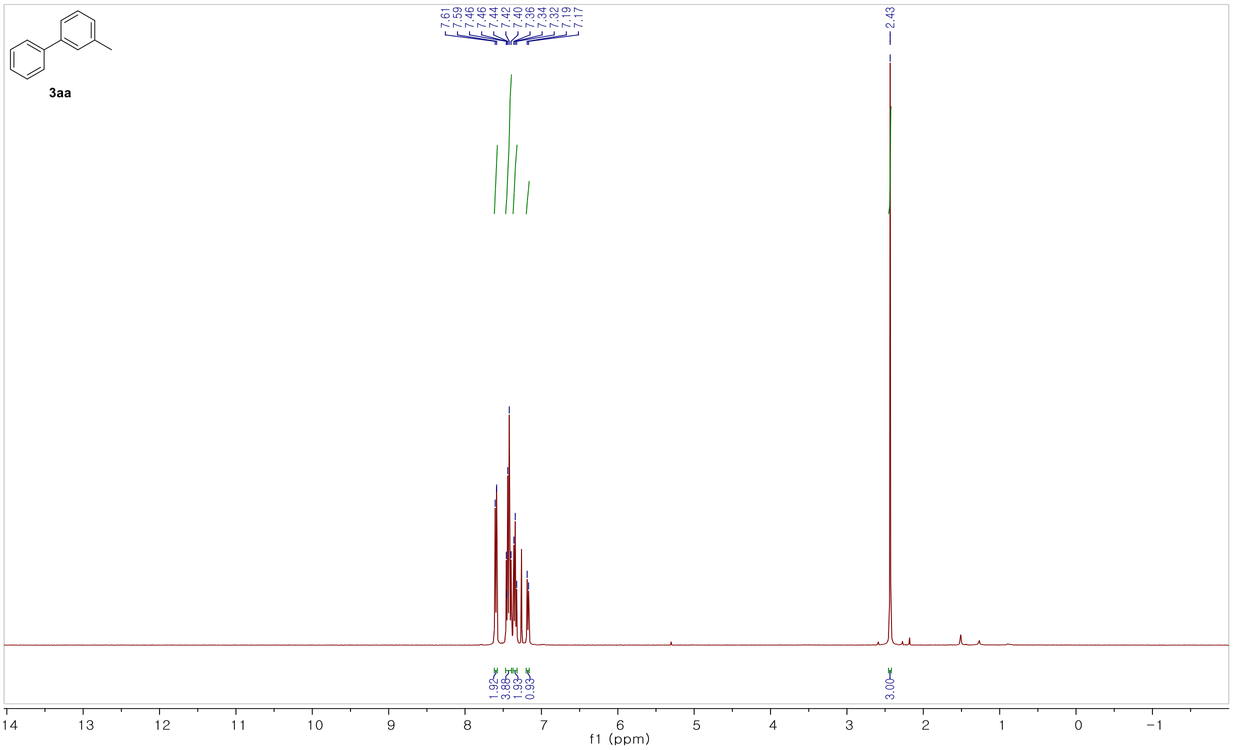

3aa

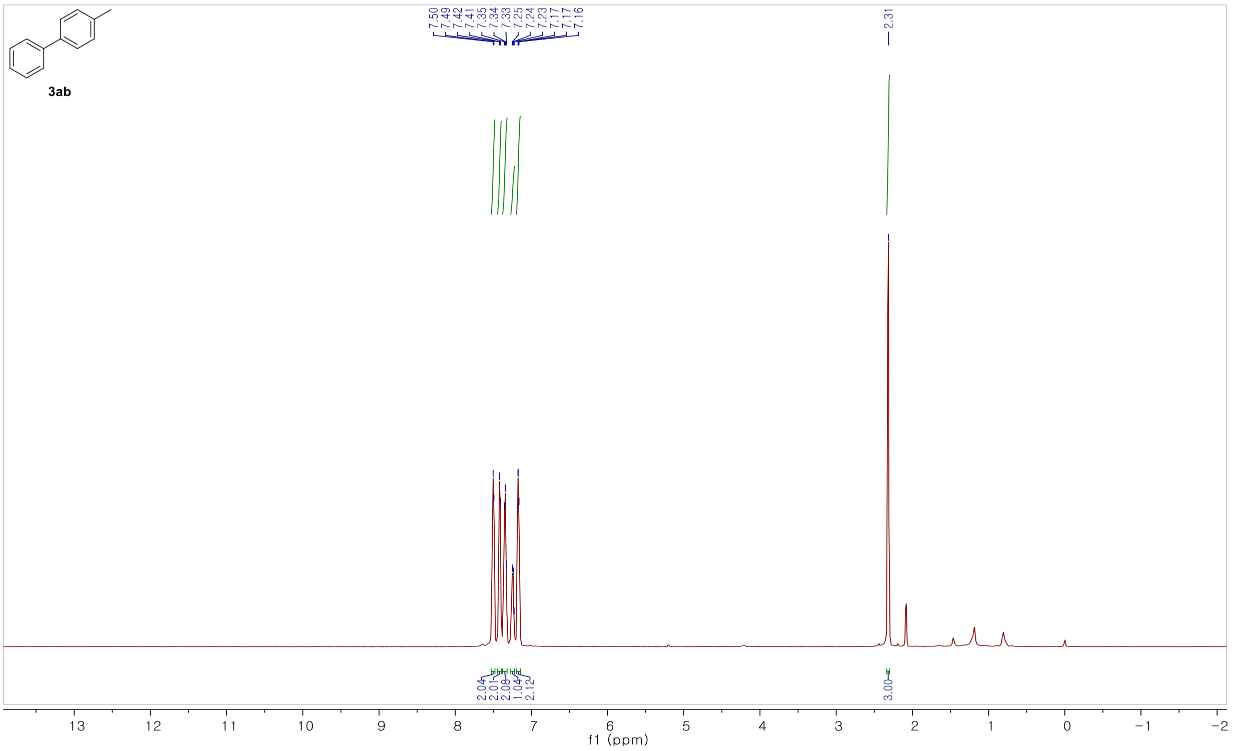

3ab

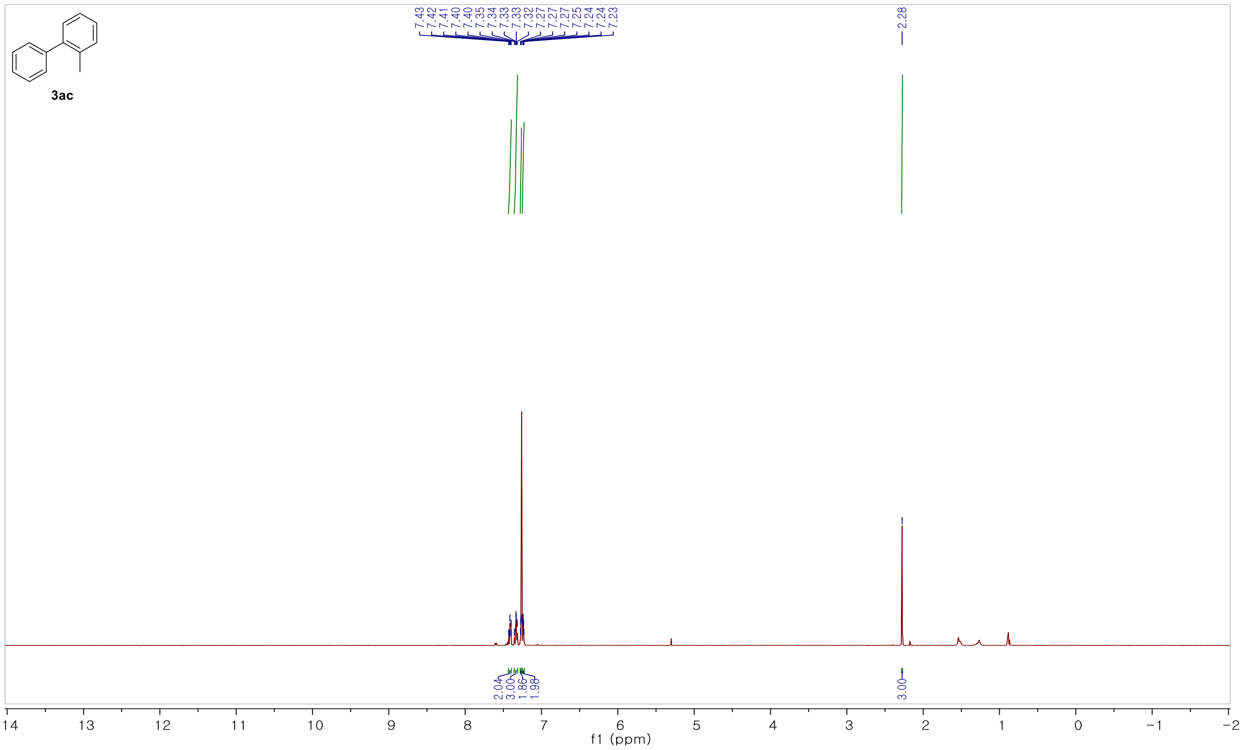

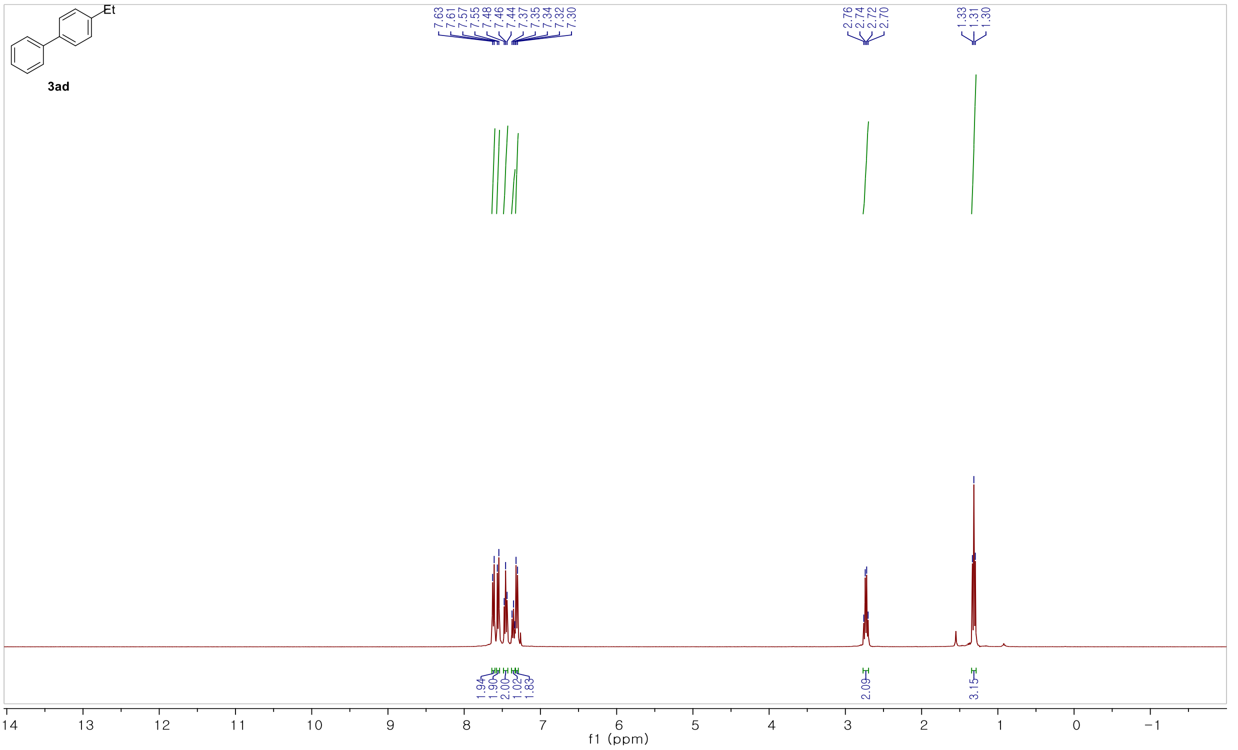

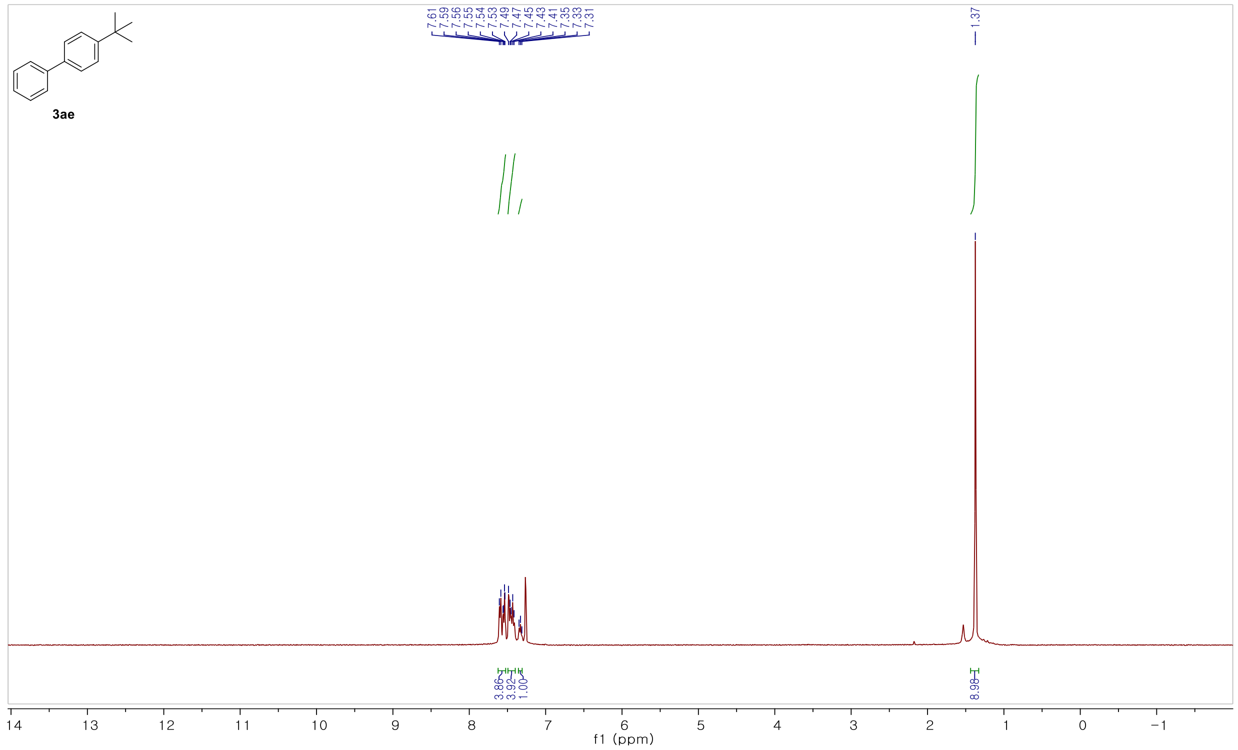
3ae
7.61 7.59 7.56 7.55 7.54 7.53 7.49 7.47 7.45 7.43 7.41 7.35 7.33 7.31
1.37
3.86 3.92 1.00
8.98
f1 (ppm)

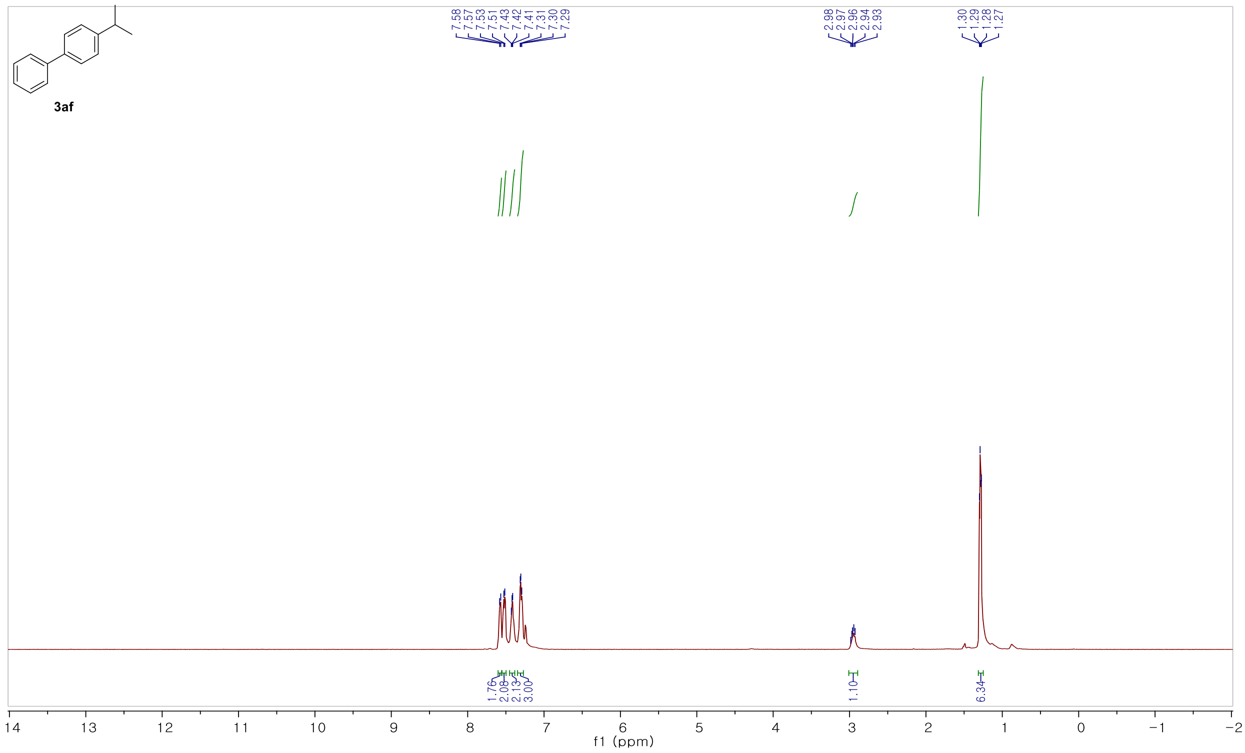
3af
7.58 7.57 7.53 7.51 7.43 7.42 7.41 7.31 7.30 7.29
2.98 2.97 2.96 2.94 2.93
1.30 1.29 1.28 1.27
1.76 2.08 2.13 3.00
1.10
6.34
f1 (ppm)

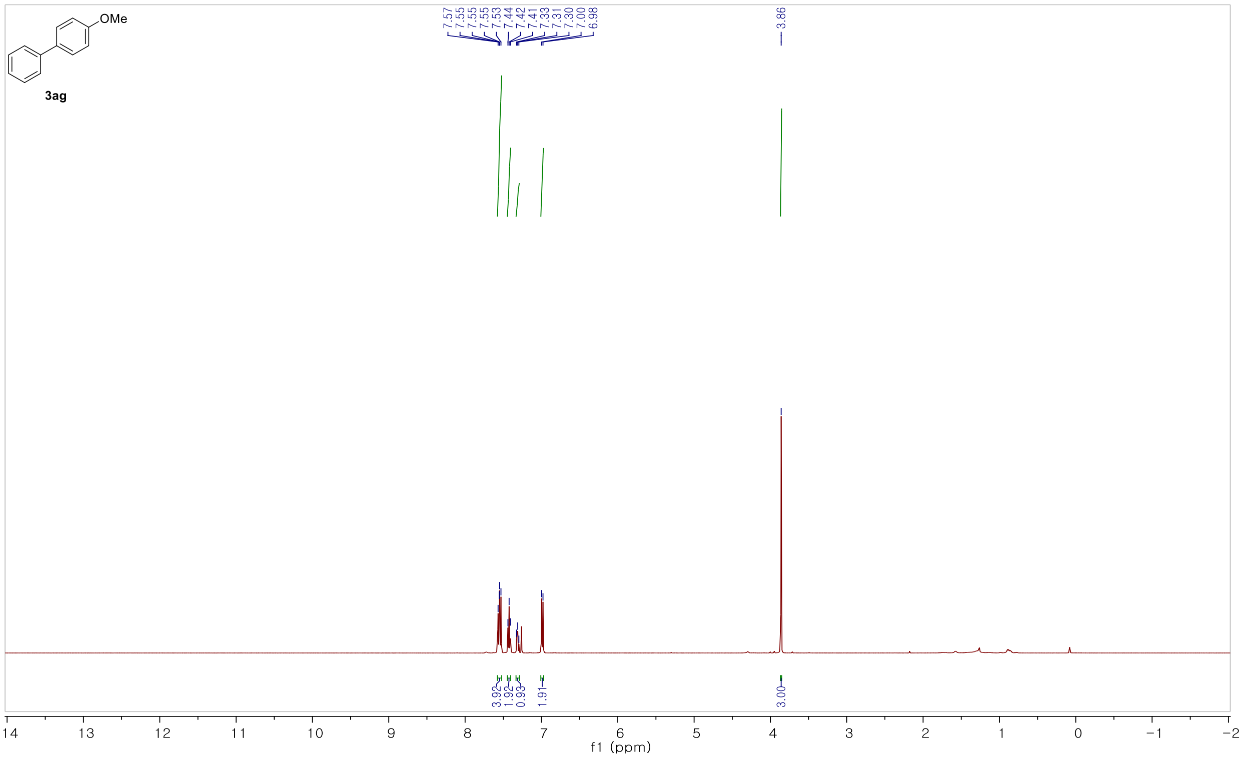

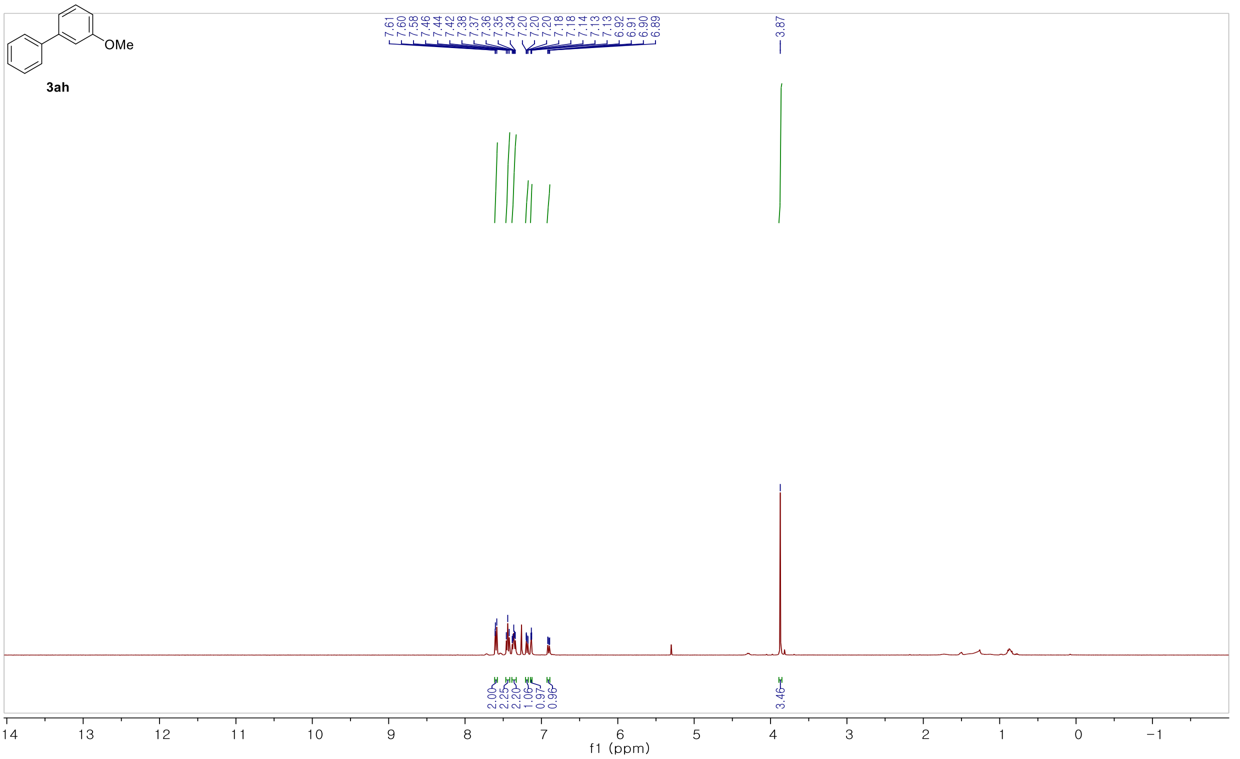

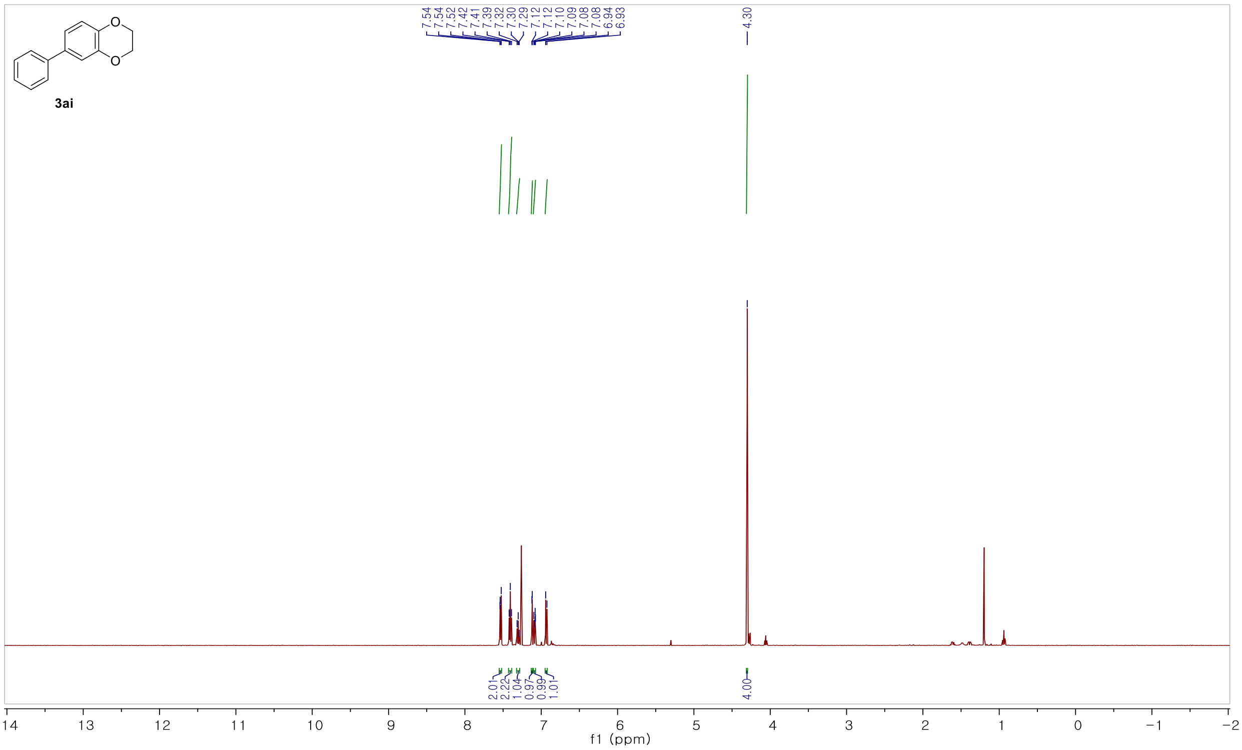
O
O
3ai
7.54
7.54
7.52
7.42
7.41
7.39
7.32
7.30
7.29
7.12
7.12
7.10
7.09
7.08
7.08
6.94
6.93
4.30
2.01
2.22
1.04
0.97
0.99
1.01
4.00
14
13
12
11
10
9
8
7
6
5
4
3
2
1
0
−1
−2
f1 (ppm)

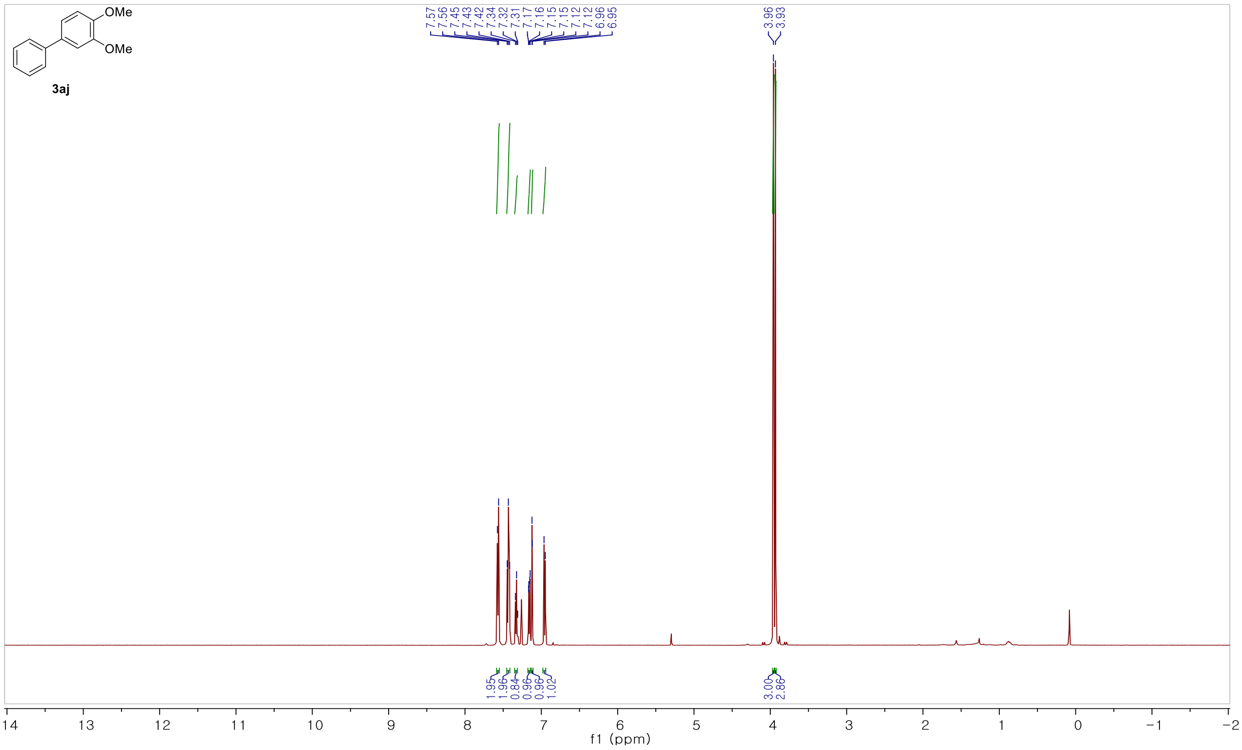
OMe
OMe
3aj
7.57
7.56
7.45
7.43
7.42
7.34
7.32
7.31
7.17
7.16
7.15
7.12
6.96
6.95
3.96
3.93
1.95
1.96
0.84
0.96
0.96
1.02
3.00
2.86
14
13
12
11
10
9
8
7
6
5
4
3
2
1
0
−1
−2
f1 (ppm)

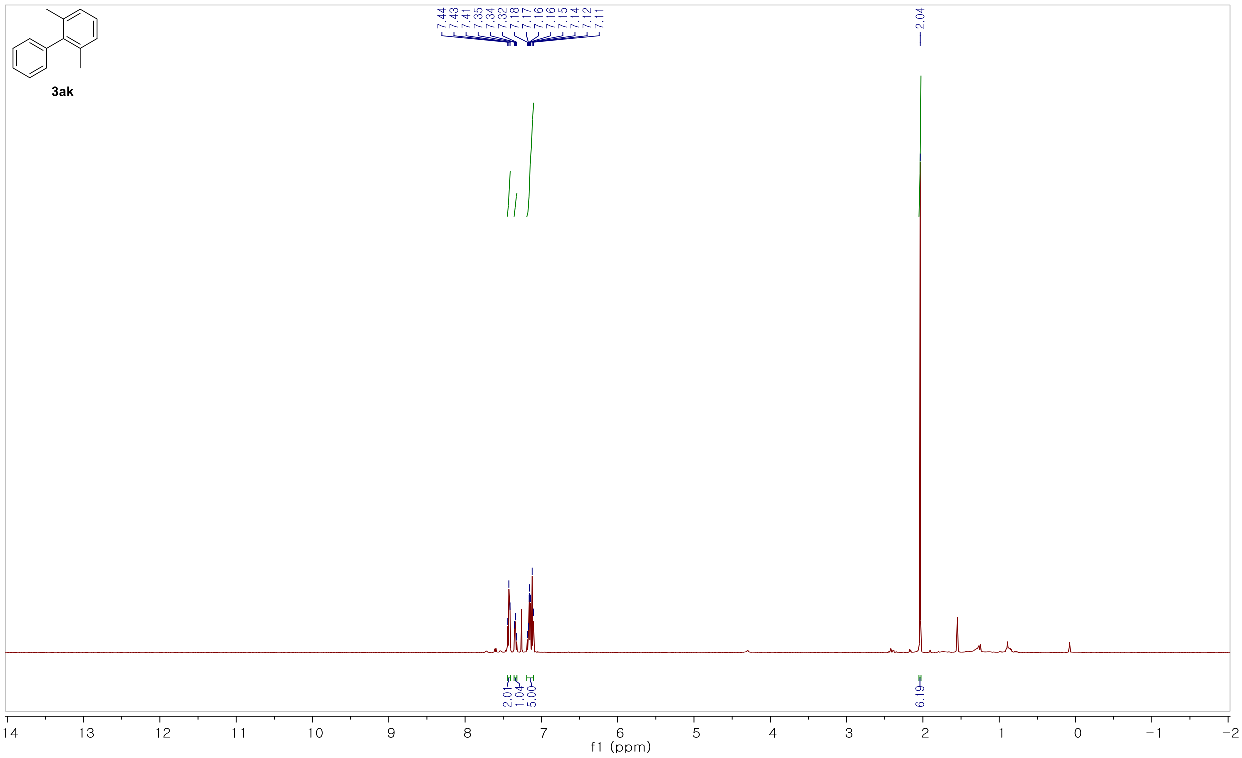

3ak
7.44
7.43
7.41
7.35
7.34
7.32
7.18
7.17
7.16
7.16
7.15
7.14
7.12
7.11
2.04
2.01
1.04
5.00
6.19
14 13 12 11 10 9 8 7 6 5 4 3 2 1 0 −1 −2
f1 (ppm)

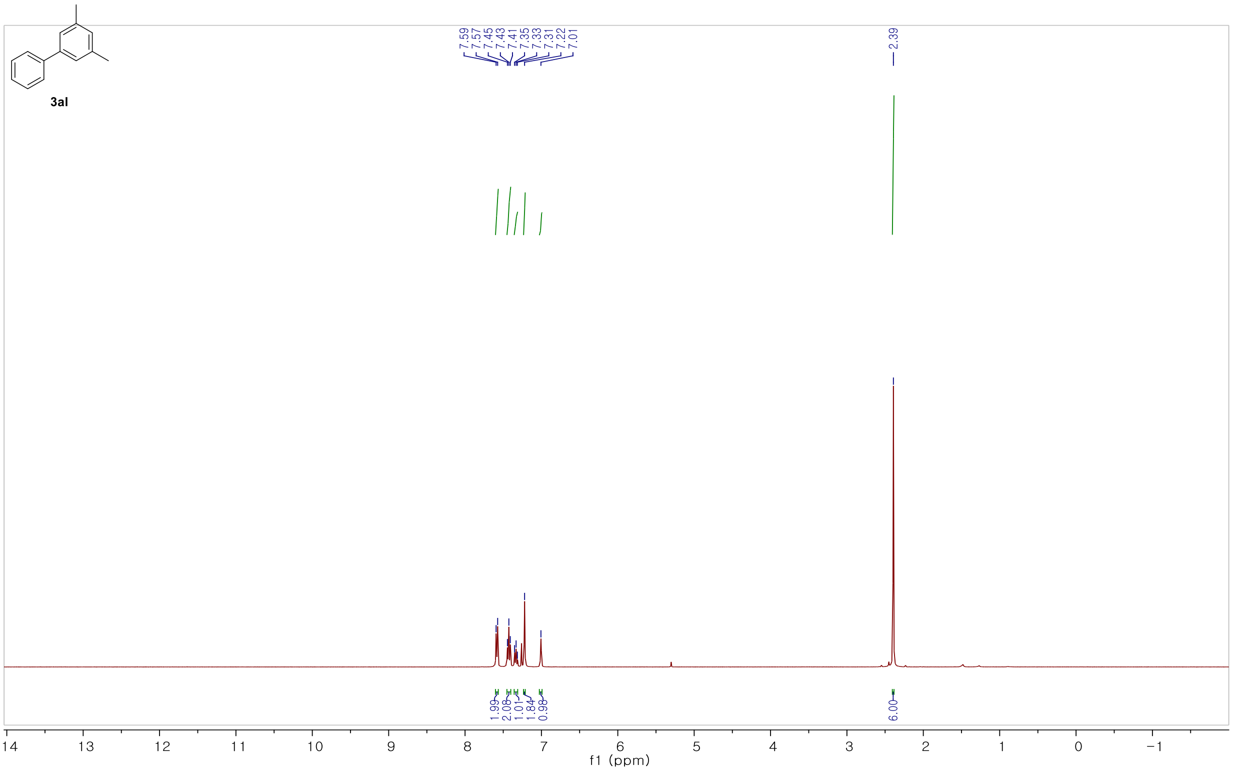

3al
7.59
7.57
7.45
7.43
7.41
7.35
7.33
7.31
7.22
7.01
2.39
1.99
2.08
1.01
1.84
0.98
6.00
14 13 12 11 10 9 8 7 6 5 4 3 2 1 0 −1
f1 (ppm)

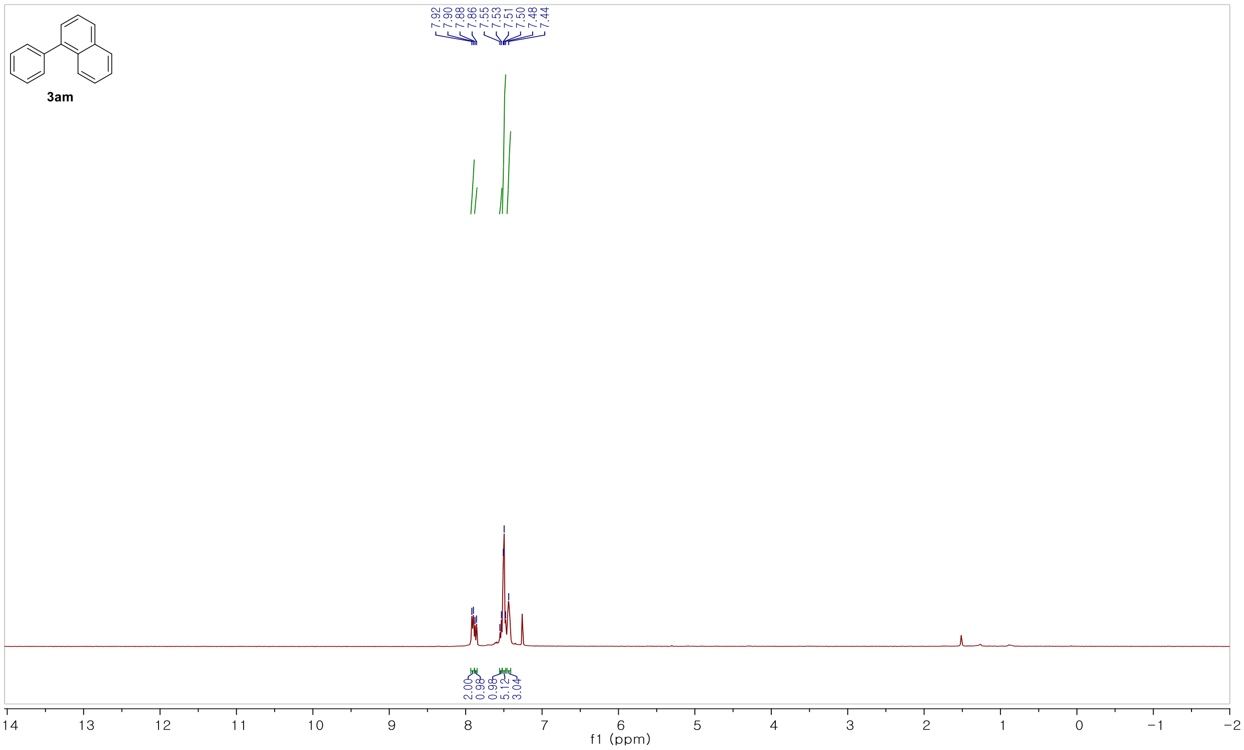
3am
7.92
7.90
7.88
7.86
7.55
7.53
7.51
7.50
7.48
7.44
2.00
0.98
0.98
5.12
3.04
14 13 12 11 10 9 8 7 6 5 4 3 2 1 0 −1 −2
f1 (ppm)

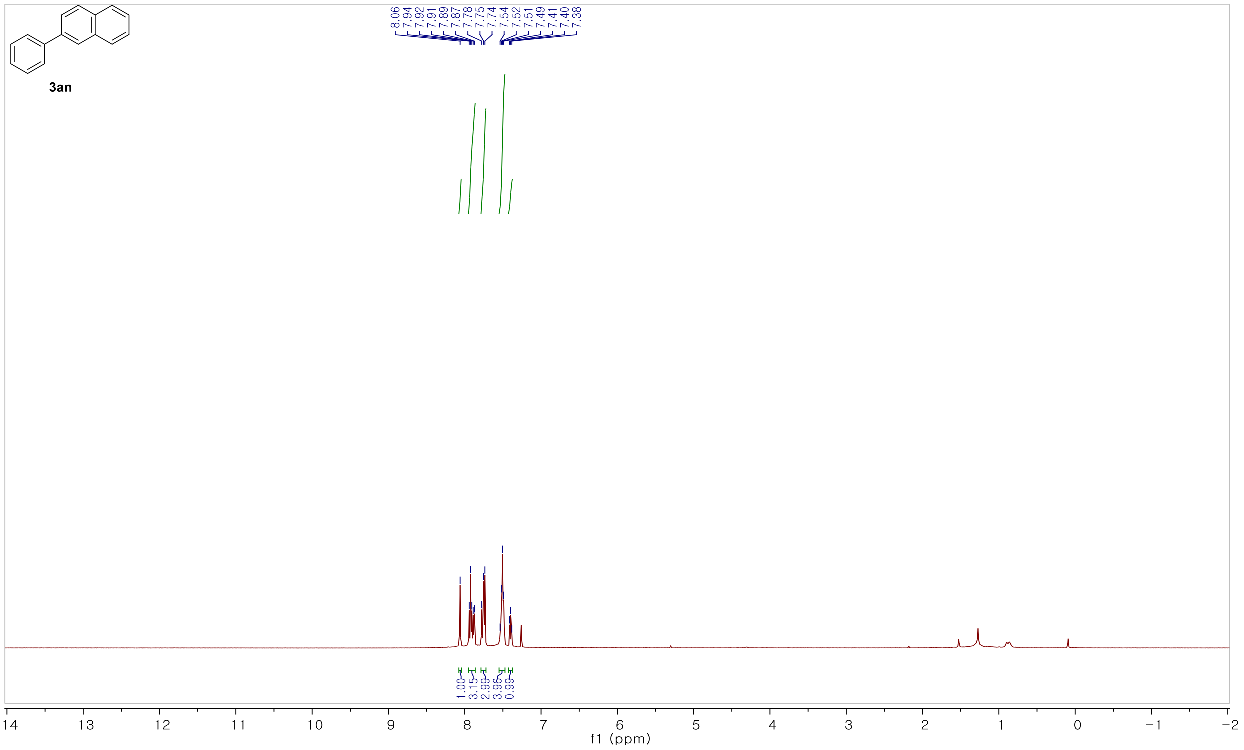
3an
8.06
7.94
7.92
7.91
7.89
7.87
7.78
7.75
7.74
7.54
7.52
7.51
7.49
7.41
7.40
7.38
1.00
3.15
2.99
3.96
0.99
14 13 12 11 10 9 8 7 6 5 4 3 2 1 0 −1 −2
f1 (ppm)

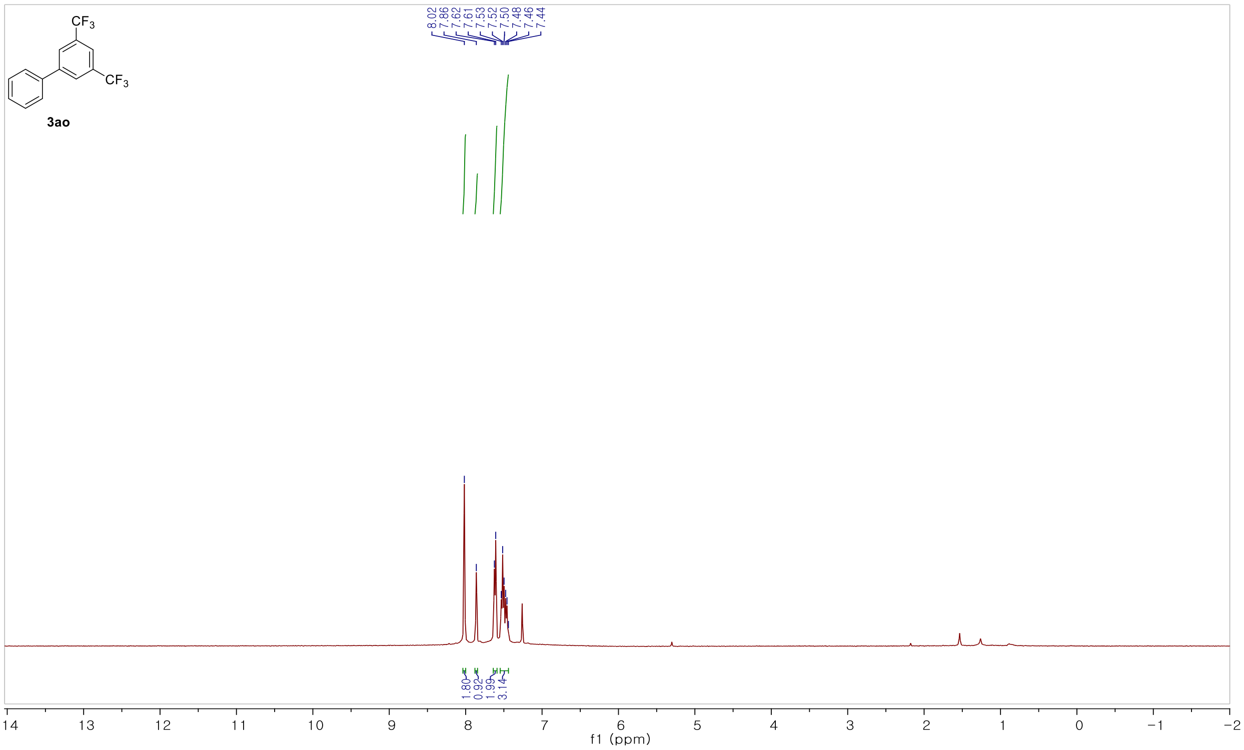
CF3
CF3
3ao
8.02
7.86
7.62
7.61
7.53
7.52
7.50
7.48
7.46
7.44
1.80
0.92
1.99
3.14
14 13 12 11 10 9 8 7 6 5 4 3 2 1 0 −1 −2
f1 (ppm)

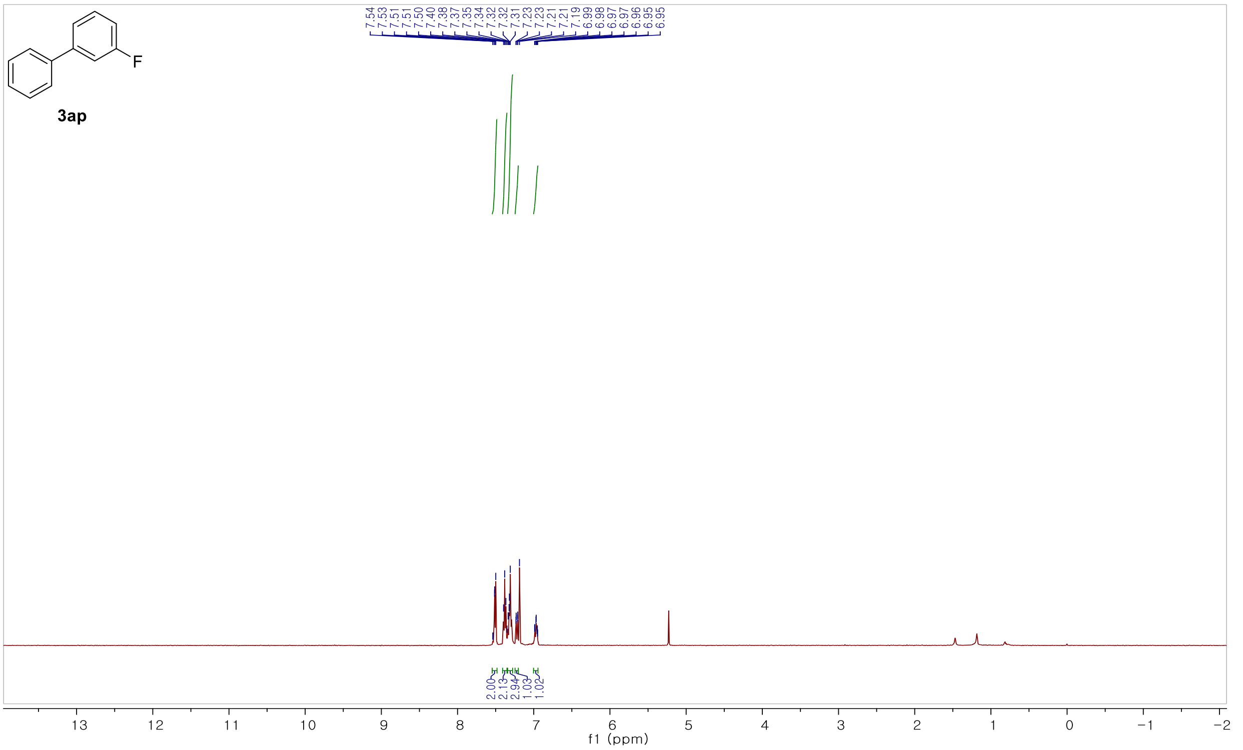
F
3ap
7.54
7.53
7.51
7.50
7.40
7.38
7.37
7.35
7.34
7.32
7.32
7.31
7.23
7.23
7.21
7.19
6.99
6.98
6.97
6.96
6.95
6.95
2.00
2.13
2.94
1.03
1.02
13 12 11 10 9 8 7 6 5 4 3 2 1 0 −1 −2
f1 (ppm)

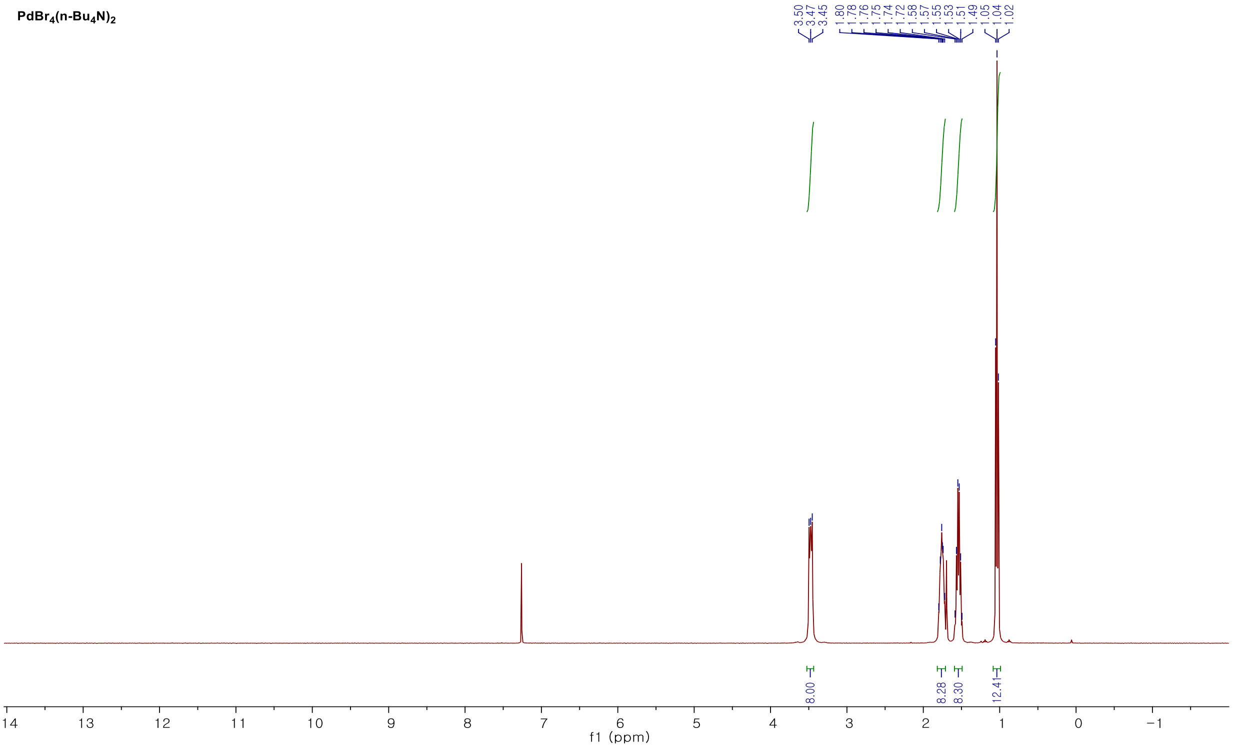
PdBr$_4$(n-Bu$_4$N)$_2$
3.50
3.47
3.45
1.80
1.78
1.76
1.75
1.74
1.72
1.58
1.57
1.55
1.53
1.51
1.49
1.05
1.04
1.02
8.00
8.28
8.30
12.41
14 13 12 11 10 9 8 7 6 5 4 3 2 1 0 −1
f1 (ppm)

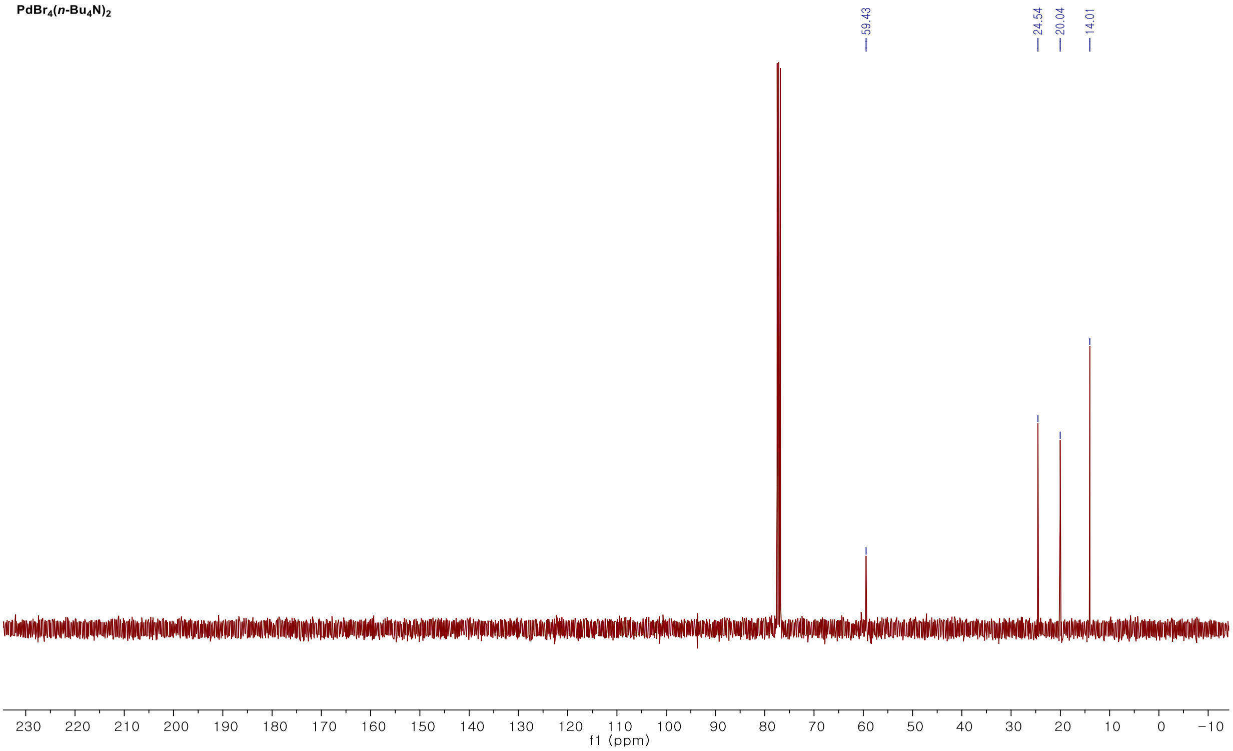
PdBr$_4$(*n*-Bu$_4$N)$_2$
59.43
24.54
20.04
14.01
230 220 210 200 190 180 170 160 150 140 130 120 110 100 90 80 70 60 50 40 30 20 10 0 −10
f1 (ppm)
