# Supplementary material for: Direct C(sp2)–H alkylation of unactivated arenes enabled by photoinduced Pd catalysis
Source: Nat Commun. 2020 Oct 19;11:5266. doi: 10.1038/s41467-020-19038-8 (PMC7572399; doi:10.1038/s41467-020-19038-8)
Supplement: Supplementary file 2 — Description of Additional Supplementary Files [file 41467_2020_19038_MOESM2_ESM.pdf]

Description of Additional Supplementary Files

File Name: Supplementary Data 1
Description: Cartesian coordinates of optimized geometry
