# Supplementary material for: Direct C(sp2)–H alkylation of unactivated arenes enabled by photoinduced Pd catalysis
Source: Nat Commun. 2020 Oct 19;11:5266. doi: 10.1038/s41467-020-19038-8 (PMC7572399; doi:10.1038/s41467-020-19038-8)
Supplement: Supplementary file 3 — Supplementary Data 1 [file 41467_2020_19038_MOESM3_ESM.zip › 257271_3_data_set_4901217_qgqz8q (1).docx]

**Supplementary** **Data for**

Direct C(sp^2^)–H Alkylation of Unactivated Arenes Enabled by Photoinduced Pd Catalysis

Daeun Kim^1,2^, Geun Seok Lee^1,2^, Dongwook Kim^1,3^, and Soon Hyeok Hong^1,^*

^1^Department of Chemistry, Korea Advanced Institute of Science and Technology (KAIST), Daejeon 34141, Republic of Korea

^2^Department of Chemistry, College of Natural Sciences, Seoul National University, Seoul 08826, Republic of Korea

^3^Center for Catalytic Hydrocarbon Functionalizations, Institute for Basic Science (IBS), Daejeon 34141, Republic of Korea

E-mail: Soon Hyeok Hong (soonhyeok.hong@kaist.ac.kr)

**Cartesian Coordinates of Optimized Geometry**

===============================

PPh_3_

===============================

P -0.000415000 0.000682000 -1.264402000

C -1.623068000 0.342459000 -0.439044000

C -1.771004000 1.116756000 0.721693000

C -2.769330000 -0.219200000 -1.024715000

C -3.033195000 1.318319000 1.283368000

H -0.898323000 1.562831000 1.187657000

C -4.028440000 -0.027048000 -0.457047000

H -2.673461000 -0.810601000 -1.931936000

C -4.163593000 0.745379000 0.698497000

H -3.131852000 1.922217000 2.181201000

H -4.904002000 -0.472367000 -0.920890000

H -5.144516000 0.903342000 1.137456000

C 0.514592000 -1.575946000 -0.440211000

C -0.090390000 -2.098899000 0.712625000

C 1.582813000 -2.280637000 -1.019014000

C 0.366401000 -3.293043000 1.273429000

H -0.919540000 -1.572083000 1.173687000

C 2.046135000 -3.467392000 -0.452017000

H 2.053950000 -1.896028000 -1.920214000

C 1.436585000 -3.978064000 0.695943000

H -0.113690000 -3.686370000 2.165229000

H 2.876243000 -3.997274000 -0.910503000

H 1.790480000 -4.906672000 1.134432000

C 1.107374000 1.234071000 -0.438525000

C 1.189362000 2.509673000 -1.020626000

C 1.857922000 0.972403000 0.717748000

C 1.985906000 3.503564000 -0.453117000

H 0.625173000 2.724343000 -1.924849000

C 2.664252000 1.964401000 1.279010000

H 1.812291000 -0.008074000 1.180853000

C 2.728013000 3.231921000 0.698275000

H 2.034230000 4.486096000 -0.913943000

H 3.241351000 1.745990000 2.173292000

H 3.355881000 4.002118000 1.136927000

===============================

**^1^A**

===============================

Pd -0.014682000 -0.050926000 0.010440000

P -1.452399000 1.643155000 1.042940000

P -1.072697000 -2.257877000 0.158243000

P 2.240675000 -0.059066000 1.053078000

P 0.285978000 0.587017000 -2.324817000

C 1.957801000 0.265220000 -3.050799000

C 2.656606000 1.179097000 -3.851411000

C 2.570832000 -0.938400000 -2.705215000

C 3.961181000 0.896091000 -4.278185000

H 2.201045000 2.124110000 -4.128652000

C 3.871874000 -1.233862000 -3.136313000

H 2.051419000 -1.643294000 -2.060882000

C 4.569187000 -0.307438000 -3.912603000

H 4.498425000 1.611884000 -4.894876000

H 4.336191000 -2.164104000 -2.823274000

H 5.584209000 -0.524551000 -4.233460000

C 0.050055000 2.381996000 -2.713944000

C -0.889402000 2.859425000 -3.647124000

C 0.812100000 3.307925000 -1.990334000

C -1.068479000 4.230341000 -3.832721000

H -1.480987000 2.156800000 -4.223183000

C 0.658844000 4.675437000 -2.192795000

H 1.528796000 2.962838000 -1.243440000

C -0.289656000 5.142364000 -3.114132000

H -1.806065000 4.589256000 -4.543974000

H 1.252933000 5.379837000 -1.618315000

H -0.432395000 6.207650000 -3.254761000

C -0.812573000 -0.207083000 -3.587303000

C -0.425060000 -0.441225000 -4.913360000

C -2.108424000 -0.584890000 -3.183794000

C -1.304486000 -1.045617000 -5.815490000

H 0.575619000 -0.156835000 -5.242555000

C -2.992198000 -1.176627000 -4.088163000

H -2.422521000 -0.440667000 -2.152945000

C -2.583637000 -1.412243000 -5.411026000

H -0.986460000 -1.223428000 -6.840266000

H -3.985254000 -1.471222000 -3.755449000

H -3.268651000 -1.884410000 -6.114016000

C -3.164365000 1.848972000 0.350512000

C -3.290804000 1.932788000 -1.048672000

C -4.319047000 1.924985000 1.141865000

C -4.548193000 2.092884000 -1.632878000

H -2.415362000 1.889859000 -1.676343000

C -5.579646000 2.067977000 0.543891000

H -4.240415000 1.868746000 2.225843000

C -5.694511000 2.151297000 -0.838497000

H -4.623273000 2.143930000 -2.719179000

H -6.460847000 2.133644000 1.175803000

H -6.677616000 2.259797000 -1.298458000

C -0.876577000 3.407784000 1.021899000

C 0.460276000 3.643632000 1.364077000

C -1.706293000 4.505330000 0.724724000

C 0.968308000 4.940235000 1.427321000

H 1.121123000 2.807735000 1.559424000

C -1.195355000 5.805410000 0.768720000

H -2.741076000 4.349662000 0.456058000

C 0.134141000 6.027181000 1.126659000

H 2.007419000 5.092103000 1.676346000

H -1.848597000 6.642632000 0.524470000

H 0.526556000 7.040754000 1.160785000

C -1.828103000 1.418654000 2.843690000

C -2.104401000 0.112519000 3.291097000

C -1.835838000 2.463666000 3.785646000

C -2.381349000 -0.143681000 4.629559000

H -2.063597000 -0.708588000 2.590716000

C -2.097771000 2.195876000 5.130159000

H -1.625720000 3.483985000 3.468509000

C -2.378193000 0.892873000 5.560399000

H -2.578814000 -1.168956000 4.942356000

H -2.093337000 3.012988000 5.843811000

H -2.578248000 0.700608000 6.608361000

C -0.285497000 -3.653868000 -0.784879000

C 0.047417000 -3.428840000 -2.128439000

C -0.002926000 -4.907948000 -0.218361000

C 0.663614000 -4.421198000 -2.891788000

H -0.185451000 -2.472790000 -2.587140000

C 0.623299000 -5.900027000 -0.982814000

H -0.252890000 -5.109150000 0.812680000

C 0.955055000 -5.662355000 -2.314815000

H 0.909164000 -4.220119000 -3.925252000

H 0.848598000 -6.863933000 -0.528479000

H 1.440808000 -6.434579000 -2.908327000

C -2.829762000 -2.417638000 -0.411222000

C -3.742436000 -1.460445000 0.062837000

C -3.283230000 -3.413851000 -1.296478000

C -5.081224000 -1.474363000 -0.352935000

H -3.410018000 -0.684967000 0.741660000

C -4.620723000 -3.437664000 -1.719683000

H -2.600779000 -4.174361000 -1.672983000

C -5.518907000 -2.463343000 -1.247959000

H -5.758841000 -0.705846000 0.016686000

H -4.951874000 -4.206741000 -2.408540000

H -6.558986000 -2.473528000 -1.583157000

C -1.195867000 -2.989624000 1.863869000

C -2.362225000 -3.554771000 2.382321000

C -0.065792000 -2.883226000 2.692287000

C -2.404564000 -3.991313000 3.708891000

H -3.251349000 -3.655374000 1.765003000

C -0.102097000 -3.329884000 4.010489000

H 0.832104000 -2.424348000 2.309859000

C -1.282295000 -3.882087000 4.530948000

H -3.325447000 -4.424569000 4.105837000

H 0.777710000 -3.225758000 4.627471000

H -1.315814000 -4.226128000 5.562252000

C 3.284000000 1.464922000 0.845892000

C 3.736233000 1.777019000 -0.444419000

C 3.595390000 2.339067000 1.887114000

C 4.454062000 2.938819000 -0.699850000

H 3.531409000 1.094087000 -1.271263000

C 4.312432000 3.513871000 1.640184000

H 3.283168000 2.120550000 2.905040000

C 4.737742000 3.822695000 0.352086000

H 4.777146000 3.158733000 -1.706102000

H 4.546776000 4.188518000 2.471039000

H 5.290977000 4.739460000 0.152971000

C 2.279152000 -0.276433000 2.898001000

C 3.124705000 -1.178885000 3.567822000

C 1.361675000 0.456381000 3.663267000

C 3.056833000 -1.326293000 4.955814000

H 3.845535000 -1.768795000 3.002788000

C 1.301673000 0.319856000 5.048986000

H 0.677420000 1.133641000 3.174027000

C 2.143873000 -0.585245000 5.704637000

H 3.721109000 -2.038552000 5.448490000

H 0.567937000 0.899738000 5.609894000

H 2.092310000 -0.704103000 6.783096000

C 3.455968000 -1.354712000 0.537971000

C 2.963925000 -2.610059000 0.163696000

C 4.847380000 -1.162249000 0.543127000

C 3.816079000 -3.640545000 -0.219981000

H 1.894042000 -2.766311000 0.138427000

C 5.715142000 -2.194242000 0.150028000

H 5.257669000 -0.210436000 0.835014000

C 5.197221000 -3.432345000 -0.229017000

H 3.401255000 -4.598414000 -0.516207000

H 6.787105000 -2.017814000 0.152956000

H 5.873675000 -4.225143000 -0.536592000

===============================

**^1^B**

===============================

Pd 0.119054000 0.100295000 -0.553114000

P -1.619796000 1.668713000 -0.230970000

P 2.399152000 0.564707000 -0.281049000

P -0.606866000 -2.129642000 -0.210315000

C 2.686883000 0.375880000 1.536085000

C 2.997064000 1.454797000 2.376099000

C 2.428367000 -0.880787000 2.115203000

C 3.029399000 1.284730000 3.764045000

H 3.202318000 2.431779000 1.950762000

C 2.471114000 -1.049009000 3.497082000

H 2.186164000 -1.726189000 1.481036000

C 2.762794000 0.036862000 4.328997000

H 3.268141000 2.131964000 4.401502000

H 2.264529000 -2.027782000 3.920823000

H 2.785007000 -0.090977000 5.407578000

C 3.128026000 2.230791000 -0.628174000

C 4.488020000 2.450204000 -0.896690000

C 2.256776000 3.329374000 -0.600911000

C 4.959717000 3.742305000 -1.133967000

H 5.177796000 1.612388000 -0.926292000

C 2.729104000 4.622012000 -0.830617000

H 1.202133000 3.163104000 -0.412440000

C 4.083163000 4.830392000 -1.100425000

H 6.013809000 3.899761000 -1.346313000

H 2.033575000 5.456392000 -0.815981000

H 4.454413000 5.833802000 -1.290123000

C 3.647954000 -0.568427000 -1.033871000

C 4.755910000 -1.087311000 -0.349538000

C 3.439517000 -0.945835000 -2.370181000

C 5.634019000 -1.966859000 -0.987429000

H 4.926150000 -0.816399000 0.687695000

C 4.322507000 -1.813799000 -3.011382000

H 2.562555000 -0.573835000 -2.894910000

C 5.422012000 -2.330203000 -2.318612000

H 6.483236000 -2.369101000 -0.441847000

H 4.146719000 -2.097860000 -4.045213000

H 6.105547000 -3.014676000 -2.813202000

C 0.665926000 -3.407151000 0.191641000

C 1.756018000 -3.524314000 -0.686745000

C 0.657362000 -4.176482000 1.364324000

C 2.813199000 -4.385403000 -0.401788000

H 1.791217000 -2.912110000 -1.581516000

C 1.723789000 -5.032226000 1.655701000

H -0.175168000 -4.097597000 2.056077000

C 2.803082000 -5.137533000 0.776803000

H 3.654228000 -4.442310000 -1.085911000

H 1.708888000 -5.615837000 2.572395000

H 3.634887000 -5.795968000 1.011110000

C -1.629183000 -2.953140000 -1.504664000

C -2.487949000 -2.137588000 -2.259500000

C -1.616733000 -4.335802000 -1.743232000

C -3.332420000 -2.696064000 -3.218367000

H -2.499144000 -1.066700000 -2.085965000

C -2.451124000 -4.890176000 -2.716138000

H -0.955787000 -4.977845000 -1.168753000

C -3.313591000 -4.073370000 -3.451355000

H -4.002436000 -2.050754000 -3.778886000

H -2.430566000 -5.961715000 -2.896073000

H -3.965195000 -4.508789000 -4.204024000

C -1.712469000 -2.179721000 1.272680000

C -2.900237000 -2.921860000 1.328748000

C -1.383753000 -1.341248000 2.351958000

C -3.754119000 -2.805712000 2.428788000

H -3.173136000 -3.569400000 0.501962000

C -2.235854000 -1.225593000 3.448974000

H -0.474727000 -0.749553000 2.314739000

C -3.430031000 -1.951733000 3.485301000

H -4.678877000 -3.376114000 2.453770000

H -1.979838000 -0.543516000 4.254282000

H -4.105826000 -1.848296000 4.329666000

C -1.796253000 2.105818000 1.558042000

C -0.639055000 2.053536000 2.351546000

C -3.013329000 2.450511000 2.166273000

C -0.693764000 2.329902000 3.717393000

H 0.305436000 1.767716000 1.901489000

C -3.070136000 2.723426000 3.534490000

H -3.923977000 2.482144000 1.576369000

C -1.912616000 2.661285000 4.314146000

H 0.215453000 2.266006000 4.308351000

H -4.021778000 2.979904000 3.992008000

H -1.962133000 2.866928000 5.379952000

C -1.517381000 3.318223000 -1.066505000

C -0.736285000 3.404266000 -2.228646000

C -2.160433000 4.472232000 -0.593776000

C -0.603967000 4.615827000 -2.909925000

H -0.207053000 2.519351000 -2.573669000

C -2.019290000 5.685652000 -1.267650000

H -2.760976000 4.426172000 0.309396000

C -1.242920000 5.759711000 -2.427786000

H 0.010744000 4.668036000 -3.803983000

H -2.514160000 6.574931000 -0.886781000

H -1.132284000 6.706638000 -2.948891000

C -3.316265000 1.106123000 -0.700479000

C -3.883853000 1.458214000 -1.935108000

C -3.969368000 0.143952000 0.090914000

C -5.068104000 0.857685000 -2.369878000

H -3.399445000 2.200798000 -2.562043000

C -5.153953000 -0.447344000 -0.342693000

H -3.546435000 -0.152893000 1.043621000

C -5.706768000 -0.097937000 -1.577451000

H -5.492609000 1.142793000 -3.328836000

H -5.633429000 -1.193864000 0.283804000

H -6.624901000 -0.567277000 -1.918996000

===============================

**^3^B**

===============================

Pd -0.003437000 0.459431000 -0.404623000

P 2.327858000 0.941306000 -0.136190000

P -2.265663000 1.099308000 -0.095992000

P -0.127228000 -1.900046000 -0.119127000

C -3.420923000 1.048664000 -1.515549000

C -4.487916000 1.957836000 -1.628417000

C -3.244088000 0.070600000 -2.504749000

C -5.365224000 1.878656000 -2.712707000

H -4.626286000 2.725906000 -0.873386000

C -4.134367000 -0.018278000 -3.573282000

H -2.409777000 -0.618134000 -2.433857000

C -5.193500000 0.890235000 -3.683219000

H -6.182693000 2.589610000 -2.796935000

H -3.997131000 -0.795368000 -4.319213000

H -5.878527000 0.829495000 -4.524413000

C -2.147996000 2.885133000 0.321848000

C -2.383144000 3.388744000 1.608808000

C -1.724512000 3.764572000 -0.693960000

C -2.193126000 4.746320000 1.879471000

H -2.717948000 2.723571000 2.398161000

C -1.542371000 5.120051000 -0.419761000

H -1.533515000 3.378395000 -1.691137000

C -1.775751000 5.614569000 0.865360000

H -2.376036000 5.125036000 2.881175000

H -1.214774000 5.789468000 -1.209911000

H -1.633207000 6.670690000 1.076632000

C -3.204649000 0.367121000 1.311398000

C -4.572399000 0.046867000 1.262287000

C -2.455653000 -0.031292000 2.433254000

C -5.165118000 -0.663372000 2.306636000

H -5.164917000 0.328929000 0.398571000

C -3.051719000 -0.731712000 3.479497000

H -1.388350000 0.167369000 2.454366000

C -4.410111000 -1.057960000 3.416158000

H -6.221474000 -0.913279000 2.252543000

H -2.444849000 -1.053950000 4.320422000

H -4.873638000 -1.626401000 4.217262000

C -1.594952000 -2.661655000 -0.907745000

C -2.849093000 -2.559249000 -0.275210000

C -1.528763000 -3.197488000 -2.204616000

C -4.008999000 -2.954964000 -0.942577000

H -2.922005000 -2.174532000 0.734112000

C -2.691089000 -3.601677000 -2.861526000

H -0.569835000 -3.290256000 -2.704292000

C -3.937244000 -3.468264000 -2.237296000

H -4.968489000 -2.846867000 -0.445310000

H -2.624450000 -4.015598000 -3.863716000

===============================

**2c**

===============================

C 0.608571000 0.639853000 -0.000011000

H 0.676626000 1.262347000 -0.892657000

H 0.676622000 1.262336000 0.892642000

C 1.606086000 -0.504692000 -0.000012000

H 1.436515000 -1.134050000 -0.879824000

H 1.436499000 -1.134070000 0.879780000

C 3.045557000 0.031463000 0.000011000

H 3.242661000 0.645817000 0.885348000

H 3.242686000 0.645754000 -0.885359000

H 3.762626000 -0.795068000 0.000057000

Br -1.315301000 -0.050080000 0.000002000

===============================

**^2^C**

===============================

Pd 0.017893000 0.381017000 -0.339079000

P -0.007619000 -2.046874000 -0.266972000

P -2.334877000 0.958038000 0.122236000

P 2.327087000 0.895913000 0.258247000

C -2.458074000 2.658648000 0.824896000

C -3.700933000 3.249037000 1.105380000

C -1.283039000 3.358269000 1.127044000

C -3.762367000 4.513202000 1.688726000

H -4.619802000 2.723796000 0.863196000

C -1.346606000 4.622707000 1.716939000

H -0.319572000 2.924917000 0.885558000

C -2.584369000 5.200175000 1.999106000

H -4.728422000 4.963602000 1.899501000

H -0.423798000 5.146963000 1.943732000

H -2.635334000 6.185820000 2.453737000

C -3.060727000 -0.077304000 1.465359000

C -3.606399000 -1.339985000 1.173105000

C -2.942428000 0.310091000 2.810459000

C -4.026056000 -2.186647000 2.197728000

H -3.708649000 -1.663596000 0.144168000

C -3.362783000 -0.542027000 3.834096000

H -2.525307000 1.281193000 3.057764000

C -3.906125000 -1.792662000 3.532105000

H -4.438114000 -3.159864000 1.947389000

H -3.269204000 -0.223230000 4.868650000

H -4.230840000 -2.455113000 4.329278000

C -3.635478000 0.935320000 -1.189388000

C -4.981097000 0.627193000 -0.932307000

C -3.249872000 1.255162000 -2.500849000

C -5.916430000 0.618314000 -1.968144000

H -5.302246000 0.376175000 0.072861000

C -4.190275000 1.252839000 -3.531900000

H -2.214662000 1.509134000 -2.709396000

C -5.523501000 0.927569000 -3.271713000

H -6.952344000 0.370153000 -1.753883000

H -3.875047000 1.504614000 -4.540621000

H -6.252407000 0.919233000 -4.077463000

C 2.457218000 2.515061000 1.131744000

C 2.286984000 3.697326000 0.388723000

C 2.632349000 2.602921000 2.521415000

C 2.303427000 4.936542000 1.027920000

H 2.121448000 3.642806000 -0.682173000

C 2.638294000 3.846505000 3.155923000

H 2.772332000 1.704171000 3.112506000

C 2.476854000 5.016957000 2.412601000

H 2.171967000 5.840311000 0.439800000

H 2.777565000 3.898193000 4.232244000

H 2.486659000 5.984106000 2.907569000

C 3.515484000 1.036509000 -1.129796000

C 3.260574000 0.295732000 -2.291047000

C 4.681826000 1.813359000 -1.049364000

C 4.169966000 0.312472000 -3.347637000

H 2.347230000 -0.281204000 -2.371096000

C 5.585406000 1.834779000 -2.112300000

H 4.878865000 2.405922000 -0.161020000

C 5.331929000 1.081024000 -3.261207000

H 3.961159000 -0.269923000 -4.239741000

H 6.483848000 2.441862000 -2.044382000

H 6.035327000 1.100690000 -4.089126000

C 3.142371000 -0.283629000 1.416626000

C 4.474923000 -0.703984000 1.289164000

C 2.344776000 -0.855602000 2.419059000

C 4.986290000 -1.687415000 2.137501000

H 5.104054000 -0.284377000 0.512110000

C 2.855778000 -1.836984000 3.268382000

H 1.306181000 -0.554592000 2.507089000

C 4.178203000 -2.260849000 3.123173000

H 6.016468000 -2.013016000 2.021616000

H 2.207678000 -2.291582000 4.010905000

H 4.575319000 -3.038846000 3.768970000

C 0.049089000 -3.021407000 1.292833000

C -0.564398000 -2.465667000 2.424678000

C 0.637486000 -4.292025000 1.393270000

C -0.585480000 -3.160453000 3.633678000

H -1.022474000 -1.485462000 2.360882000

C 0.627525000 -4.980535000 2.607178000

H 1.113633000 -4.739914000 0.527342000

C 0.016285000 -4.417044000 3.730085000

H -1.075113000 -2.714575000 4.493835000

H 1.094425000 -5.959175000 2.673763000

H 0.008095000 -4.955912000 4.673407000

C -1.508899000 -2.702118000 -1.113292000

C -2.074237000 -1.921665000 -2.134723000

C -2.159139000 -3.881492000 -0.718403000

C -3.274360000 -2.299698000 -2.737564000

H -1.594913000 -0.992816000 -2.429502000

C -3.359221000 -4.257956000 -1.322823000

H -1.747085000 -4.491152000 0.078584000

C -3.923182000 -3.465367000 -2.326630000

H -3.714793000 -1.659340000 -3.495141000

H -3.860346000 -5.166635000 -1.000654000

H -4.868112000 -3.751099000 -2.779814000

C 1.396785000 -2.686158000 -1.271374000

C 1.273848000 -2.834555000 -2.662307000

C 2.660098000 -2.861143000 -0.680361000

C 2.389497000 -3.144976000 -3.440991000

H 0.309229000 -2.703238000 -3.141260000

C 3.770965000 -3.170870000 -1.463128000

H 2.781407000 -2.751412000 0.390463000

C 3.642187000 -3.309912000 -2.846023000

H 2.277152000 -3.255700000 -4.515824000

H 4.739414000 -3.289227000 -0.986190000

H 4.510654000 -3.540710000 -3.455814000

Br 0.225740000 2.333418000 -2.288075000

===============================

**D**

===============================

C 1.321116000 -0.237856000 -0.000006000

C 0.070220000 0.576159000 0.000067000

C -1.214818000 -0.286754000 -0.000050000

H 0.048934000 1.232072000 0.880412000

H 0.048968000 1.232292000 -0.880110000

H -1.252315000 -0.931277000 0.884065000

H -2.110165000 0.345057000 0.000038000

H -1.252318000 -0.931042000 -0.884336000

H 1.728702000 -0.628548000 0.927320000

H 1.729085000 -0.627850000 -0.927456000

===============================

benzene

===============================

C 1.209256000 0.698164000 0.000000000

C 0.000000000 1.396329000 0.000000000

C 1.209256000 -0.698164000 0.000000000

H 0.000000000 2.482676000 0.000000000

H 2.150061000 -1.241338000 0.000000000

C -1.209256000 0.698164000 0.000000000

C 0.000000000 -1.396329000 0.000000000

H -2.150061000 1.241338000 0.000000000

H 0.000000000 -2.482676000 0.000000000

C -1.209256000 -0.698164000 0.000000000

H -2.150061000 -1.241338000 0.000000000

H 2.150061000 1.241338000 0.000000000

===============================

**D-TS**

===============================

C -1.653167000 -0.562477000 1.213017000

C -1.064275000 0.692706000 1.221045000

C -0.655261000 1.307069000 0.000436000

C -1.064292000 0.693553000 -1.220591000

C -1.653180000 -0.561634000 -1.213431000

C -1.937686000 -1.208632000 -0.000427000

H -1.917803000 -1.040473000 2.152474000

H -0.863643000 1.196713000 2.162159000

H -1.917827000 -1.038982000 -2.153214000

H -2.403812000 -2.189093000 -0.000763000

C 1.511543000 1.019963000 0.000201000

H 1.761999000 1.564290000 -0.910490000

H 1.762313000 1.564429000 0.910723000

C 1.779295000 -0.455160000 0.000242000

H 1.305239000 -0.910538000 0.877946000

H 1.304448000 -0.910710000 -0.876949000

C 3.283544000 -0.789378000 -0.000418000

H 3.781259000 -0.375375000 0.883355000

H 3.444936000 -1.873449000 -0.000367000

H 3.780416000 -0.375583000 -0.884763000

H -0.452974000 2.374500000 0.000805000

H -0.863677000 1.198214000 -2.161358000

===============================

**E**

===============================

C 1.828545000 1.225431000 -0.376619000

C 0.828326000 1.250853000 0.553714000

C 0.193179000 -0.000102000 1.101192000

C 0.828376000 -1.250948000 0.553523000

C 1.828595000 -1.225344000 -0.376805000

C 2.348785000 0.000091000 -0.867812000

H 2.240668000 2.160902000 -0.747467000

H 0.441282000 2.199799000 0.917998000

H 2.240756000 -2.160742000 -0.747794000

H 3.143136000 0.000163000 -1.606670000

C -1.353971000 -0.000112000 0.887834000

H -1.774327000 -0.879930000 1.394304000

H -1.774365000 0.879559000 1.394526000

C -1.781264000 0.000069000 -0.582928000

H -1.348766000 0.875968000 -1.081936000

H -1.348724000 -0.875679000 -1.082165000

C -3.302532000 0.000054000 -0.754021000

H -3.754225000 0.883654000 -0.288084000

H -3.586534000 0.000187000 -1.811514000

H -3.754180000 -0.883693000 -0.288318000

H 0.325674000 -0.000184000 2.201001000

H 0.441370000 -2.199964000 0.917663000

===============================

**[C + E]-TS**

===============================

Pd -0.178837000 -0.039160000 0.277571000

P -1.902796000 -0.023682000 -1.435962000

P 0.026800000 2.229961000 1.100747000

P -0.022067000 -2.237899000 1.198736000

C 0.823846000 2.398671000 2.763127000

C 1.120784000 3.647153000 3.332153000

C 1.094245000 1.234216000 3.492285000

C 1.673611000 3.724604000 4.609841000

H 0.926661000 4.558644000 2.774837000

C 1.639612000 1.312346000 4.775833000

H 0.891739000 0.268315000 3.044634000

C 1.929997000 2.556845000 5.335473000

H 1.904378000 4.695573000 5.040013000

H 1.840094000 0.396017000 5.322671000

H 2.359717000 2.620634000 6.331698000

C -1.648067000 2.942711000 1.422106000

C -2.397347000 3.517057000 0.379692000

C -2.261024000 2.774070000 2.674664000

C -3.714435000 3.922863000 0.589309000

H -1.956766000 3.643733000 -0.601655000

C -3.583542000 3.173628000 2.878699000

H -1.702239000 2.330035000 3.492575000

C -4.314541000 3.752514000 1.838687000

H -4.272964000 4.358151000 -0.234243000

H -4.039539000 3.036753000 3.855527000

H -5.343020000 4.063027000 1.999226000

C 0.867265000 3.567596000 0.123141000

C 0.428521000 4.900628000 0.077088000

C 2.013729000 3.218148000 -0.608770000

C 1.097452000 5.847395000 -0.701828000

H -0.448081000 5.206177000 0.637628000

C 2.691776000 4.168963000 -1.371482000

H 2.397857000 2.206702000 -0.560244000

C 2.230335000 5.485891000 -1.432499000

H 0.733106000 6.870715000 -0.730872000

H 3.593730000 3.874262000 -1.900079000

H 2.753131000 6.224819000 -2.033563000

C 0.522937000 -2.446779000 2.955992000

C 1.890961000 -2.368606000 3.272882000

C -0.404510000 -2.547566000 4.005214000

C 2.314546000 -2.403285000 4.600762000

H 2.620618000 -2.265654000 2.477885000

C 0.022819000 -2.570327000 5.333971000

H -1.465551000 -2.613918000 3.788760000

C 1.383248000 -2.501361000 5.638479000

H 3.376373000 -2.345924000 4.824285000

H -0.711285000 -2.649568000 6.131187000

H 1.715284000 -2.523916000 6.672694000

C 1.074973000 -3.391078000 0.269761000

C 1.358440000 -3.079016000 -1.067731000

C 1.604417000 -4.569032000 0.819553000

C 2.132912000 -3.936997000 -1.848066000

H 0.987069000 -2.150319000 -1.486635000

C 2.390001000 -5.421412000 0.041542000

H 1.408929000 -4.815246000 1.858852000

C 2.650617000 -5.110060000 -1.295299000

H 2.336705000 -3.680038000 -2.883598000

H 2.796248000 -6.329209000 0.479285000

H 3.259664000 -5.776427000 -1.900130000

C -1.648957000 -3.121858000 1.197793000

C -1.806007000 -4.477403000 0.869413000

C -2.792114000 -2.362915000 1.492629000

C -3.078432000 -5.051087000 0.829968000

H -0.939357000 -5.082767000 0.626292000

C -4.061868000 -2.938494000 1.463945000

H -2.684472000 -1.306732000 1.714705000

C -4.207852000 -4.285012000 1.126276000

H -3.186122000 -6.098876000 0.562589000

H -4.931582000 -2.322578000 1.668302000

H -5.196877000 -4.732549000 1.083705000

C -3.664046000 -0.167111000 -0.891987000

C -4.044150000 0.480432000 0.293997000

C -4.631456000 -0.887976000 -1.607843000

C -5.361610000 0.421637000 0.745959000

H -3.306183000 1.023074000 0.871728000

C -5.947650000 -0.958724000 -1.146354000

H -4.357259000 -1.400885000 -2.523544000

C -6.318306000 -0.301078000 0.028225000

H -5.631726000 0.941044000 1.660378000

H -6.683620000 -1.526874000 -1.708656000

H -7.343900000 -0.353986000 0.383265000

C -1.905763000 1.571501000 -2.366659000

C -0.662274000 2.183577000 -2.589425000

C -3.071692000 2.229539000 -2.784453000

C -0.581838000 3.436358000 -3.195595000

H 0.240210000 1.690826000 -2.242578000

C -2.992123000 3.486084000 -3.389262000

H -4.041611000 1.773931000 -2.613019000

C -1.750074000 4.094708000 -3.588861000

H 0.387400000 3.909537000 -3.319036000

H -3.902671000 3.994212000 -3.695398000

H -1.693372000 5.080920000 -4.040982000

C -1.830446000 -1.274740000 -2.792980000

C -1.858132000 -0.939913000 -4.155132000

C -1.711555000 -2.626742000 -2.431143000

C -1.748827000 -1.933767000 -5.130175000

H -1.952892000 0.098386000 -4.455491000

C -1.613994000 -3.618097000 -3.405424000

H -1.699201000 -2.905937000 -1.384374000

C -1.623950000 -3.274024000 -4.759716000

H -1.762088000 -1.657690000 -6.181037000

H -1.518154000 -4.656845000 -3.102592000

H -1.535787000 -4.044836000 -5.520224000

Br 2.820704000 -0.152051000 0.538896000

C 5.660276000 -1.103835000 -2.198841000

C 5.561602000 -1.070816000 -0.853096000

C 5.235518000 0.151877000 -0.154528000

H 5.713841000 -1.971532000 -0.265920000

C 5.409588000 0.097075000 -3.067136000

H 5.888131000 -2.038602000 -2.706338000

C 5.283239000 1.364008000 -2.268291000

C 4.142008000 -0.118788000 -3.961962000

H 6.255693000 0.209697000 -3.766793000

C 5.193336000 1.379204000 -0.921885000

H 5.226744000 2.290522000 -2.836622000

H 5.067499000 2.315248000 -0.385593000

H 5.460974000 0.217873000 0.902561000

H 4.007222000 0.771180000 -4.591962000

H 4.335454000 -0.958498000 -4.643458000

C 2.879477000 -0.386561000 -3.140087000

H 3.029664000 -1.297316000 -2.551729000

H 2.760199000 0.401808000 -2.390058000

C 1.611109000 -0.516004000 -3.977440000

H 1.679082000 -1.340315000 -4.697722000

H 0.746735000 -0.712886000 -3.339747000

H 1.399296000 0.400589000 -4.540900000

===============================

**F**

===============================

C 0.491221000 -1.329471000 1.250810000

C -0.843095000 -1.446215000 1.248691000

C -1.679096000 -1.429040000 -0.000036000

C -0.842976000 -1.446310000 -1.248674000

C 0.491345000 -1.329579000 -1.250654000

C 1.259720000 -1.175765000 0.000113000

H 1.052810000 -1.331285000 2.179942000

H -1.377997000 -1.549268000 2.190805000

H 1.053009000 -1.331504000 -2.179739000

Br 2.076653000 0.852446000 -0.000022000

C -2.673531000 -0.227405000 -0.000095000

H -3.325923000 -0.319890000 -0.878939000

H -3.325962000 -0.319871000 0.878722000

C -1.995668000 1.147589000 -0.000100000

H -1.337145000 1.229539000 0.872334000

H -1.337345000 1.229617000 -0.872679000

C -3.008579000 2.295464000 0.000065000

H -3.655870000 2.255000000 0.884215000

H -2.505420000 3.267485000 -0.000018000

H -3.656152000 2.254999000 -0.883880000

H -2.304529000 -2.337583000 -0.000058000

H -1.377781000 -1.549416000 -2.190838000

H 2.219410000 -1.689037000 0.000195000

===============================

KHCO_2_

===============================

O -0.907850000 -1.131410000 0.000049000

C -1.472176000 0.000004000 -0.000044000

O -0.907833000 1.131411000 0.000049000

H -2.588242000 0.000014000 -0.000262000

K 1.365619000 -0.000003000 -0.000014000

===============================

HCO_2_H

===============================

O 1.034662000 -0.441132000 0.000000000

C 0.000000000 0.421442000 0.000000000

O -1.164465000 0.110628000 0.000000000

H 0.384693000 1.452654000 0.000000000

H 0.653735000 -1.337273000 0.000000000

===============================

KBr

===============================

K 0.000000000 0.000000000 -1.899963000

Br 0.000000000 0.000000000 1.031408000

===============================

**3c**

===============================

C 2.849189000 -0.000240000 -0.311599000

C 2.168156000 1.205983000 -0.137751000

C 2.167795000 -1.206271000 -0.137507000

H 2.690660000 2.149750000 -0.266919000

H 2.690081000 -2.150183000 -0.266498000

C 0.816055000 1.203087000 0.206750000

C 0.815734000 -1.202921000 0.206979000

H 0.291493000 2.146016000 0.343026000

H 0.290855000 -2.145646000 0.343444000

C 0.119997000 0.000218000 0.384817000

H 3.902403000 -0.000443000 -0.576860000

C -1.357907000 0.000398000 0.703552000

H -1.608457000 -0.878630000 1.311174000

H -1.608404000 0.879907000 1.310500000

C -2.235044000 -0.000050000 -0.563313000

H -1.978660000 -0.876967000 -1.171222000

H -1.978836000 0.876559000 -1.171732000

C -3.732532000 -0.000168000 -0.246822000

H -4.012631000 0.883686000 0.337727000

H -4.012485000 -0.884023000 0.337793000

H -4.334674000 -0.000242000 -1.161070000

===============================

**2a-rad**

===============================

C -0.000002000 -1.410554000 0.271347000

C -1.266890000 -0.712460000 -0.244696000

C -1.287875000 0.777481000 0.159741000

C 0.000011000 1.463798000 -0.171702000

C 1.287874000 0.777484000 0.159740000

C 1.266884000 -0.712476000 -0.244699000

H -0.000021000 -2.465515000 -0.027946000

H -0.000003000 -1.395814000 1.371189000

H -2.164981000 -1.216204000 0.131925000

H -1.294320000 -0.785985000 -1.340022000

H -2.137606000 1.290639000 -0.305662000

H -1.465875000 0.829139000 1.251925000

H -0.000018000 2.526566000 -0.397966000

H 2.137628000 1.290617000 -0.305652000

H 1.465888000 0.829110000 1.251925000

H 2.164982000 -1.216199000 0.131926000

H 1.294317000 -0.785988000 -1.340025000

===============================

**2a-TS**

===============================

C 3.252954000 -0.000114000 -0.309892000

C 2.387942000 -1.268543000 -0.352611000

C 1.351156000 -1.275333000 0.784425000

C 0.545555000 0.000126000 0.848363000

C 1.351290000 1.275485000 0.784232000

C 2.388071000 1.268402000 -0.352791000

H 3.970944000 -0.000211000 -1.138591000

H 3.843720000 -0.000078000 0.617675000

H 3.017959000 -2.163427000 -0.289837000

H 1.870504000 -1.320767000 -1.319937000

H 0.688792000 -2.144534000 0.705309000

H 1.888791000 -1.392680000 1.742535000

H -0.244417000 0.000221000 1.598496000

H 0.689031000 2.144751000 0.704960000

H 1.888933000 1.392938000 1.742324000

H 3.018193000 2.163223000 -0.290156000

H 1.870675000 1.320516000 -1.320128000

C -0.826052000 0.000162000 -0.804006000

C -1.544569000 1.222012000 -0.601782000

C -1.544338000 -1.221852000 -0.601987000

H -1.091564000 2.161417000 -0.903597000

H -1.091156000 -2.161124000 -0.903950000

C -2.772265000 1.214044000 0.039019000

C -2.772030000 -1.214214000 0.038828000

H -3.280658000 2.152344000 0.244877000

H -3.280242000 -2.152641000 0.244551000

C -3.383530000 -0.000172000 0.394788000

H -4.347053000 -0.000303000 0.895046000

H -0.017553000 0.000332000 -1.529098000

===============================

**3a-rad**

===============================

C 3.485918000 -0.000125000 -0.070764000

C 2.682192000 -1.258963000 -0.416347000

C 1.309440000 -1.261585000 0.273653000

C 0.484597000 0.000097000 -0.047527000

C 1.309640000 1.261570000 0.273877000

C 2.682382000 1.258897000 -0.416148000

H 4.447248000 -0.000174000 -0.598172000

H 3.715287000 -0.000204000 1.004504000

H 3.240046000 -2.161431000 -0.138762000

H 2.536161000 -1.304292000 -1.504965000

H 0.760180000 -2.160789000 -0.021782000

H 1.452031000 -1.321371000 1.363538000

H 0.258957000 0.000170000 -1.123647000

H 0.760526000 2.160934000 -0.021341000

H 1.452259000 1.321084000 1.363774000

H 3.240354000 2.161235000 -0.138382000

H 2.536347000 1.304451000 -1.504753000

C -0.904259000 0.000138000 0.708510000

C -1.700165000 1.245860000 0.414493000

C -1.700138000 -1.245697000 0.414809000

C -2.897764000 1.221711000 -0.243880000

C -2.897730000 -1.221779000 -0.243583000

C -3.509709000 -0.000088000 -0.621404000

H -1.282082000 2.196543000 0.729482000

H -1.282046000 -2.196269000 0.730131000

H -3.406150000 2.157724000 -0.463048000

H -3.406064000 -2.157868000 -0.462546000

H -4.457245000 -0.000161000 -1.149775000

H -0.632235000 0.000199000 1.781620000

===============================

**2ad-rad**

===============================

C -0.570228000 -0.506337000 0.095524000

H -0.566092000 -1.056151000 1.056404000

H -0.433623000 -1.285456000 -0.670466000

C 0.579836000 0.446420000 0.047430000

H 0.407486000 1.475535000 0.357454000

C 1.984058000 -0.043253000 -0.069603000

H 2.073979000 -0.826892000 -0.834049000

H 2.347044000 -0.493056000 0.872110000

H 2.680866000 0.761632000 -0.325062000

C -1.936141000 0.167974000 -0.084580000

H -2.105224000 0.920703000 0.693612000

H -1.997149000 0.673953000 -1.053666000

H -2.752433000 -0.559083000 -0.028964000

===============================

**2ad-TS**

===============================

C 1.872250000 0.260858000 -1.310072000

C 1.065193000 1.203382000 -0.692567000

C 0.648052000 1.026389000 0.663650000

C 1.278115000 -0.015826000 1.412910000

C 2.086916000 -0.947587000 0.783166000

C 2.377740000 -0.831380000 -0.586701000

H 2.138771000 0.382246000 -2.356773000

H 0.702764000 2.061011000 -1.251462000

H 2.519821000 -1.762228000 1.357842000

H 3.016286000 -1.561648000 -1.073921000

C -1.406967000 0.384099000 0.521986000

H -1.571248000 0.252332000 1.593971000

C -1.388432000 -0.900718000 -0.260761000

H -1.044042000 -0.694658000 -1.282075000

H -0.653576000 -1.582261000 0.182220000

C -2.760919000 -1.599144000 -0.313416000

H -3.513724000 -0.970522000 -0.800679000

H -2.699742000 -2.538871000 -0.873106000

H -3.124043000 -1.832355000 0.693838000

H 0.285332000 1.897235000 1.204619000

H 1.073650000 -0.100387000 2.476606000

C -2.129648000 1.560157000 -0.076809000

H -1.800292000 1.739409000 -1.106889000

H -3.216108000 1.390897000 -0.111249000

H -1.967645000 2.478411000 0.498745000

===============================

**3ad-rad**

===============================

C 2.017816000 0.670470000 -1.106968000

C 0.912649000 1.212390000 -0.512176000

C 0.294688000 0.635509000 0.733680000

C 1.081394000 -0.530838000 1.269440000

C 2.184033000 -1.035958000 0.640819000

C 2.673960000 -0.461746000 -0.560434000

H 2.411407000 1.121052000 -2.015111000

H 0.441569000 2.090310000 -0.944921000

H 2.702374000 -1.890552000 1.069084000

H 3.549203000 -0.876415000 -1.049231000

C -1.223823000 0.257665000 0.547497000

H -1.536509000 -0.195846000 1.500636000

C -1.407624000 -0.796390000 -0.558328000

H -1.138084000 -0.347499000 -1.523118000

H -0.686105000 -1.604564000 -0.389559000

C -2.819074000 -1.386842000 -0.635662000

H -3.566283000 -0.633229000 -0.904653000

H -2.870331000 -2.180587000 -1.388100000

H -3.118331000 -1.821382000 0.325596000

H 0.284771000 1.425256000 1.510786000

H 0.720915000 -0.984086000 2.190190000

C -2.089042000 1.505044000 0.320761000

H -3.154407000 1.260513000 0.357552000

H -1.898989000 2.264922000 1.086889000

H -1.891063000 1.956283000 -0.657826000

===============================

**2aq-rad**

===============================

C -0.000029000 -0.000026000 -0.155651000

C 0.996512000 -1.103506000 0.015592000

C 0.457469000 1.414688000 0.015596000

H -0.246079000 2.126910000 -0.431618000

H 0.548020000 1.692597000 1.082117000

H 1.444259000 1.580516000 -0.432746000

H 1.192895000 -1.319960000 1.082121000

H 1.964667000 -0.850699000 -0.432720000

H 0.646285000 -2.041350000 -0.431628000

C -1.453977000 -0.311195000 0.015594000

H -1.719217000 -1.276156000 -0.432417000

H -1.739828000 -0.372624000 1.082096000

H -2.090853000 0.460996000 -0.431992000

===============================

**2aq-TS**

===============================

C -2.004828000 1.213105000 0.097488000

C -0.876226000 1.220773000 -0.704704000

C -0.194076000 0.000002000 -1.022573000

C -0.876218000 -1.220771000 -0.704699000

C -2.004823000 -1.213103000 0.097489000

C -2.569107000 0.000000000 0.527671000

H -2.476484000 2.152849000 0.373014000

H -0.470720000 2.162385000 -1.062083000

H -2.476480000 -2.152847000 0.373016000

H -3.457087000 0.000000000 1.152051000

H 0.460253000 -0.000005000 -1.892432000

H -0.470715000 -2.162383000 -1.062078000

C 2.260565000 -1.267529000 -0.213563000

C 1.552714000 0.000004000 0.200707000

C 2.260586000 1.267525000 -0.213560000

C 1.026967000 0.000001000 1.612230000

H 3.225788000 -1.355191000 0.308929000

H 2.474745000 -1.282910000 -1.288810000

H 1.674333000 -2.157284000 0.035123000

H 1.674355000 2.157288000 0.035100000

H 3.225795000 1.355178000 0.308956000

H 2.474792000 1.282884000 -1.288802000

H 0.414858000 -0.885427000 1.807405000

H 1.858370000 -0.000010000 2.334270000

H 0.414871000 0.885433000 1.807423000

===============================

**3aq-rad**

===============================

C 0.027825000 -0.000009000 -0.745952000

C -0.787513000 -1.245629000 -0.515603000

C -0.787556000 1.245608000 -0.515617000

H -0.337763000 -2.196856000 -0.782061000

H -0.337767000 2.196787000 -0.782182000

C -2.043698000 -1.221867000 0.023003000

C -2.043726000 1.221860000 0.022975000

H -2.569510000 -2.158062000 0.195216000

H -2.569572000 2.158041000 0.195157000

C -2.690036000 -0.000023000 0.336986000

H -3.684519000 -0.000031000 0.770542000

H 0.369851000 0.000060000 -1.798651000

C 1.388738000 -0.000028000 0.088565000

C 2.214855000 -1.247979000 -0.274510000

C 1.086935000 -0.000147000 1.595717000

H 0.508322000 0.883811000 1.880594000

H 0.508911000 -0.884494000 1.880577000

H 2.018406000 0.000150000 2.172678000

H 1.722007000 -2.171800000 0.040747000

H 3.189988000 -1.212697000 0.223334000

H 2.393856000 -1.307309000 -1.354621000

C 2.214646000 1.248185000 -0.274212000

H 1.721701000 2.171788000 0.041538000

H 3.189902000 1.212786000 0.223363000

H 2.393364000 1.307998000 -1.354347000

===============================

**1m**

===============================

C 1.195490000 -0.968896000 -0.000001000

C 1.209524000 0.424793000 0.000001000

C 0.000010000 -1.677491000 -0.000006000

H 0.000114000 -2.759935000 0.000000000

C -0.000008000 1.118325000 0.000000000

C -1.195466000 -0.968935000 -0.000001000

C -1.209521000 0.424767000 0.000002000

F 2.365211000 -1.622280000 0.000002000

F 2.365326000 1.097964000 0.000001000

F -0.000037000 2.453619000 -0.000003000

F -2.365315000 1.097929000 0.000001000

F -2.365218000 -1.622280000 0.000002000

===============================

**D-TS'**

===============================

C -1.055360000 1.259759000 0.007157000

C -0.103498000 1.153139000 -0.992124000

C 0.412877000 -0.098257000 -1.402024000

C -0.254222000 -1.243141000 -0.905435000

C -1.208830000 -1.158813000 0.093158000

C -1.627132000 0.099854000 0.543557000

C 2.410994000 -0.206433000 -0.451835000

H 2.633137000 -1.235076000 -0.730915000

H 2.925103000 0.543420000 -1.050657000

C 2.215199000 0.086216000 1.004563000

H 1.843996000 1.110861000 1.129783000

H 1.444105000 -0.579974000 1.413093000

C 3.510575000 -0.085018000 1.823144000

H 4.295148000 0.588830000 1.463849000

H 3.333989000 0.134686000 2.881638000

H 3.889641000 -1.109548000 1.748848000

H 0.910316000 -0.165632000 -2.362246000

F 0.130792000 -2.453337000 -1.345636000

F -1.774289000 -2.262258000 0.599753000

F -2.552501000 0.192299000 1.504843000

F -1.467120000 2.459284000 0.438048000

F 0.432113000 2.271635000 -1.509716000

===============================

**E'**

===============================

C -1.205251000 1.225752000 0.001427000

C -0.030761000 1.229923000 -0.689256000

C 0.725499000 -0.000050000 -1.087758000

C -0.030831000 -1.229968000 -0.689221000

C -1.205324000 -1.225711000 0.001455000

C -1.819581000 0.000043000 0.355299000

C 2.181576000 -0.000081000 -0.523755000

H 2.690711000 -0.881624000 -0.928394000

H 2.690798000 0.881345000 -0.928538000

C 2.263467000 0.000046000 1.005099000

H 1.736210000 0.878077000 1.398603000

H 1.736062000 -0.877825000 1.398761000

C 3.710831000 -0.000034000 1.504664000

H 4.251410000 0.884139000 1.148678000

H 3.753704000 0.000067000 2.598121000

H 4.251248000 -0.884373000 1.148846000

H 0.823841000 -0.000074000 -2.189220000

F 0.546429000 -2.394970000 -1.033877000

F -1.814723000 -2.369199000 0.346503000

F -2.960550000 0.000085000 1.051022000

F -1.814581000 2.369284000 0.346451000

F 0.546565000 2.394883000 -1.033942000

===============================

**^1^G**

===============================

Pd -0.251353000 -1.298233000 -0.481990000

P 1.778565000 0.103371000 0.005784000

C 1.643285000 1.761799000 0.820529000

C 1.281916000 2.898904000 0.078305000

C 1.841394000 1.901297000 2.202773000

C 1.134364000 4.139565000 0.697754000

H 1.134159000 2.825159000 -0.991863000

C 1.694334000 3.143667000 2.819930000

H 2.116655000 1.039916000 2.800984000

C 1.343663000 4.267977000 2.071755000

H 0.860092000 5.004679000 0.100483000

H 1.852148000 3.228831000 3.891053000

H 1.232976000 5.234562000 2.555116000

C 2.946506000 -0.758079000 1.135424000

C 4.320765000 -0.485370000 1.151270000

C 2.416019000 -1.665470000 2.063602000

C 5.149465000 -1.104652000 2.087212000

H 4.749111000 0.200721000 0.428154000

C 3.244204000 -2.272096000 3.007922000

H 1.360813000 -1.914967000 2.027100000

C 4.612041000 -1.993254000 3.020937000

H 6.215196000 -0.893994000 2.085332000

H 2.823225000 -2.977134000 3.718785000

H 5.259035000 -2.474491000 3.748947000

C 2.731793000 0.491371000 -1.525615000

C 3.649881000 1.553399000 -1.598036000

C 2.516028000 -0.298997000 -2.666099000

C 4.334518000 1.818545000 -2.784118000

H 3.817291000 2.186874000 -0.733011000

C 3.201346000 -0.027896000 -3.851867000

H 1.828796000 -1.137223000 -2.617236000

C 4.109540000 1.030002000 -3.914925000

H 5.040633000 2.643213000 -2.824735000

H 3.024768000 -0.650013000 -4.724516000

H 4.640464000 1.239781000 -4.839306000

Br 1.326210000 -3.332034000 -0.878817000

P -1.801165000 0.369275000 -0.072408000

C -1.548467000 1.034478000 1.627248000

C -1.205159000 0.094779000 2.615205000

C -1.725183000 2.373455000 1.994682000

C -1.076761000 0.482335000 3.948497000

H -1.033246000 -0.940396000 2.332688000

C -1.586089000 2.759204000 3.328308000

H -1.938928000 3.125535000 1.245568000

C -1.273564000 1.816605000 4.308565000

H -0.813862000 -0.255959000 4.700356000

H -1.709041000 3.803997000 3.597294000

H -1.167139000 2.123480000 5.345016000

C -1.710084000 1.752414000 -1.279781000

C -0.769607000 1.673687000 -2.317193000

C -2.582217000 2.853473000 -1.233704000

C -0.662227000 2.699382000 -3.258217000

H -0.116363000 0.809677000 -2.383542000

C -2.468206000 3.881631000 -2.168687000

H -3.362873000 2.897313000 -0.480717000

C -1.501696000 3.811065000 -3.176569000

H 0.078803000 2.624258000 -4.048432000

H -3.140810000 4.732942000 -2.117644000

H -1.417406000 4.612773000 -3.904573000

C -3.599982000 -0.032151000 -0.085053000

C -4.285597000 -0.069477000 -1.310826000

C -4.276816000 -0.411674000 1.082747000

C -5.619392000 -0.471420000 -1.364347000

H -3.775657000 0.217109000 -2.225532000

C -5.611411000 -0.817017000 1.024911000

H -3.767755000 -0.388479000 2.040047000

C -6.285699000 -0.849130000 -0.196252000

H -6.135497000 -0.494333000 -2.319667000

H -6.122663000 -1.107223000 1.938137000

H -7.323401000 -1.166705000 -0.238506000

C -1.825090000 -2.623997000 -0.922724000

H -2.643377000 -2.082380000 -1.402393000

H -1.385375000 -3.319588000 -1.638492000

C -2.285978000 -3.340383000 0.339617000

H -1.451060000 -3.922341000 0.745926000

H -2.580133000 -2.617031000 1.111722000

C -3.486330000 -4.262894000 0.064812000

H -3.240627000 -5.006313000 -0.701406000

H -3.792260000 -4.799871000 0.970502000

H -4.345861000 -3.682640000 -0.290113000

===============================

**^1^H**

===============================

Pd 1.533209000 0.640776000 -0.269533000

P -0.600433000 -0.055766000 0.005986000

C -1.852785000 1.275347000 -0.185006000

C -2.126004000 2.139191000 0.888220000

C -2.432140000 1.538882000 -1.435223000

C -2.983791000 3.225803000 0.719708000

H -1.675352000 1.956301000 1.859070000

C -3.287342000 2.629502000 -1.600626000

H -2.223421000 0.888401000 -2.278486000

C -3.568565000 3.472796000 -0.524418000

H -3.193900000 3.879609000 1.561147000

H -3.734720000 2.817293000 -2.572332000

H -4.236062000 4.319485000 -0.654543000

C -1.117020000 -1.353779000 -1.188155000

C -2.465294000 -1.744347000 -1.265463000

C -0.165390000 -1.990114000 -1.995850000

C -2.854436000 -2.750494000 -2.146318000

H -3.211570000 -1.257745000 -0.645567000

C -0.562548000 -3.000806000 -2.875030000

H 0.882092000 -1.717837000 -1.918948000

C -1.902156000 -3.379814000 -2.954062000

H -3.898630000 -3.044290000 -2.201943000

H 0.182715000 -3.491752000 -3.493533000

H -2.206720000 -4.165473000 -3.639625000

C -0.929316000 -0.778508000 1.664686000

C 0.132234000 -1.339347000 2.390303000

C -2.233154000 -0.840950000 2.185895000

C -0.112311000 -1.952014000 3.621003000

H 1.140739000 -1.312215000 1.988569000

C -2.467846000 -1.450642000 3.417151000

H -3.063181000 -0.401229000 1.642421000

C -1.407399000 -2.007270000 4.136986000

H 0.716045000 -2.385066000 4.173640000

H -3.478162000 -1.489699000 3.814169000

H -1.592311000 -2.481399000 5.096679000

Br 3.047313000 -1.450287000 -0.076498000

C 1.026185000 2.606832000 -0.529854000

H 0.425468000 2.732373000 -1.432330000

H 0.506678000 3.006433000 0.343292000

C 2.487089000 2.976438000 -0.638525000

H 3.108237000 2.025832000 -0.651081000

H 2.715954000 3.414098000 -1.617477000

C 3.022210000 3.835163000 0.513124000

H 2.812467000 3.356525000 1.475006000

H 4.103573000 3.981288000 0.435524000

H 2.541694000 4.818694000 0.511243000

===============================

**H-TS**

===============================

Pd 1.641259000 0.655977000 0.207594000

P -0.617289000 0.058008000 -0.010365000

C -0.974182000 -1.191598000 -1.302491000

C -0.099482000 -1.311261000 -2.390762000

C -2.110139000 -2.013197000 -1.233670000

C -0.369607000 -2.226063000 -3.408625000

H 0.804942000 -0.713157000 -2.420801000

C -2.374671000 -2.928660000 -2.252264000

H -2.781288000 -1.942173000 -0.382973000

C -1.506388000 -3.033505000 -3.341805000

H 0.318018000 -2.319063000 -4.243859000

H -3.253556000 -3.564040000 -2.191202000

H -1.710261000 -3.751711000 -4.130920000

C -1.308620000 -0.670838000 1.530465000

C -2.577884000 -0.331340000 2.021566000

C -0.530886000 -1.620415000 2.214151000

C -3.063893000 -0.935598000 3.182972000

H -3.185228000 0.404077000 1.503875000

C -1.025897000 -2.222022000 3.370646000

H 0.452474000 -1.886518000 1.835991000

C -2.290034000 -1.880647000 3.858381000

H -4.046969000 -0.665697000 3.558402000

H -0.419060000 -2.956137000 3.892643000

H -2.669464000 -2.348189000 4.762618000

C -1.763276000 1.454137000 -0.382513000

C -1.632453000 2.636252000 0.364412000

C -2.765248000 1.375801000 -1.358772000

C -2.489707000 3.712459000 0.146550000

H -0.855828000 2.709617000 1.121065000

C -3.615164000 2.462002000 -1.586026000

H -2.883414000 0.471266000 -1.945255000

C -3.482425000 3.629135000 -0.834598000

H -2.380370000 4.617796000 0.736826000

H -4.381857000 2.391045000 -2.352241000

H -4.144975000 4.471265000 -1.012284000

Br 2.622571000 -1.728969000 0.013086000

C 3.634380000 1.480465000 0.472503000

H 3.960130000 1.083048000 1.428680000

H 4.192509000 1.149858000 -0.398765000

C 2.859726000 2.658090000 0.412458000

H 1.302032000 2.206264000 0.356607000

H 2.657311000 3.162140000 1.357428000

C 2.873441000 3.554943000 -0.811150000

H 2.994851000 2.967254000 -1.724283000

H 1.957143000 4.144148000 -0.898188000

H 3.718743000 4.248906000 -0.733793000

===============================

**^1^I**

===============================

Pd -0.852081000 -0.401258000 -0.415538000

Br -0.739264000 -2.955873000 -0.002563000

P 1.372866000 0.222313000 -0.018343000

C 2.648378000 -0.669129000 -0.993612000

C 4.013979000 -0.380426000 -0.833591000

C 2.252760000 -1.657046000 -1.904660000

C 4.967873000 -1.062591000 -1.585273000

H 4.329844000 0.374197000 -0.119586000

C 3.213670000 -2.339970000 -2.654663000

H 1.202198000 -1.909669000 -2.001105000

C 4.567442000 -2.043024000 -2.498927000

H 6.022063000 -0.834520000 -1.456231000

H 2.899537000 -3.110202000 -3.352772000

H 5.312587000 -2.577657000 -3.081325000

C 1.657141000 1.998940000 -0.408916000

C 1.130048000 2.977011000 0.452899000

C 2.262021000 2.406023000 -1.605854000

C 1.218007000 4.329500000 0.129147000

H 0.655028000 2.674623000 1.381876000

C 2.342675000 3.762626000 -1.931020000

H 2.673786000 1.665573000 -2.283824000

C 1.824211000 4.726982000 -1.066618000

H 0.813703000 5.073740000 0.809484000

H 2.816271000 4.063078000 -2.861337000

H 1.890770000 5.780864000 -1.320382000

C 1.929856000 0.056254000 1.727991000

C 1.405440000 -1.000172000 2.489349000

C 2.884312000 0.912032000 2.301992000

C 1.836646000 -1.195771000 3.802416000

H 0.671957000 -1.670871000 2.050803000

C 3.306488000 0.713543000 3.616619000

H 3.287678000 1.742474000 1.731254000

C 2.783948000 -0.340976000 4.368877000

H 1.426340000 -2.017599000 4.381960000

H 4.042305000 1.383462000 4.052621000

H 3.113051000 -0.493389000 5.392939000

C -3.236064000 0.915738000 1.285309000

C -2.336301000 1.761586000 0.748444000

C -1.830463000 1.575626000 -0.619091000

H -1.954047000 2.604543000 1.316841000

C -3.747620000 -0.299112000 0.550643000

H -3.626417000 1.088402000 2.285433000

C -3.119064000 -0.439160000 -0.825473000

C -5.301856000 -0.300574000 0.460952000

H -3.470508000 -1.192182000 1.129209000

C -2.481321000 0.631521000 -1.464767000

H -3.455264000 -1.284291000 -1.419223000

H -2.301696000 0.627478000 -2.535960000

H -1.227924000 2.365460000 -1.055643000

H -5.623821000 -1.251680000 0.017356000

H -5.699675000 -0.288142000 1.485037000

C -5.896244000 0.869027000 -0.331546000

H -5.535515000 1.813840000 0.094848000

H -5.521436000 0.838617000 -1.362183000

C -7.427660000 0.853475000 -0.340266000

H -7.829364000 0.919875000 0.677451000

H -7.833353000 1.692904000 -0.914352000

H -7.810918000 -0.071784000 -0.785751000

===============================

**I-TS**

===============================

Pd -0.807575000 -0.799118000 0.111921000

Br -0.121910000 -3.189298000 0.897048000

P 1.191087000 0.391693000 -0.024316000

C 2.214821000 0.505174000 1.486104000

C 3.255773000 1.446005000 1.597009000

C 1.976593000 -0.399207000 2.532483000

C 4.041721000 1.484581000 2.753062000

H 3.447904000 2.151880000 0.796879000

C 2.772066000 -0.357992000 3.686222000

H 1.199593000 -1.155286000 2.442638000

C 3.800717000 0.581470000 3.794577000

H 4.849338000 2.222210000 2.832769000

H 2.588454000 -1.071009000 4.488995000

H 4.417282000 0.610390000 4.684550000

C 2.354267000 -0.189600000 -1.328001000

C 2.342344000 -1.561400000 -1.667530000

C 3.282030000 0.676454000 -1.945806000

C 3.261504000 -2.047477000 -2.594909000

H 1.632515000 -2.225902000 -1.189101000

C 4.181598000 0.176669000 -2.875693000

H 3.279855000 1.739211000 -1.701463000

C 4.177522000 -1.181854000 -3.210777000

H 3.241970000 -3.108543000 -2.853974000

H 4.897622000 0.850154000 -3.353852000

H 4.878327000 -1.569915000 -3.934776000

C 0.806852000 2.128162000 -0.482921000

C 0.646917000 3.130648000 0.498575000

C 0.472056000 2.421877000 -1.833091000

C 0.180052000 4.400326000 0.145534000

H 0.898421000 2.918045000 1.537729000

C 0.006477000 3.692657000 -2.172240000

H 0.579912000 1.654865000 -2.604826000

C -0.147577000 4.685504000 -1.190099000

H 0.069631000 5.158464000 0.925897000

H -0.246059000 3.908011000 -3.221365000

H -0.508636000 5.666440000 -1.461434000

C -3.400840000 0.664377000 -1.576078000

C -3.522583000 -0.247194000 -2.583883000

C -3.323800000 -1.631435000 -2.352831000

H -3.773202000 0.100428000 -3.587531000

C -3.003476000 0.244796000 -0.233044000

H -3.533680000 1.729592000 -1.754031000

C -2.979914000 -1.205353000 0.016029000

C -3.412894000 1.155972000 0.930766000

H -1.679502000 0.542387000 -0.308987000

C -3.092851000 -2.107274000 -1.083020000

H -3.117317000 -1.569419000 1.029108000

H -2.994966000 -3.160639000 -0.881882000

H -3.408324000 -2.333851000 -3.183672000

H -2.884728000 0.837345000 1.837404000

H -3.072005000 2.177017000 0.717769000

C -4.924867000 1.144714000 1.180482000

H -5.443916000 1.448223000 0.261245000

H -5.239761000 0.116353000 1.384138000

C -5.335389000 2.055735000 2.341486000

H -5.043353000 3.093777000 2.145261000

H -6.414989000 2.031107000 2.502704000

H -4.846098000 1.750104000 3.275055000

===============================

**^1^J**

===============================

Pd 0.260892000 -0.802291000 -0.074210000

P 1.162403000 1.376562000 -0.074159000

C 1.588035000 2.109542000 1.565128000

C 1.640649000 1.270103000 2.686293000

C 1.842943000 3.481888000 1.729723000

C 1.953365000 1.785273000 3.946164000

H 1.420629000 0.213225000 2.571268000

C 2.153086000 3.995773000 2.988582000

H 1.785971000 4.147528000 0.873970000

C 2.209814000 3.148538000 4.099304000

H 1.986749000 1.120743000 4.804887000

H 2.345652000 5.058579000 3.104195000

H 2.446162000 3.552802000 5.079495000

C 2.738903000 1.420567000 -1.029625000

C 3.914949000 2.020734000 -0.557021000

C 2.757771000 0.749882000 -2.266338000

C 5.091276000 1.951760000 -1.308041000

H 3.922753000 2.530434000 0.400430000

C 3.931842000 0.698284000 -3.017142000

H 1.865142000 0.240785000 -2.625032000

C 5.102148000 1.294644000 -2.539054000

H 5.998795000 2.411100000 -0.926344000

H 3.931817000 0.175693000 -3.969211000

H 6.018582000 1.240991000 -3.119906000

C 0.154839000 2.720613000 -0.830261000

C -0.936689000 3.226343000 -0.103583000

C 0.411607000 3.228968000 -2.110466000

C -1.738972000 4.230078000 -0.637553000

H -1.162794000 2.834870000 0.883311000

C -0.400697000 4.230935000 -2.646029000

H 1.246497000 2.852912000 -2.690803000

C -1.473067000 4.735919000 -1.912144000

H -2.585324000 4.599876000 -0.067883000

H -0.188951000 4.616238000 -3.639370000

H -2.105865000 5.511972000 -2.332633000

Br -0.112208000 -1.762560000 -2.725435000

P -2.120737000 -0.535518000 0.220604000

C -2.999877000 0.752359000 -0.752183000

C -4.083608000 1.480598000 -0.239402000

C -2.580078000 0.974211000 -2.073752000

C -4.745535000 2.412101000 -1.041230000

H -4.412771000 1.324695000 0.782818000

C -3.248445000 1.903759000 -2.868816000

H -1.741668000 0.410666000 -2.473255000

C -4.330776000 2.623073000 -2.357218000

H -5.584304000 2.971567000 -0.635905000

H -2.913220000 2.072297000 -3.887653000

H -4.844876000 3.350398000 -2.979598000

C -3.043724000 -2.077210000 -0.184175000

C -2.518957000 -3.297815000 0.264460000

C -4.246694000 -2.073635000 -0.901809000

C -3.190458000 -4.491962000 0.014251000

H -1.575363000 -3.306192000 0.799656000

C -4.914053000 -3.273346000 -1.159720000

H -4.659288000 -1.138851000 -1.265734000

C -4.390673000 -4.482514000 -0.701505000

H -2.771378000 -5.431166000 0.364352000

H -5.842168000 -3.260026000 -1.724157000

H -4.909431000 -5.414317000 -0.907955000

C -2.639351000 -0.201741000 1.960057000

C -1.809470000 0.599696000 2.757977000

C -3.827001000 -0.706872000 2.512049000

C -2.160901000 0.905711000 4.072392000

H -0.875293000 0.971419000 2.355469000

C -4.176618000 -0.404055000 3.829557000

H -4.475025000 -1.338143000 1.912185000

C -3.347377000 0.403652000 4.611697000

H -1.499857000 1.525483000 4.671995000

H -5.097738000 -0.802309000 4.245930000

H -3.621966000 0.633824000 5.637276000

C 2.965407000 -1.760708000 2.276686000

C 1.932763000 -2.569978000 2.596369000

C 1.110813000 -3.160690000 1.560113000

H 1.729982000 -2.828238000 3.632331000

C 3.274372000 -1.361917000 0.861080000

H 3.617115000 -1.366880000 3.053580000

C 2.197594000 -1.782805000 -0.133162000

C 4.676665000 -1.878571000 0.415715000

H 3.375854000 -0.270042000 0.847612000

C 1.332066000 -2.877867000 0.226386000

H 2.494867000 -1.729264000 -1.176340000

H 0.817278000 -3.430665000 -0.552209000

H 0.380122000 -3.913855000 1.840662000

H 4.907019000 -1.411445000 -0.550362000

H 5.424029000 -1.502921000 1.128962000

C 4.798046000 -3.401568000 0.301762000

H 4.513712000 -3.860365000 1.257389000

H 4.080110000 -3.768657000 -0.440910000

C 6.211507000 -3.843513000 -0.089101000

H 6.948309000 -3.520312000 0.655786000

H 6.281128000 -4.932680000 -0.176812000

H 6.505622000 -3.411888000 -1.052770000

===============================

**^3^J**

===============================

Pd -0.297989000 0.296718000 -0.361265000

P 1.730918000 1.379397000 0.167401000

C 2.172414000 1.083579000 1.933122000

C 1.756719000 -0.122151000 2.521791000

C 2.886995000 2.009122000 2.708929000

C 2.061674000 -0.403196000 3.853895000

H 1.191665000 -0.847209000 1.943019000

C 3.185674000 1.727747000 4.043273000

H 3.200784000 2.953042000 2.274140000

C 2.776026000 0.521853000 4.617998000

H 1.734299000 -1.343311000 4.289074000

H 3.736147000 2.453309000 4.635473000

H 3.008467000 0.308398000 5.657486000

C 3.235079000 0.917633000 -0.776349000

C 4.417456000 0.471645000 -0.171381000

C 3.140458000 0.943917000 -2.179057000

C 5.491082000 0.045243000 -0.958709000

H 4.499239000 0.444224000 0.910571000

C 4.220252000 0.531956000 -2.957873000

H 2.212545000 1.259315000 -2.649932000

C 5.394743000 0.074712000 -2.350549000

H 6.400597000 -0.308461000 -0.481376000

H 4.138848000 0.553149000 -4.040790000

H 6.229211000 -0.258970000 -2.960797000

C 1.638444000 3.205731000 0.019720000

C 0.386112000 3.819018000 0.176885000

C 2.768443000 3.998735000 -0.228147000

C 0.271948000 5.206743000 0.108595000

H -0.497889000 3.207359000 0.332162000

C 2.648414000 5.387480000 -0.302036000

H 3.737484000 3.530639000 -0.374072000

C 1.401659000 5.992727000 -0.130527000

H -0.702612000 5.671545000 0.227062000

H 3.527288000 5.994929000 -0.498830000

H 1.309363000 7.073319000 -0.193618000

Br -0.670062000 1.407964000 -2.764908000

P -2.515460000 -0.355472000 0.082538000

C -3.518522000 1.082375000 0.637603000

C -4.381138000 1.030495000 1.741950000

C -3.397462000 2.278510000 -0.091325000

C -5.114878000 2.159453000 2.112740000

H -4.472177000 0.115144000 2.318732000

C -4.137910000 3.399937000 0.281845000

H -2.724210000 2.325815000 -0.943956000

C -4.995016000 3.344366000 1.384388000

H -5.778174000 2.111956000 2.971937000

H -4.039876000 4.319581000 -0.288137000

H -5.564818000 4.221975000 1.676583000

C -3.560825000 -1.179920000 -1.174975000

C -2.959958000 -1.592298000 -2.372245000

C -4.933009000 -1.401812000 -0.974908000

C -3.717933000 -2.246651000 -3.345533000

H -1.914316000 -1.364480000 -2.549212000

C -5.685325000 -2.057030000 -1.948517000

H -5.410414000 -1.056807000 -0.061983000

C -5.076309000 -2.484727000 -3.132513000

H -3.247428000 -2.557506000 -4.273603000

H -6.745954000 -2.228453000 -1.788045000

H -5.665159000 -2.991539000 -3.891857000

C -2.500664000 -1.486674000 1.536389000

C -1.735012000 -1.086364000 2.646830000

C -3.108052000 -2.748932000 1.551670000

C -1.584913000 -1.927891000 3.746228000

H -1.240874000 -0.118313000 2.641270000

C -2.951378000 -3.594041000 2.654220000

H -3.697109000 -3.078598000 0.702458000

C -2.190796000 -3.188189000 3.751743000

H -0.986599000 -1.602042000 4.592323000

H -3.424215000 -4.572075000 2.651001000

H -2.067758000 -3.849327000 4.604702000

C 1.683563000 -3.302857000 1.120313000

C 0.370235000 -3.478652000 0.783085000

C -0.123627000 -3.078929000 -0.486768000

H -0.319295000 -3.917456000 1.499190000

C 2.690061000 -2.685046000 0.186784000

H 2.044462000 -3.615218000 2.098218000

C 2.075304000 -2.255342000 -1.116679000

C 3.930594000 -3.601555000 -0.041317000

H 3.102749000 -1.783122000 0.676761000

C 0.754300000 -2.455540000 -1.413691000

H 2.729918000 -1.770379000 -1.833681000

H 0.369981000 -2.125086000 -2.374634000

H -1.165927000 -3.232050000 -0.741653000

H 4.668662000 -3.034262000 -0.624534000

H 4.394930000 -3.808950000 0.933031000

C 3.608193000 -4.920071000 -0.750648000

H 2.859011000 -5.468319000 -0.165541000

H 3.135192000 -4.699305000 -1.715539000

C 4.847868000 -5.792962000 -0.964149000

H 5.320735000 -6.050552000 -0.008942000

H 4.597582000 -6.729067000 -1.474495000

H 5.597580000 -5.272365000 -1.571683000

===============================

**3x-rad**

===============================

C 4.814662000 -1.603332000 0.211479000

C 3.487547000 -1.550532000 -0.110717000

C 2.827449000 -0.304021000 -0.642010000

C 3.842829000 0.746809000 -1.011544000

C 5.163003000 0.649257000 -0.671540000

C 5.676023000 -0.501492000 -0.021245000

H 5.226999000 -2.517473000 0.631935000

H 2.864632000 -2.427279000 0.035864000

H 5.841944000 1.459562000 -0.926103000

H 6.725320000 -0.558183000 0.248283000

H 2.242820000 -0.567768000 -1.543527000

H 3.490185000 1.620965000 -1.549580000

C -0.067078000 2.010734000 0.745135000

C 1.050602000 1.516553000 -0.183862000

C 1.755075000 0.261041000 0.369736000

C 0.708474000 -0.805955000 0.751100000

C -0.394549000 -0.230289000 1.650082000

N -1.019201000 0.936768000 1.026568000

H -0.609540000 2.843573000 0.298952000

H 0.356156000 2.345017000 1.701252000

H 1.770689000 2.327212000 -0.332876000

H 0.607704000 1.288747000 -1.163297000

H 2.298945000 0.546267000 1.282035000

H 1.184710000 -1.644990000 1.268254000

H 0.240593000 -1.205381000 -0.159795000

H -1.156935000 -0.975938000 1.867209000

H 0.037178000 0.096801000 2.605305000

C -2.129407000 0.844596000 0.219914000

O -2.503958000 1.736710000 -0.528744000

O -2.753347000 -0.348363000 0.387224000

C -3.973692000 -0.670826000 -0.362168000

C -5.093268000 0.306308000 0.006703000

H -4.854895000 1.314331000 -0.331459000

H -6.029756000 -0.016358000 -0.459410000

H -5.236506000 0.319638000 1.091588000

C -3.677764000 -0.678401000 -1.864816000

H -4.552610000 -1.047381000 -2.409464000

H -3.434450000 0.323396000 -2.217665000

H -2.835830000 -1.345127000 -2.076791000

C -4.297773000 -2.081451000 0.133336000

H -4.461269000 -2.079129000 1.214753000

H -5.202470000 -2.453803000 -0.355969000

H -3.473563000 -2.764853000 -0.090807000

===============================

**4c-*o*-rad**

===============================

C 4.583089000 1.878592000 -0.075695000

C 3.306023000 1.711507000 0.383651000

C 2.701172000 0.350362000 0.635472000

C 3.787349000 -0.679213000 0.670510000

C 5.055708000 -0.503236000 0.211309000

C 5.469926000 0.781827000 -0.221172000

H 4.941173000 2.879149000 -0.303425000

H 2.668821000 2.575014000 0.539929000

H 5.741557000 -1.343977000 0.227567000

H 6.475425000 0.929156000 -0.598686000

H 2.183318000 0.339304000 1.609827000

F 3.430775000 -1.880524000 1.186523000

C -0.196422000 -1.643578000 -1.194973000

C 0.934775000 -1.367385000 -0.194774000

C 1.591734000 0.005702000 -0.438502000

C 0.509379000 1.102216000 -0.495486000

C -0.605501000 0.746064000 -1.488838000

N -1.181384000 -0.562546000 -1.183558000

H -0.708774000 -2.575742000 -0.959661000

H 0.210140000 -1.720052000 -2.212335000

H 1.674157000 -2.168492000 -0.259046000

H 0.517784000 -1.398533000 0.820511000

H 2.100894000 -0.020546000 -1.412946000

H 0.945805000 2.062712000 -0.786779000

H 0.062504000 1.231347000 0.500197000

H -1.392065000 1.497912000 -1.483849000

H -0.191473000 0.698849000 -2.505252000

C -2.313217000 -0.728755000 -0.421857000

O -2.671618000 -1.802917000 0.040816000

O -2.978656000 0.445257000 -0.277250000

C -4.240835000 0.506868000 0.470149000

C -5.299642000 -0.363796000 -0.211870000

H -5.029269000 -1.417847000 -0.154912000

H -6.267560000 -0.214944000 0.277312000

H -5.399172000 -0.078246000 -1.263641000

C -4.006468000 0.103547000 1.928791000

H -4.919586000 0.274084000 2.507902000

H -3.729570000 -0.947781000 2.000101000

H -3.206819000 0.712828000 2.361843000

C -4.607647000 1.988718000 0.369561000

H -4.726195000 2.284084000 -0.676892000

H -5.547658000 2.180654000 0.894936000

H -3.825071000 2.608242000 0.817304000

**===============================**

**4i-rad**

===============================

C 4.538965000 1.545129000 -0.306403000

C 3.301668000 1.450973000 0.253150000

C 2.603339000 0.168958000 0.601318000

C 3.645303000 -0.908967000 0.674750000

C 4.886481000 -0.840650000 0.119800000

C 5.341441000 0.383173000 -0.432486000

H 4.910198000 2.521097000 -0.600106000

F 2.582691000 2.574442000 0.487333000

H 5.527080000 -1.715227000 0.156196000

H 6.320528000 0.443616000 -0.892096000

H 2.116871000 0.273744000 1.583959000

F 3.254740000 -2.038334000 1.310344000

C -0.380756000 -1.818816000 -1.075147000

C 0.764035000 -1.517004000 -0.097980000

C 1.460146000 -0.183577000 -0.435235000

C 0.412493000 0.942325000 -0.546857000

C -0.717370000 0.560364000 -1.513238000

N -1.331187000 -0.709995000 -1.129766000

H -0.919746000 -2.718190000 -0.779545000

H 0.019796000 -1.972423000 -2.086294000

H 1.476295000 -2.344719000 -0.115168000

H 0.352733000 -1.468499000 0.918775000

H 1.955693000 -0.289703000 -1.411215000

H 0.872676000 1.873498000 -0.884834000

H -0.021851000 1.138494000 0.442462000

H -1.480467000 1.335052000 -1.547894000

H -0.311613000 0.442391000 -2.527483000

C -2.474949000 -0.800993000 -0.373154000

O -2.859055000 -1.833529000 0.158934000

O -3.119379000 0.391508000 -0.321902000

C -4.388741000 0.530564000 0.403038000

C -5.455396000 -0.370154000 -0.225721000

H -5.203466000 -1.421490000 -0.089523000

H -6.425269000 -0.170426000 0.240882000

H -5.539721000 -0.160095000 -1.296447000

C -4.177827000 0.232975000 1.890325000

H -5.094067000 0.461268000 2.444005000

H -3.920761000 -0.814800000 2.043034000

H -3.372480000 0.859102000 2.287275000

C -4.727820000 2.006743000 0.187670000

H -4.829402000 2.224674000 -0.879334000

H -5.670330000 2.252527000 0.685484000

H -3.939857000 2.645004000 0.597913000

===============================

**4k-rad**

===============================

C 4.244315000 1.478896000 -0.137380000

C 2.996113000 1.391264000 0.401586000

C 2.270641000 0.111991000 0.708445000

C 3.295782000 -0.982868000 0.799125000

C 4.548906000 -0.933760000 0.266710000

C 5.002517000 0.295802000 -0.253294000

H 4.667663000 2.437235000 -0.412903000

F 2.297604000 2.521607000 0.652171000

H 5.202216000 -1.797392000 0.296291000

F 6.222326000 0.354675000 -0.820953000

H 1.759876000 0.209432000 1.679178000

F 2.882021000 -2.105769000 1.427012000

C -0.686384000 -1.811791000 -1.080946000

C 0.436196000 -1.541258000 -0.069117000

C 1.153029000 -0.209030000 -0.363561000

C 0.121729000 0.931170000 -0.479938000

C -0.986289000 0.579434000 -1.482361000

N -1.622358000 -0.690799000 -1.137275000

H -1.242896000 -2.710230000 -0.816669000

H -0.261811000 -1.950646000 -2.084316000

H 1.141228000 -2.375632000 -0.083731000

H -0.000692000 -1.507527000 0.937513000

H 1.672880000 -0.302072000 -1.328346000

H 0.601749000 1.863480000 -0.786703000

H -0.336723000 1.112387000 0.501351000

H -1.740179000 1.362749000 -1.522564000

H -0.555424000 0.475191000 -2.487650000

C -2.784994000 -0.782452000 -0.409215000

O -3.195749000 -1.821567000 0.088738000

O -3.412407000 0.417921000 -0.343568000

C -4.701921000 0.556827000 0.345675000

C -5.761083000 -0.308020000 -0.343021000

H -5.527099000 -1.366691000 -0.233902000

H -6.741603000 -0.110305000 0.101629000

H -5.811399000 -0.062398000 -1.408299000

C -4.543413000 0.212822000 1.829522000

H -5.472669000 0.442563000 2.360394000

H -4.309918000 -0.843367000 1.960275000

H -3.740282000 0.812556000 2.269342000

C -5.010875000 2.044020000 0.163865000

H -5.074147000 2.295282000 -0.898754000

H -5.965159000 2.290331000 0.638387000

H -4.227033000 2.656894000 0.618398000

===============================

**4l-rad**

===============================

C 3.934265000 1.729659000 -0.128713000

C 2.686896000 1.589053000 0.400605000

C 2.006751000 0.279204000 0.682337000

C 3.058637000 -0.787960000 0.768432000

C 4.306416000 -0.669968000 0.230019000

C 4.741465000 0.582654000 -0.263821000

H 4.325157000 2.705731000 -0.389813000

F 1.946935000 2.688671000 0.660429000

F 5.163457000 -1.703433000 0.233569000

F 5.952281000 0.668882000 -0.833876000

H 1.491787000 0.344301000 1.653297000

F 2.703625000 -1.939050000 1.371646000

C -0.864283000 -1.722747000 -1.156231000

C 0.247109000 -1.429585000 -0.138585000

C 0.903071000 -0.059397000 -0.399272000

C -0.176368000 1.037567000 -0.486348000

C -1.271276000 0.661701000 -1.495264000

N -1.849522000 -0.643649000 -1.180802000

H -1.378825000 -2.652034000 -0.914857000

H -0.436519000 -1.815632000 -2.163399000

H 0.988481000 -2.231238000 -0.175907000

H -0.188931000 -1.441964000 0.868859000

H 1.427287000 -0.105571000 -1.365083000

H 0.261587000 1.996923000 -0.771902000

H -0.639837000 1.175388000 0.499637000

H -2.059129000 1.411787000 -1.513835000

H -0.840025000 0.602010000 -2.503891000

C -2.995576000 -0.801166000 -0.436247000

O -3.351912000 -1.867671000 0.044844000

O -3.672420000 0.368998000 -0.333976000

C -4.951517000 0.438541000 0.385790000

C -5.987514000 -0.459314000 -0.295711000

H -5.709338000 -1.509099000 -0.206602000

H -6.966490000 -0.307491000 0.169864000

H -6.068018000 -0.201058000 -1.356095000

C -4.743485000 0.074997000 1.858594000

H -5.670380000 0.249971000 2.413898000

H -4.457697000 -0.971227000 1.963137000

H -3.959884000 0.703940000 2.292867000

C -5.328614000 1.913718000 0.238569000

H -5.428107000 2.180344000 -0.817509000

H -6.281073000 2.110304000 0.739163000

H -4.561270000 2.552164000 0.685988000

===============================

**4m-rad**

===============================

C 3.766763000 1.423612000 -0.021566000

C 2.505257000 1.333538000 0.488032000

C 1.785093000 0.034579000 0.704556000

C 2.792047000 -1.076345000 0.779307000

C 4.053889000 -0.989420000 0.269565000

C 4.553388000 0.257505000 -0.175075000

F 4.316802000 2.611288000 -0.314199000

F 1.821285000 2.459213000 0.767901000

F 4.871926000 -2.052040000 0.249078000

F 5.754717000 0.330845000 -0.754860000

H 1.256015000 0.087182000 1.668257000

F 2.379895000 -2.230007000 1.336347000

C -1.137420000 -1.789239000 -1.236319000

C -0.029493000 -1.575564000 -0.194683000

C 0.692665000 -0.230262000 -0.407892000

C -0.331854000 0.919654000 -0.489670000

C -1.424854000 0.616766000 -1.524242000

N -2.070402000 -0.664305000 -1.243077000

H -1.697182000 -2.699891000 -1.026693000

H -0.700802000 -1.874599000 -2.240004000

H 0.675297000 -2.409182000 -0.241566000

H -0.483523000 -1.594520000 0.804717000

H 1.236623000 -0.276091000 -1.362777000

H 0.157378000 1.862640000 -0.745577000

H -0.807286000 1.058336000 0.490426000

H -2.174513000 1.405059000 -1.541263000

H -0.980326000 0.553567000 -2.526523000

C -3.209783000 -0.775988000 -0.477913000

O -3.613326000 -1.832355000 -0.012606000

O -3.818321000 0.426309000 -0.335892000

C -5.074917000 0.550684000 0.416751000

C -6.175324000 -0.271278000 -0.259180000

H -5.954282000 -1.336721000 -0.201186000

H -7.132894000 -0.076974000 0.234242000

H -6.266764000 0.016852000 -1.310924000

C -4.852843000 0.140659000 1.875291000

H -5.753516000 0.357492000 2.458221000

H -4.628002000 -0.922873000 1.949811000

H -4.022609000 0.711862000 2.302705000

C -5.370158000 2.047872000 0.312559000

H -5.477577000 2.345609000 -0.734370000

H -6.298460000 2.285640000 0.839901000

H -4.557942000 2.630784000 0.756588000

===============================

**3x-cat**

===============================

C 4.739390000 -1.595488000 0.286393000

C 3.397729000 -1.558545000 -0.007903000

C 2.779160000 -0.395114000 -0.642931000

C 3.710501000 0.627678000 -1.119268000

C 5.051215000 0.582167000 -0.822023000

C 5.559456000 -0.517491000 -0.102348000

H 5.173340000 -2.446809000 0.799238000

H 2.760508000 -2.391186000 0.269789000

H 5.720079000 1.372095000 -1.145610000

H 6.617548000 -0.543234000 0.144875000

H 2.071076000 -0.689775000 -1.428770000

H 3.310331000 1.459399000 -1.689099000

C -0.075387000 2.092069000 0.744624000

C 1.027667000 1.532415000 -0.188709000

C 1.695039000 0.311500000 0.451126000

C 0.665434000 -0.728738000 0.908581000

C -0.423933000 -0.059784000 1.786300000

N -1.034733000 1.054881000 1.081743000

H -0.592594000 2.907476000 0.239969000

H 0.367658000 2.475003000 1.670481000

H 1.749370000 2.329062000 -0.387035000

H 0.551467000 1.259256000 -1.138014000

H 2.319511000 0.621718000 1.295385000

H 1.130613000 -1.531967000 1.486486000

H 0.167723000 -1.179229000 0.040992000

H -1.182150000 -0.795153000 2.048584000

H 0.022369000 0.316690000 2.713737000

C -2.069553000 0.852866000 0.165895000

O -2.328517000 1.656368000 -0.713785000

O -2.695946000 -0.307661000 0.405214000

C -3.892024000 -0.710305000 -0.381601000

C -5.000617000 0.325311000 -0.194665000

H -4.728929000 1.280470000 -0.643845000

H -5.916548000 -0.038554000 -0.669233000

H -5.203549000 0.474550000 0.869682000

C -3.497559000 -0.893357000 -1.847638000

H -4.346813000 -1.302760000 -2.402127000

H -3.209231000 0.056146000 -2.298379000

H -2.666170000 -1.601023000 -1.931896000

C -4.265164000 -2.043993000 0.262450000

H -4.494325000 -1.909520000 1.322978000

H -5.145954000 -2.461873000 -0.232108000

H -3.444632000 -2.761763000 0.170585000

===============================

**4c-*o*-cat**

===============================

C -4.529813000 1.823258000 0.090570000

C -3.234223000 1.720338000 -0.333777000

C -2.630548000 0.424916000 -0.682833000

C -3.607392000 -0.653672000 -0.803554000

C -4.905095000 -0.573152000 -0.343632000

C -5.354364000 0.669427000 0.109583000

H -4.939931000 2.778407000 0.398317000

H -2.595698000 2.595925000 -0.359260000

H -5.553960000 -1.440037000 -0.390956000

H -6.378515000 0.757444000 0.461406000

H -1.976644000 0.467163000 -1.559749000

F -3.184438000 -1.781527000 -1.334771000

C 0.182769000 -1.624707000 1.379387000

C -0.912097000 -1.349120000 0.313136000

C -1.526668000 0.029852000 0.549458000

C -0.454408000 1.121320000 0.627307000

C 0.628152000 0.731053000 1.670201000

N 1.184168000 -0.575014000 1.366468000

H 0.660602000 -2.578559000 1.158605000

H -0.264183000 -1.675115000 2.378322000

H -1.655219000 -2.146916000 0.367010000

H -0.438595000 -1.398438000 -0.673596000

H -2.129429000 0.025722000 1.464118000

H -0.875094000 2.088500000 0.914043000

H 0.039695000 1.238939000 -0.344648000

H 1.416632000 1.481176000 1.671864000

H 0.183980000 0.698171000 2.671653000

C 2.230003000 -0.726395000 0.450926000

O 2.457726000 -1.785189000 -0.107522000

O 2.901341000 0.423396000 0.303831000

C 4.116415000 0.498492000 -0.551955000

C 5.181264000 -0.456303000 -0.013126000

H 4.873726000 -1.495827000 -0.125949000

H 6.113266000 -0.303418000 -0.564974000

H 5.373228000 -0.251658000 1.044070000

C 3.734345000 0.200019000 -2.002029000

H 4.598662000 0.380004000 -2.647680000

H 3.417529000 -0.836032000 -2.120821000

H 2.926470000 0.864617000 -2.325604000

C 4.540338000 1.955715000 -0.379615000

H 4.762180000 2.171938000 0.668890000

H 5.438099000 2.152838000 -0.971291000

H 3.749370000 2.632092000 -0.716866000

===============================

**4i-cat**

===============================

C 4.403522000 1.565342000 -0.313106000

C 3.159126000 1.482901000 0.257825000

C 2.533376000 0.222704000 0.676386000

C 3.518223000 -0.860289000 0.787400000

C 4.769323000 -0.815349000 0.225878000

C 5.193917000 0.402697000 -0.332312000

H 4.774509000 2.514586000 -0.680577000

F 2.418216000 2.566551000 0.379483000

H 5.418920000 -1.681282000 0.269748000

H 6.189792000 0.458345000 -0.761881000

H 1.922989000 0.332013000 1.576499000

F 3.111968000 -1.952894000 1.401165000

C -0.367780000 -1.889789000 -1.179094000

C 0.735517000 -1.545208000 -0.139940000

C 1.399513000 -0.221233000 -0.516480000

C 0.369680000 0.893604000 -0.702622000

C -0.719961000 0.436301000 -1.715264000

N -1.326690000 -0.808315000 -1.284618000

H -0.881510000 -2.795943000 -0.859909000

H 0.080887000 -2.063041000 -2.163290000

H 1.446189000 -2.372412000 -0.106635000

H 0.257918000 -1.470998000 0.843281000

H 1.997772000 -0.340057000 -1.426876000

H 0.817911000 1.816176000 -1.075065000

H -0.124386000 1.119901000 0.249079000

H -1.477781000 1.212469000 -1.800696000

H -0.268917000 0.284989000 -2.702575000

C -2.387174000 -0.825035000 -0.370776000

O -2.645944000 -1.805154000 0.305136000

O -3.030209000 0.348243000 -0.372594000

C -4.245514000 0.560551000 0.461692000

C -5.331474000 -0.430906000 0.045606000

H -5.047943000 -1.454651000 0.289200000

H -6.259033000 -0.188282000 0.572236000

H -5.520384000 -0.357106000 -1.029224000

C -3.869659000 0.441144000 1.938735000

H -4.731635000 0.713577000 2.554328000

H -3.569871000 -0.576973000 2.186935000

H -3.051875000 1.128491000 2.178787000

C -4.634490000 1.992699000 0.101134000

H -4.848743000 2.076820000 -0.967685000

H -5.528789000 2.284771000 0.657667000

H -3.828509000 2.688378000 0.352443000

===============================

**4k-cat**

===============================

C 4.134668000 1.496427000 -0.153722000

C 2.877845000 1.416860000 0.375763000

C 2.210751000 0.155625000 0.746527000

C 3.182962000 -0.945588000 0.874281000

C 4.447341000 -0.920179000 0.356637000

C 4.890916000 0.309447000 -0.164022000

H 4.563576000 2.432844000 -0.488751000

F 2.156639000 2.510601000 0.503577000

H 5.107917000 -1.777383000 0.400970000

F 6.105749000 0.363834000 -0.651675000

H 1.601765000 0.265980000 1.648361000

F 2.744556000 -2.036828000 1.463806000

C -0.675352000 -1.871539000 -1.190971000

C 0.411715000 -1.566533000 -0.126787000

C 1.103480000 -0.240734000 -0.455835000

C 0.087146000 0.891225000 -0.638746000

C -0.988089000 0.472978000 -1.678801000

N -1.618896000 -0.774585000 -1.290082000

H -1.208399000 -2.777549000 -0.904842000

H -0.211464000 -2.028181000 -2.171201000

H 1.112029000 -2.403143000 -0.101067000

H -0.080381000 -1.509625000 0.850655000

H 1.708699000 -0.348742000 -1.363923000

H 0.554979000 1.816059000 -0.981083000

H -0.419706000 1.102659000 0.309951000

H -1.735314000 1.259675000 -1.760700000

H -0.521749000 0.339046000 -2.661807000

C -2.700604000 -0.801266000 -0.403725000

O -2.990922000 -1.795749000 0.237921000

O -3.327595000 0.381462000 -0.387458000

C -4.563925000 0.585044000 0.416391000

C -5.650730000 -0.375690000 -0.064721000

H -5.387883000 -1.410926000 0.152157000

H -6.590248000 -0.137842000 0.442504000

H -5.806474000 -0.263160000 -1.141498000

C -4.236357000 0.412004000 1.899752000

H -5.112253000 0.680150000 2.497359000

H -3.961182000 -0.618501000 2.123880000

H -3.414993000 1.076780000 2.186727000

C -4.921396000 2.034056000 0.092213000

H -5.099710000 2.157108000 -0.979456000

H -5.829077000 2.320872000 0.629538000

H -4.114447000 2.708982000 0.392311000

===============================

**4l-cat**

===============================

C 3.842801000 1.739676000 -0.146214000

C 2.587345000 1.607309000 0.382493000

C 1.964143000 0.319482000 0.723124000

C 2.955399000 -0.762274000 0.826656000

C 4.215595000 -0.671803000 0.294464000

C 4.643393000 0.587641000 -0.187515000

H 4.240817000 2.697362000 -0.459587000

F 1.830613000 2.674763000 0.528037000

F 5.058527000 -1.691933000 0.300554000

F 5.859167000 0.669224000 -0.660622000

H 1.346198000 0.386998000 1.622933000

F 2.565902000 -1.892793000 1.372003000

C -0.848435000 -1.748930000 -1.296464000

C 0.234381000 -1.442966000 -0.224876000

C 0.857783000 -0.074169000 -0.500162000

C -0.207203000 1.017183000 -0.621602000

C -1.274260000 0.596432000 -1.673446000

N -1.841455000 -0.694379000 -1.335651000

H -1.334720000 -2.691405000 -1.047100000

H -0.386193000 -1.837957000 -2.285652000

H 0.972166000 -2.246872000 -0.244141000

H -0.252259000 -1.455322000 0.756713000

H 1.465986000 -0.113056000 -1.411565000

H 0.213447000 1.977240000 -0.925475000

H -0.715832000 1.161299000 0.338571000

H -2.057222000 1.351230000 -1.710781000

H -0.811981000 0.529957000 -2.664925000

C -2.909312000 -0.810145000 -0.436945000

O -3.141979000 -1.844614000 0.162918000

O -3.588995000 0.339431000 -0.360539000

C -4.823289000 0.449635000 0.466476000

C -5.870173000 -0.539081000 -0.045230000

H -5.558401000 -1.569515000 0.124828000

H -6.813704000 -0.366336000 0.480482000

H -6.042996000 -0.388237000 -1.114669000

C -4.467729000 0.226997000 1.936469000

H -5.347151000 0.427644000 2.555010000

H -4.142913000 -0.798494000 2.112134000

H -3.674323000 0.916191000 2.243788000

C -5.251241000 1.893126000 0.210984000

H -5.451310000 2.054457000 -0.851700000

H -6.162943000 2.113862000 0.772175000

H -4.471770000 2.591049000 0.530478000

===============================

**4m-cat**

===============================

C 3.691801000 1.436692000 -0.024980000

C 2.422786000 1.348337000 0.491867000

C 1.763587000 0.067100000 0.765300000

C 2.704172000 -1.056764000 0.832796000

C 3.976276000 -0.994346000 0.318866000

C 4.468624000 0.260286000 -0.100561000

F 4.233002000 2.592835000 -0.370181000

F 1.716599000 2.446044000 0.655277000

F 4.775722000 -2.047163000 0.286656000

F 5.688545000 0.338980000 -0.555902000

H 1.128268000 0.117032000 1.653811000

F 2.259918000 -2.196253000 1.314720000

C -1.125203000 -1.802394000 -1.366439000

C -0.038956000 -1.583686000 -0.271812000

C 0.650829000 -0.241265000 -0.498874000

C -0.352473000 0.906754000 -0.600564000

C -1.425704000 0.568490000 -1.678848000

N -2.061322000 -0.698103000 -1.379461000

H -1.659468000 -2.726512000 -1.148335000

H -0.656857000 -1.884469000 -2.352893000

H 0.657610000 -2.422702000 -0.309368000

H -0.540682000 -1.603467000 0.701945000

H 1.278736000 -0.280129000 -1.396391000

H 0.120359000 1.852057000 -0.872142000

H -0.869807000 1.049658000 0.354932000

H -2.166752000 1.364932000 -1.703829000

H -0.953557000 0.503178000 -2.665262000

C -3.132276000 -0.780631000 -0.477412000

O -3.420576000 -1.819826000 0.088487000

O -3.739777000 0.403488000 -0.356799000

C -4.957865000 0.561251000 0.489643000

C -6.068826000 -0.340630000 -0.045859000

H -5.818876000 -1.394102000 0.079815000

H -6.993537000 -0.132123000 0.499917000

H -6.245285000 -0.136843000 -1.105836000

C -4.601426000 0.261773000 1.945530000

H -5.457807000 0.499835000 2.582752000

H -4.345900000 -0.789053000 2.081443000

H -3.759076000 0.883862000 2.265573000

C -5.295099000 2.037316000 0.291948000

H -5.492679000 2.250698000 -0.761905000

H -6.186940000 2.293221000 0.869806000

H -4.471225000 2.672682000 0.629950000

===============================

**HAT-TS**

===============================

C -0.623127000 1.361540000 -0.080178000

C -1.966301000 1.003928000 0.060211000

C 0.328671000 0.372956000 -0.195030000

H 1.849688000 0.741293000 -0.348885000

C -2.315539000 -0.347308000 0.081400000

C -0.001798000 -0.963546000 -0.175608000

C -1.339629000 -1.338574000 -0.034816000

F -0.293644000 2.663127000 -0.099454000

F -2.919516000 1.937358000 0.173817000

F -3.599447000 -0.695890000 0.215641000

F -1.695820000 -2.629122000 -0.010821000

F 0.935940000 -1.922442000 -0.287598000

C 3.023139000 0.871397000 -0.441567000

C 3.701705000 -0.183547000 0.417982000

H 3.218492000 1.899368000 -0.121262000

H 3.230916000 0.763840000 -1.510616000

C 5.231592000 -0.118660000 0.307318000

H 3.402024000 -0.049264000 1.464577000

H 3.346115000 -1.175496000 0.118985000

H 5.610660000 0.858416000 0.626367000

H 5.703140000 -0.882904000 0.933636000

H 5.559069000 -0.281637000 -0.725324000

===============================

propane

===============================

H 2.175301000 0.362159000 -0.000009000

C 1.274515000 -0.260442000 0.000000000

H 1.315176000 -0.907819000 -0.883673000

H 1.315183000 -0.907806000 0.883682000

C -0.000135000 0.589285000 0.000000000

H -0.000412000 1.249117000 -0.877241000

H -0.000414000 1.249119000 0.877240000

C -1.274382000 -0.260580000 0.000000000

H -1.314636000 -0.907919000 0.883735000

H -1.314639000 -0.907920000 -0.883735000

H -2.175548000 0.361491000 0.000002000

===============================

**1m-rad**

===============================

C -1.217669000 -1.011324000 -0.000004000

C -1.214697000 0.388359000 -0.000001000

C -0.000003000 -1.648834000 0.000000000

C 0.000010000 1.076717000 -0.000001000

C 1.217667000 -1.011326000 -0.000001000

C 1.214713000 0.388347000 -0.000002000

F -2.382295000 -1.668999000 0.000001000

F -2.365215000 1.068742000 0.000001000

F -0.000020000 2.412536000 0.000002000

F 2.365219000 1.068760000 -0.000001000

F 2.382297000 -1.668999000 0.000002000

===============================

**HCO_2_^–^**

===============================

O -1.141627000 -0.208630000 0.000000000

O 1.141739000 -0.208066000 0.000000000

C 0.000000000 0.311372000 0.000000000

H -0.000897000 1.465339000 0.000000000

===============================

**E-anion**

===============================

C 2.055304000 1.299054000 -0.017037000

C 0.686244000 1.162400000 0.269151000

C 0.112752000 -0.184982000 0.328227000

C 0.959282000 -1.267476000 0.090425000

C 2.321835000 -1.129577000 -0.207737000

C 2.884433000 0.211882000 -0.265717000

H 2.484671000 2.305533000 -0.049469000

H 0.080632000 2.025537000 0.543617000

H 2.947394000 -2.006285000 -0.364864000

H 3.933110000 0.362248000 -0.520366000

C -1.350410000 -0.353804000 0.599108000

H -1.590118000 -1.413309000 0.786333000

H -1.652045000 0.196970000 1.518103000

C -2.273602000 0.157264000 -0.534010000

H -1.990854000 1.191787000 -0.766600000

H -2.064439000 -0.424818000 -1.441433000

C -3.765230000 0.082107000 -0.190581000

H -3.986405000 0.664864000 0.713276000

H -4.403681000 0.463850000 -0.997966000

H -4.070299000 -0.953794000 0.012220000

H 0.528383000 -2.273789000 0.136171000

===============================

**L**

===============================

H 2.548506000 1.895168000 0.881218000

C 1.555966000 1.846917000 0.448852000

C 0.832650000 3.023109000 0.309039000

C -0.425046000 3.001394000 -0.300577000

C -0.953823000 1.789458000 -0.742440000

C -0.258838000 0.588430000 -0.581327000

C 1.038170000 0.607316000 0.004878000

C 1.813916000 -0.622185000 0.040691000

C 1.348173000 -1.763342000 -0.676763000

C 0.109539000 -1.804430000 -1.232225000

C -0.899659000 -0.718931000 -1.001863000

H 1.256867000 3.960874000 0.654813000

H -0.985322000 3.921334000 -0.437070000

H -1.926285000 1.773611000 -1.225939000

H -1.478286000 -0.549704000 -1.921675000

H -0.225619000 -2.692837000 -1.761407000

H 2.027153000 -2.607045000 -0.747782000

C 3.087856000 -0.746459000 0.742346000

O 3.788216000 -1.756822000 0.710214000

H 3.410135000 0.121409000 1.349017000

C -1.912113000 -1.252358000 0.071674000

H -2.234504000 -2.252259000 -0.248014000

H -1.364703000 -1.390016000 1.012246000

C -3.151950000 -0.385202000 0.310524000

H -3.656466000 -0.196979000 -0.647415000

H -2.851295000 0.593084000 0.700399000

C -4.131256000 -1.043438000 1.287941000

H -3.655556000 -1.223706000 2.258421000

H -4.483680000 -2.009097000 0.908263000

H -5.008180000 -0.410949000 1.458711000

===============================

**L-anion**

===============================

H -2.888574000 1.862770000 0.314546000

C -1.817866000 1.811846000 0.146628000

C -1.098293000 3.005653000 0.101726000

C 0.282338000 2.974080000 -0.114478000

C 0.923452000 1.751634000 -0.280160000

C 0.223797000 0.513005000 -0.242091000

C -1.210672000 0.545914000 -0.016639000

C -1.953954000 -0.707629000 0.029157000

C -1.230352000 -1.921016000 -0.158823000

C 0.132406000 -1.926130000 -0.374601000

C 0.893550000 -0.741313000 -0.424678000

H -1.615160000 3.953890000 0.235740000

H 0.857214000 3.897852000 -0.152230000

H 1.995999000 1.745193000 -0.450261000

H 0.647612000 -2.877606000 -0.515316000

H -1.802267000 -2.843105000 -0.127011000

C -3.368842000 -0.799600000 0.249171000

O -4.037635000 -1.858164000 0.293165000

H -3.905653000 0.165369000 0.391873000

C 2.387919000 -0.817661000 -0.617618000

H 2.646955000 -1.789618000 -1.062383000

H 2.735871000 -0.060459000 -1.338851000

C 3.200827000 -0.655420000 0.684941000

H 2.907158000 -1.453206000 1.378966000

H 2.915990000 0.286675000 1.167851000

C 4.715310000 -0.688451000 0.457368000

H 5.029180000 0.123072000 -0.211118000

H 5.023588000 -1.632051000 -0.010852000

H 5.275459000 -0.582929000 1.394315000

===============================

PO_4_^3–^

===============================

O -0.281253000 0.791655000 1.361369000

P 0.000142000 -0.000037000 -0.000041000

O -0.581450000 -1.485822000 0.117303000

O -0.714315000 0.757687000 -1.214488000

O 1.576751000 -0.063452000 -0.264108000

===============================

HPO_4_^2–^

===============================

O -0.900469000 -1.305878000 -0.267634000

P -0.157684000 0.000341000 0.070795000

O -0.902585000 1.300976000 -0.284377000

O 0.585706000 0.010147000 1.437894000

O 1.277064000 -0.005904000 -0.989014000

H 1.887529000 0.000154000 -0.236880000

===============================

chloropropane

===============================

C -0.067843000 0.584774000 0.000014000

H 0.009152000 1.216346000 -0.888150000

H 0.009155000 1.216348000 0.888173000

C 0.983511000 -0.516496000 0.000012000

H 0.835528000 -1.152202000 -0.879582000

H 0.835547000 -1.152214000 0.879602000

C 2.399650000 0.071825000 -0.000012000

H 2.573573000 0.693688000 0.885053000

H 2.573587000 0.693576000 -0.885150000

H 3.149388000 -0.724750000 0.000058000

Cl -1.757520000 -0.095965000 -0.000005000

===============================

iodopropane

===============================

C 1.040052000 0.654294000 -0.000006000

H 1.123871000 1.277319000 -0.890949000

H 1.123881000 1.277322000 0.890933000

C 2.024679000 -0.504688000 -0.000007000

H 1.851810000 -1.133999000 -0.879341000

H 1.851804000 -1.134002000 0.879322000

C 3.469900000 0.016149000 0.000007000

H 3.672564000 0.628677000 0.885328000

H 3.672571000 0.628617000 -0.885352000

H 4.178845000 -0.817744000 0.000042000

I -1.069493000 -0.032466000 0.000001000

===============================

**^3^B-TS**

===============================

Pd 0.045594000 -0.319654000 -0.006681000

P 2.208492000 -1.358244000 -0.269854000

P -2.153499000 0.023623000 -0.892604000

P 0.587273000 1.864724000 0.762099000

C -3.642231000 -0.040742000 0.180286000

C -4.869763000 -0.528472000 -0.302294000

C -3.550097000 0.379509000 1.513249000

C -5.987215000 -0.575033000 0.532820000

H -4.948024000 -0.877989000 -1.327425000

C -4.670426000 0.338145000 2.343070000

H -2.600315000 0.717158000 1.905778000

C -5.890452000 -0.139341000 1.856790000

H -6.929681000 -0.957934000 0.151454000

H -4.582616000 0.677836000 3.370635000

H -6.759243000 -0.181315000 2.507812000

C -2.386562000 -1.417280000 -2.007206000

C -2.512075000 -1.311900000 -3.399338000

C -2.386758000 -2.693293000 -1.406957000

C -2.635166000 -2.462385000 -4.182774000

H -2.518423000 -0.334565000 -3.871300000

C -2.517421000 -3.835354000 -2.195123000

H -2.275818000 -2.781516000 -0.330564000

C -2.641943000 -3.725434000 -3.582601000

H -2.731929000 -2.370041000 -5.261051000

H -2.513934000 -4.814160000 -1.724156000

H -2.740413000 -4.618363000 -4.193556000

C -2.425145000 1.497294000 -1.963135000

C -3.612587000 2.247418000 -1.970515000

C -1.325993000 1.960926000 -2.706038000

C -3.689252000 3.435961000 -2.697821000

H -4.466942000 1.915440000 -1.390646000

C -1.406375000 3.144191000 -3.437302000

H -0.392149000 1.408021000 -2.672299000

C -2.588372000 3.890463000 -3.429620000

H -4.610809000 4.011830000 -2.688290000

H -0.534692000 3.498521000 -3.978953000

H -2.647344000 4.825089000 -3.979948000

C -0.814234000 2.677950000 1.619240000

C -1.818445000 3.304452000 0.857378000

C -0.985850000 2.558549000 3.007520000

C -2.973229000 3.785140000 1.474249000

H -1.701144000 3.415111000 -0.214142000

C -2.140869000 3.047587000 3.619111000

H -0.222835000 2.074988000 3.608547000

C -3.141305000 3.655699000 2.853556000

H -3.744916000 4.250417000 0.867922000

H -2.260543000 2.949110000 4.694298000

H -4.045198000 4.023511000 3.330294000

C 1.152118000 3.115970000 -0.465019000

C 1.803290000 2.659322000 -1.619209000

C 0.944201000 4.496621000 -0.301198000

C 2.247832000 3.557912000 -2.587691000

H 1.956173000 1.595869000 -1.760019000

C 1.369650000 5.393465000 -1.282995000

H 0.442261000 4.869843000 0.585426000

C 2.022658000 4.928163000 -2.426492000

H 2.770233000 3.181886000 -3.461771000

H 1.192576000 6.457376000 -1.151377000

H 2.354366000 5.629660000 -3.186983000

C 1.938464000 1.806379000 2.010267000

C 2.918286000 2.804981000 2.119053000

C 2.024959000 0.666340000 2.824303000

C 3.972171000 2.654195000 3.021232000

H 2.874979000 3.682091000 1.481994000

C 3.084242000 0.514905000 3.719633000

H 1.278506000 -0.117397000 2.727329000

C 4.062146000 1.506978000 3.815534000

H 4.732090000 3.427394000 3.094008000

H 3.158088000 -0.394270000 4.308273000

H 4.897229000 1.384122000 4.499485000

C 3.007758000 -2.131687000 1.206391000

C 2.166104000 -2.702390000 2.172262000

C 4.397407000 -2.188852000 1.391986000

C 2.703866000 -3.298844000 3.313252000

H 1.089330000 -2.657438000 2.040146000

C 4.932172000 -2.778808000 2.538789000

H 5.063880000 -1.762311000 0.650011000

C 4.087401000 -3.331671000 3.504185000

H 2.037444000 -3.730722000 4.054710000

H 6.009618000 -2.805489000 2.676706000

H 4.505789000 -3.788589000 4.396939000

C 2.066880000 -2.750127000 -1.469477000

C 1.040133000 -2.726705000 -2.425194000

C 2.998199000 -3.801190000 -1.491819000

C 0.953567000 -3.725178000 -3.396890000

H 0.304399000 -1.928500000 -2.409411000

C 2.904804000 -4.802298000 -2.459509000

H 3.792090000 -3.838329000 -0.752864000

C 1.886947000 -4.764215000 -3.415281000

H 0.148038000 -3.690677000 -4.122468000

H 3.629402000 -5.611988000 -2.466784000

H 1.818681000 -5.544769000 -4.168080000

C 3.543772000 -0.323045000 -0.999594000

C 3.763252000 -0.298194000 -2.390297000

C 4.221821000 0.617111000 -0.196544000

C 4.620381000 0.643898000 -2.956740000

H 3.257803000 -1.015060000 -3.029907000

C 5.074223000 1.557695000 -0.769273000

H 4.084152000 0.612490000 0.878010000

C 5.274157000 1.584221000 -2.152006000

H 4.781771000 0.641555000 -4.031590000

H 5.576376000 2.277177000 -0.128481000

H 5.932547000 2.324037000 -2.597428000

C -2.933216000 -2.806793000 3.228616000

H -2.474430000 -3.552762000 3.877770000

H -3.304738000 -1.950488000 3.790819000

C -3.911792000 -3.348722000 2.216571000

Cl -1.363493000 -1.988286000 2.186255000

H -3.444124000 -4.173813000 1.664877000

H -4.138099000 -2.567524000 1.484020000

C -5.221521000 -3.824242000 2.865205000

H -5.721767000 -2.995400000 3.378121000

H -5.043755000 -4.617604000 3.599854000

H -5.913282000 -4.213569000 2.109033000

===============================

**^2^C-Cl**

===============================

Pd 0.016438000 0.417335000 -0.468439000

P -0.005474000 -1.976059000 -0.155861000

P -2.334022000 1.039333000 -0.029164000

P 2.334761000 0.997331000 0.051866000

C -2.452797000 2.798400000 0.507583000

C -3.691422000 3.444276000 0.646055000

C -1.278576000 3.490687000 0.828736000

C -3.750188000 4.758203000 1.107603000

H -4.607661000 2.922944000 0.385931000

C -1.339423000 4.804955000 1.296800000

H -0.316722000 3.009630000 0.696046000

C -2.573972000 5.438997000 1.437471000

H -4.712760000 5.252116000 1.207859000

H -0.416917000 5.324361000 1.537261000

H -2.622854000 6.463550000 1.796187000

C -3.085019000 0.138047000 1.392131000

C -3.617255000 -1.151242000 1.212607000

C -2.985536000 0.649159000 2.697013000

C -4.036464000 -1.905775000 2.307018000

H -3.706743000 -1.568782000 0.217038000

C -3.405682000 -0.110809000 3.790765000

H -2.579687000 1.642830000 2.858460000

C -3.930802000 -1.390912000 3.600732000

H -4.436375000 -2.902207000 2.143181000

H -3.325568000 0.302712000 4.792464000

H -4.253140000 -1.982021000 4.453044000

C -3.601565000 0.902687000 -1.363589000

C -4.952284000 0.609598000 -1.118057000

C -3.176746000 1.108178000 -2.686631000

C -5.856738000 0.502438000 -2.175847000

H -5.300862000 0.448106000 -0.103436000

C -4.086937000 1.006599000 -3.739532000

H -2.135824000 1.351356000 -2.883945000

C -5.426180000 0.696635000 -3.489775000

H -6.897689000 0.267818000 -1.970868000

H -3.744218000 1.169100000 -4.757612000

H -6.130910000 0.611489000 -4.312449000

C 2.495218000 2.689473000 0.766972000

C 2.172205000 3.788868000 -0.049433000

C 2.861820000 2.914542000 2.102625000

C 2.228976000 5.083574000 0.464737000

H 1.851378000 3.621179000 -1.072772000

C 2.905432000 4.213624000 2.613293000

H 3.120347000 2.080573000 2.746388000

C 2.593234000 5.301692000 1.796889000

H 1.977291000 5.922659000 -0.177625000

H 3.192390000 4.372843000 3.649122000

H 2.633132000 6.311990000 2.194735000

C 3.475884000 1.015576000 -1.382367000

C 3.191096000 0.166232000 -2.459455000

C 4.633550000 1.807955000 -1.415641000

C 4.064402000 0.091039000 -3.543739000

H 2.283052000 -0.424700000 -2.451349000

C 5.500891000 1.736876000 -2.506330000

H 4.851618000 2.483238000 -0.593732000

C 5.219158000 0.874938000 -3.569453000

H 3.834210000 -0.575560000 -4.369306000

H 6.393590000 2.355803000 -2.527225000

H 5.894852000 0.822096000 -4.418747000

C 3.189399000 -0.069637000 1.286119000

C 4.514085000 -0.511066000 1.151344000

C 2.427410000 -0.531805000 2.370048000

C 5.052012000 -1.412465000 2.071621000

H 5.115508000 -0.172421000 0.314867000

C 2.964415000 -1.431708000 3.290130000

H 1.396008000 -0.210540000 2.469643000

C 4.277974000 -1.880936000 3.136762000

H 6.075596000 -1.756495000 1.950639000

H 2.343989000 -1.804190000 4.099272000

H 4.694779000 -2.596361000 3.839997000

C 0.059840000 -2.768200000 1.503641000

C -0.525402000 -2.075284000 2.573073000

C 0.630908000 -4.028492000 1.740557000

C -0.536134000 -2.625378000 3.854473000

H -0.969293000 -1.100884000 2.403639000

C 0.630807000 -4.572445000 3.025814000

H 1.086488000 -4.579760000 0.924526000

C 0.048202000 -3.872601000 4.085551000

H -1.004470000 -2.075648000 4.664963000

H 1.083660000 -5.544793000 3.198093000

H 0.048280000 -4.299286000 5.084685000

C -1.512030000 -2.718904000 -0.917808000

C -2.086564000 -2.052287000 -2.012000000

C -2.154544000 -3.852369000 -0.397548000

C -3.288080000 -2.496281000 -2.565239000

H -1.614056000 -1.156884000 -2.404014000

C -3.356038000 -4.295385000 -0.951938000

H -1.734965000 -4.372365000 0.456668000

C -3.929068000 -3.615234000 -2.030292000

H -3.734685000 -1.940508000 -3.383652000

H -3.851341000 -5.167097000 -0.533084000

H -4.875078000 -3.951022000 -2.445301000

C 1.388395000 -2.728558000 -1.093863000

C 1.255256000 -3.018463000 -2.461716000

C 2.654255000 -2.851872000 -0.496182000

C 2.363171000 -3.414684000 -3.211578000

H 0.287885000 -2.931499000 -2.945337000

C 3.757746000 -3.248537000 -1.250049000

H 2.783712000 -2.635756000 0.557161000

C 3.619099000 -3.526441000 -2.610775000

H 2.242479000 -3.634661000 -4.268579000

H 4.728273000 -3.325548000 -0.768911000

H 4.481694000 -3.825154000 -3.199058000

Cl 0.155891000 2.143435000 -2.380310000

===============================

**^2^C-I**

===============================

Pd -0.013682000 -0.343322000 -0.233909000

P 0.039259000 2.093716000 -0.406224000

P 2.345852000 -0.867254000 0.296951000

P -2.327473000 -0.749724000 0.444775000

C 2.457630000 -2.500136000 1.148355000

C 3.686918000 -3.142174000 1.364564000

C 1.283490000 -3.088294000 1.637971000

C 3.736921000 -4.346747000 2.064698000

H 4.603772000 -2.707831000 0.978942000

C 1.336718000 -4.290017000 2.346872000

H 0.324010000 -2.617938000 1.452770000

C 2.562712000 -4.920373000 2.560622000

H 4.692781000 -4.839085000 2.221067000

H 0.414614000 -4.728363000 2.714524000

H 2.605295000 -5.859938000 3.104738000

C 3.016256000 0.287159000 1.572946000

C 3.566860000 1.524041000 1.192118000

C 2.863343000 0.010085000 2.941166000

C 3.960372000 2.452232000 2.154671000

H 3.695147000 1.762588000 0.142805000

C 3.254530000 0.945182000 3.902173000

H 2.443595000 -0.939926000 3.256719000

C 3.805413000 2.168185000 3.513296000

H 4.378520000 3.402955000 1.837063000

H 3.134179000 0.711571000 4.956497000

H 4.109476000 2.894041000 4.261923000

C 3.703046000 -0.939517000 -0.956445000

C 5.033504000 -0.595056000 -0.665482000

C 3.385908000 -1.378433000 -2.251032000

C 6.017026000 -0.666144000 -1.653175000

H 5.306053000 -0.257555000 0.328478000

C 4.373678000 -1.457169000 -3.233386000

H 2.365141000 -1.661394000 -2.487864000

C 5.690209000 -1.094361000 -2.941177000

H 7.039572000 -0.388594000 -1.412560000

H 4.107769000 -1.801194000 -4.228945000

H 6.456969000 -1.148791000 -3.709051000

C -2.449860000 -2.242182000 1.523751000

C -2.286324000 -3.511147000 0.939456000

C -2.614150000 -2.150280000 2.914405000

C -2.300465000 -4.657788000 1.732305000

H -2.133877000 -3.598301000 -0.131325000

C -2.617074000 -3.301772000 3.704108000

H -2.749527000 -1.182854000 3.386123000

C -2.463781000 -4.558806000 3.117103000

H -2.174599000 -5.629735000 1.264014000

H -2.748362000 -3.213489000 4.779030000

H -2.472057000 -5.454202000 3.732410000

C -3.553227000 -1.055377000 -0.884444000

C -3.352589000 -0.427836000 -2.121006000

C -4.701360000 -1.837180000 -0.685734000

C -4.297258000 -0.562818000 -3.137246000

H -2.451926000 0.149351000 -2.292727000

C -5.639178000 -1.979362000 -1.709278000

H -4.858245000 -2.338555000 0.264392000

C -5.439997000 -1.338451000 -2.934669000

H -4.129161000 -0.068975000 -4.089319000

H -6.522667000 -2.591213000 -1.549967000

H -6.170120000 -1.451485000 -3.731356000

C -3.123182000 0.573111000 1.451072000

C -4.447807000 0.997210000 1.264107000

C -2.324098000 1.242301000 2.389632000

C -4.948713000 2.078862000 1.989967000

H -5.079134000 0.500899000 0.535607000

C -2.825884000 2.320261000 3.118781000

H -1.292003000 0.935044000 2.521606000

C -4.139363000 2.746567000 2.913079000

H -5.972240000 2.405502000 1.827576000

H -2.177619000 2.844269000 3.813561000

H -4.528541000 3.598032000 3.464007000

C -0.033558000 3.246603000 1.028254000

C 0.555964000 2.828063000 2.229751000

C -0.609674000 4.525384000 0.964251000

C 0.564624000 3.664231000 3.345978000

H 1.005053000 1.843794000 2.294417000

C -0.613117000 5.355683000 2.085858000

H -1.065352000 4.869747000 0.041842000

C -0.026141000 4.927751000 3.279213000

H 1.035186000 3.321721000 4.262257000

H -1.070561000 6.339140000 2.025074000

H -0.028250000 5.576841000 4.150420000

C 1.550041000 2.652765000 -1.305420000

C 2.124107000 1.771211000 -2.235654000

C 2.197285000 3.867566000 -1.030450000

C 3.326816000 2.088960000 -2.867725000

H 1.649166000 0.815220000 -2.435781000

C 3.400904000 4.182061000 -1.662150000

H 1.778259000 4.556202000 -0.305128000

C 3.971504000 3.292076000 -2.576325000

H 3.772979000 1.375252000 -3.552624000

H 3.898501000 5.120156000 -1.431919000

H 4.918338000 3.531646000 -3.051731000

C -1.355978000 2.610781000 -1.492199000

C -1.223907000 2.565856000 -2.889511000

C -2.618883000 2.886003000 -0.940052000

C -2.328494000 2.790870000 -3.711776000

H -0.260611000 2.351149000 -3.339804000

C -3.718907000 3.110658000 -1.766408000

H -2.749763000 2.922602000 0.134692000

C -3.580094000 3.061518000 -3.154388000

H -2.208284000 2.751782000 -4.790663000

H -4.687242000 3.311097000 -1.317502000

H -4.440095000 3.227486000 -3.796392000

I -0.240672000 -2.535620000 -2.186471000

===============================

**^2^M-Br**

===============================

Pd 0.053013000 -1.313894000 -0.523226000

P -1.974492000 -0.176285000 -0.151002000

C -2.614099000 0.990339000 -1.419670000

C -1.682799000 1.738780000 -2.155160000

C -3.985207000 1.182415000 -1.651765000

C -2.114625000 2.679014000 -3.089975000

H -0.621679000 1.591468000 -1.993673000

C -4.413092000 2.117462000 -2.595686000

H -4.716855000 0.600364000 -1.100287000

C -3.479757000 2.870124000 -3.312527000

H -1.379936000 3.258156000 -3.641105000

H -5.476087000 2.256187000 -2.771629000

H -3.816297000 3.597743000 -4.045642000

C -3.355752000 -1.364736000 0.109875000

C -4.392920000 -1.129289000 1.025562000

C -3.360803000 -2.548793000 -0.644290000

C -5.422961000 -2.059425000 1.174056000

H -4.391799000 -0.224335000 1.625364000

C -4.393856000 -3.473863000 -0.495715000

H -2.547444000 -2.753645000 -1.335950000

C -5.426036000 -3.230584000 0.413255000

H -6.220311000 -1.870583000 1.887322000

H -4.384643000 -4.388626000 -1.080798000

H -6.225745000 -3.955546000 0.534862000

C -1.950297000 0.814836000 1.398863000

C -2.583072000 2.059360000 1.527521000

C -1.186082000 0.320954000 2.468570000

C -2.438302000 2.801946000 2.701598000

H -3.164547000 2.461249000 0.704157000

C -1.039180000 1.065311000 3.638054000

H -0.670645000 -0.628993000 2.369085000

C -1.660577000 2.311972000 3.753672000

H -2.925014000 3.769376000 2.788971000

H -0.417113000 0.681957000 4.441038000

H -1.535329000 2.901104000 4.657620000

Br 1.374397000 -3.508317000 -0.728508000

P 1.781430000 0.252200000 -0.020898000

C 3.377029000 -0.027306000 -0.891384000

C 4.561399000 0.570888000 -0.432053000

C 3.400775000 -0.802282000 -2.057959000

C 5.751794000 0.398090000 -1.135386000

H 4.554385000 1.163796000 0.477613000

C 4.595412000 -0.967443000 -2.762796000

H 2.496776000 -1.298047000 -2.394288000

C 5.769131000 -0.369218000 -2.304055000

H 6.665717000 0.858725000 -0.771155000

H 4.606717000 -1.576545000 -3.661685000

H 6.698620000 -0.505503000 -2.849682000

C 1.426924000 2.018492000 -0.386921000

C 0.586235000 2.775861000 0.447223000

C 1.848992000 2.575589000 -1.606607000

C 0.173892000 4.051891000 0.066136000

H 0.248501000 2.372149000 1.393605000

C 1.430624000 3.852273000 -1.984323000

H 2.500564000 2.008817000 -2.263781000

C 0.588378000 4.593169000 -1.152223000

H -0.482524000 4.614358000 0.723253000

H 1.766343000 4.267652000 -2.930379000

H 0.258400000 5.583780000 -1.451038000

C 2.212305000 0.185170000 1.766248000

C 2.125612000 -1.064364000 2.404315000

C 2.613723000 1.307089000 2.506820000

C 2.417643000 -1.178666000 3.763455000

H 1.828452000 -1.941847000 1.834893000

C 2.899117000 1.187594000 3.867989000

H 2.689703000 2.277978000 2.028744000

C 2.796009000 -0.053016000 4.500259000

H 2.345624000 -2.148890000 4.246164000

H 3.201298000 2.065052000 4.432755000

H 3.014842000 -0.143688000 5.560484000

===============================

**^2^M-I**

===============================

Pd -0.120764000 1.163534000 -0.359265000

P 2.023466000 0.196641000 -0.118526000

C 2.730499000 -0.770828000 -1.512297000

C 1.840404000 -1.462429000 -2.346636000

C 4.110830000 -0.869659000 -1.748920000

C 2.321171000 -2.260124000 -3.384510000

H 0.773317000 -1.378620000 -2.178846000

C 4.588016000 -1.661737000 -2.794132000

H 4.810504000 -0.325701000 -1.121979000

C 3.695199000 -2.361578000 -3.609974000

H 1.618459000 -2.798899000 -4.012812000

H 5.657428000 -1.730089000 -2.972607000

H 4.070477000 -2.977875000 -4.422073000

C 3.299118000 1.475729000 0.234509000

C 4.386499000 1.242200000 1.090529000

C 3.172123000 2.726644000 -0.389947000

C 5.337634000 2.240777000 1.305621000

H 4.486031000 0.284517000 1.592247000

C 4.126694000 3.720501000 -0.174923000

H 2.317086000 2.927798000 -1.030495000

C 5.210747000 3.478898000 0.671949000

H 6.175078000 2.052465000 1.971527000

H 4.015829000 4.685904000 -0.659634000

H 5.949516000 4.256193000 0.845010000

C 2.132899000 -0.941425000 1.323766000

C 2.877536000 -2.129018000 1.317053000

C 1.353062000 -0.627705000 2.448662000

C 2.826155000 -2.994887000 2.411968000

H 3.473445000 -2.391207000 0.449110000

C 1.297986000 -1.495626000 3.537999000

H 0.756035000 0.278439000 2.452471000

C 2.030644000 -2.685791000 3.517973000

H 3.399608000 -3.917559000 2.394920000

H 0.661723000 -1.252665000 4.383478000

H 1.978241000 -3.371611000 4.358679000

I -1.622875000 3.415461000 -0.347304000

P -1.672257000 -0.608067000 -0.007304000

C -3.303830000 -0.391076000 -0.827072000

C -4.445460000 -1.068662000 -0.371869000

C -3.392149000 0.426835000 -1.961290000

C -5.657266000 -0.930180000 -1.046764000

H -4.390522000 -1.695153000 0.513096000

C -4.605894000 0.555709000 -2.639309000

H -2.520483000 0.979908000 -2.294953000

C -5.738046000 -0.120419000 -2.183169000

H -6.538851000 -1.451241000 -0.684307000

H -4.666382000 1.197697000 -3.512960000

H -6.684240000 -0.011291000 -2.705588000

C -1.150796000 -2.278130000 -0.574776000

C -0.215824000 -3.023911000 0.163552000

C -1.547781000 -2.749761000 -1.837500000

C 0.312297000 -4.206279000 -0.351992000

H 0.103098000 -2.683115000 1.140730000

C -1.013725000 -3.933133000 -2.349951000

H -2.270353000 -2.189993000 -2.422421000

C -0.079321000 -4.662957000 -1.611942000

H 1.038771000 -4.761668000 0.233555000

H -1.331910000 -4.285011000 -3.327310000

H 0.339372000 -5.580193000 -2.015516000

C -2.059587000 -0.793330000 1.781023000

C -2.046436000 0.368574000 2.571601000

C -2.355626000 -2.026426000 2.381946000

C -2.309913000 0.291181000 3.939131000

H -1.823201000 1.329431000 2.114799000

C -2.612208000 -2.099316000 3.752113000

H -2.370426000 -2.933417000 1.786771000

C -2.585476000 -0.942850000 4.534123000

H -2.294795000 1.196524000 4.538830000

H -2.832188000 -3.061017000 4.206919000

H -2.782376000 -1.002033000 5.600835000

===============================

**^2^M-Br-TS**

===============================

Pd 0.196867000 0.077391000 -1.337707000

P -1.650444000 0.907750000 0.012553000

C -1.655067000 2.697842000 0.394823000

C -0.426958000 3.322137000 0.662720000

C -2.842910000 3.441022000 0.476866000

C -0.392425000 4.666259000 1.027896000

H 0.498382000 2.768132000 0.572105000

C -2.799298000 4.790253000 0.831537000

H -3.797150000 2.968992000 0.266953000

C -1.575963000 5.403195000 1.111218000

H 0.564827000 5.134143000 1.235430000

H -3.721945000 5.360763000 0.888676000

H -1.545454000 6.453739000 1.386346000

C -3.335135000 0.539843000 -0.605009000

C -4.372020000 0.131591000 0.247214000

C -3.579937000 0.666190000 -1.980652000

C -5.639654000 -0.140915000 -0.272278000

H -4.189449000 0.017542000 1.310942000

C -4.848535000 0.402073000 -2.492622000

H -2.769809000 0.944175000 -2.647990000

C -5.878925000 -0.005064000 -1.640464000

H -6.435618000 -0.463455000 0.392903000

H -5.027627000 0.496033000 -3.559385000

H -6.863523000 -0.223085000 -2.044144000

C -1.595357000 0.104455000 1.665807000

C -1.804060000 0.812549000 2.858465000

C -1.270676000 -1.259751000 1.725104000

C -1.677262000 0.164266000 4.088797000

H -2.043412000 1.870173000 2.826514000

C -1.131722000 -1.901425000 2.955163000

H -1.099596000 -1.808094000 0.806317000

C -1.332208000 -1.188753000 4.140723000

H -1.836953000 0.721047000 5.007913000

H -0.838667000 -2.946425000 2.981578000

H -1.214017000 -1.683813000 5.100254000

Br -1.189487000 -2.009017000 -2.210296000

P 2.090315000 -0.432215000 0.112460000

C 3.715861000 -0.571438000 -0.733131000

C 4.892342000 -0.757431000 0.011248000

C 3.781812000 -0.523550000 -2.131011000

C 6.117869000 -0.890832000 -0.637102000

H 4.848926000 -0.796007000 1.095691000

C 5.012643000 -0.662572000 -2.777034000

H 2.881223000 -0.353450000 -2.710802000

C 6.178422000 -0.844910000 -2.033611000

H 7.024579000 -1.030798000 -0.055502000

H 5.055717000 -0.619204000 -3.861127000

H 7.134721000 -0.949188000 -2.538335000

C 2.319012000 0.898527000 1.348157000

C 1.588107000 0.906108000 2.547837000

C 3.087276000 2.024469000 1.006417000

C 1.642387000 2.010353000 3.397746000

H 0.968364000 0.059396000 2.817959000

C 3.136321000 3.125337000 1.861543000

H 3.620739000 2.049964000 0.062903000

C 2.416322000 3.121709000 3.059021000

H 1.065657000 2.001045000 4.317722000

H 3.731759000 3.990315000 1.584569000

H 2.451533000 3.983599000 3.719045000

C 1.981370000 -2.005446000 1.062289000

C 1.350995000 -3.095427000 0.441562000

C 2.527026000 -2.171403000 2.344778000

C 1.258755000 -4.323492000 1.098047000

H 0.913129000 -2.977637000 -0.545231000

C 2.422323000 -3.397719000 3.002148000

H 3.019387000 -1.341413000 2.840225000

C 1.786003000 -4.475685000 2.382832000

H 0.770149000 -5.158945000 0.605006000

H 2.841007000 -3.510434000 3.998189000

H 1.706120000 -5.429553000 2.896370000

Br 1.362375000 2.231583000 -2.292524000

C -2.271351000 -4.094075000 -0.485082000

C -3.583674000 -3.457545000 -0.192642000

H -1.457162000 -4.010051000 0.226801000

H -2.136672000 -4.788395000 -1.305052000

C -4.091892000 -3.738456000 1.235987000

H -3.488160000 -2.368906000 -0.310004000

H -4.329058000 -3.771054000 -0.931425000

H -3.371422000 -3.383749000 1.980079000

H -5.037614000 -3.214264000 1.411407000

H -4.256843000 -4.807716000 1.403303000

===============================

**^2^M-I-TS**

===============================

C -3.069061000 -4.235836000 0.867846000

C -4.243353000 -3.318180000 0.955026000

H -2.048841000 -3.878301000 0.948751000

H -3.197296000 -5.209373000 0.401305000

C -4.852939000 -2.983888000 -0.426405000

H -5.028117000 -3.773120000 1.580168000

H -3.957011000 -2.384655000 1.451519000

H -5.046077000 -3.897847000 -0.998469000

H -5.799349000 -2.445223000 -0.312426000

H -4.172180000 -2.358778000 -1.010216000

Pd 0.174098000 0.070894000 -1.128765000

P -1.477571000 1.142428000 0.269018000

I -1.093840000 -2.140193000 -2.187160000

P 1.898380000 -0.710727000 0.385048000

I 1.532957000 2.202884000 -2.237639000

C -1.291796000 2.929108000 0.619456000

C -3.162921000 0.961036000 -0.420352000

C -1.552721000 0.360111000 1.923337000

C -0.022159000 3.407158000 0.975558000

C -2.381996000 3.811642000 0.592869000

C 0.150619000 4.745780000 1.318664000

H 0.829026000 2.740059000 0.974518000

C -2.199598000 5.154995000 0.925991000

H -3.367693000 3.455389000 0.313500000

C -0.936227000 5.622964000 1.292457000

H 1.139143000 5.099498000 1.594620000

H -3.046645000 5.834298000 0.897852000

H -0.798272000 6.669231000 1.549987000

C -4.284983000 0.830439000 0.412344000

C -3.331811000 1.004251000 -1.812158000

C -5.562561000 0.765028000 -0.144157000

H -4.161600000 0.782891000 1.489780000

C -4.611152000 0.933086000 -2.362031000

H -2.465437000 1.073983000 -2.462638000

C -5.726816000 0.816734000 -1.530036000

H -6.427366000 0.663365000 0.504963000

H -4.733158000 0.958488000 -3.440460000

H -6.721816000 0.755403000 -1.961081000

C -1.649379000 1.100260000 3.111039000

C -1.456878000 -1.037954000 1.985602000

C -1.634409000 0.443020000 4.342492000

H -1.714202000 2.182343000 3.073305000

C -1.439131000 -1.691269000 3.216735000

H -1.374983000 -1.603603000 1.064884000

C -1.521657000 -0.949378000 4.397983000

H -1.704349000 1.020340000 5.260045000

H -1.346584000 -2.772245000 3.245515000

H -1.496849000 -1.454382000 5.359225000

C 3.430888000 -1.064044000 -0.560543000

C 2.342065000 0.619544000 1.563432000

C 1.672089000 -2.223592000 1.410478000

C 4.677696000 -1.099288000 0.084443000

C 3.347353000 -1.361873000 -1.926843000

C 5.827695000 -1.418344000 -0.634846000

H 4.749830000 -0.862713000 1.141829000

C 4.501919000 -1.687324000 -2.640552000

H 2.388898000 -1.332070000 -2.435416000

C 5.740370000 -1.713261000 -1.998376000

H 6.790708000 -1.434867000 -0.132730000

H 4.429847000 -1.911307000 -3.700407000

H 6.637848000 -1.960126000 -2.558196000

C 1.676993000 0.735034000 2.796110000

C 3.233957000 1.631688000 1.168772000

C 1.916292000 1.831017000 3.624522000

H 0.971004000 -0.023714000 3.112297000

C 3.471330000 2.722561000 2.004649000

H 3.723219000 1.578041000 0.203038000

C 2.814125000 2.825784000 3.233667000

H 1.387800000 1.907219000 4.569815000

H 4.161927000 3.497823000 1.686731000

H 2.995076000 3.680955000 3.878307000

C 0.876322000 -3.267360000 0.917293000

C 2.320816000 -2.383209000 2.646112000

C 0.709824000 -4.437811000 1.658504000

H 0.372031000 -3.162505000 -0.037490000

C 2.145516000 -3.552088000 3.386547000

H 2.954856000 -1.595843000 3.038319000

C 1.335040000 -4.579660000 2.898804000

H 0.083645000 -5.232797000 1.265012000

H 2.645856000 -3.659270000 4.344738000

H 1.197095000 -5.487147000 3.479497000

===============================

**^1^K-Br**

===============================

Pd 0.000030000 0.000010000 -0.000104000

P 2.390886000 0.057123000 0.018223000

C 3.077278000 0.359794000 1.695959000

C 2.279128000 0.951906000 2.685887000

C 4.404522000 0.010353000 2.002600000

C 2.804166000 1.197937000 3.955467000

H 1.257735000 1.234460000 2.459185000

C 4.924336000 0.262697000 3.271587000

H 5.026788000 -0.468852000 1.253804000

C 4.124726000 0.856896000 4.250745000

H 2.176314000 1.657839000 4.712933000

H 5.951581000 -0.010000000 3.495696000

H 4.529378000 1.048939000 5.240420000

C 3.290651000 -1.445743000 -0.534515000

C 3.954611000 -1.497350000 -1.767403000

C 3.291624000 -2.581033000 0.293099000

C 4.614335000 -2.663368000 -2.162093000

H 3.966985000 -0.630590000 -2.418869000

C 3.959916000 -3.737407000 -0.100221000

H 2.766308000 -2.560466000 1.241501000

C 4.621408000 -3.782687000 -1.330187000

H 5.127620000 -2.690088000 -3.119023000

H 3.953303000 -4.607664000 0.549261000

H 5.137493000 -4.687603000 -1.638022000

C 3.052543000 1.372723000 -1.075928000

C 4.219756000 2.084525000 -0.774939000

C 2.380694000 1.624657000 -2.281443000

C 4.710131000 3.034502000 -1.672669000

H 4.740599000 1.910799000 0.160641000

C 2.880971000 2.563992000 -3.181052000

H 1.457666000 1.097634000 -2.503330000

C 4.045825000 3.272690000 -2.876646000

H 5.610609000 3.590234000 -1.427416000

H 2.352574000 2.753780000 -4.110648000

H 4.429365000 4.013469000 -3.572379000

Br -0.043163000 -2.549127000 -0.277608000

P -2.390893000 -0.057162000 -0.018252000

C -3.052387000 -1.373026000 1.075708000

C -4.219608000 -2.084799000 0.774668000

C -2.380491000 -1.625114000 2.281157000

C -4.709938000 -3.034913000 1.672271000

H -4.740474000 -1.910938000 -0.160872000

C -2.880729000 -2.564590000 3.180649000

H -1.457445000 -1.098145000 2.503104000

C -4.045586000 -3.273258000 2.876195000

H -5.610401000 -3.590650000 1.426973000

H -2.352285000 -2.754500000 4.110195000

H -4.429098000 -4.014122000 3.571852000

C -3.290605000 1.445576000 0.535058000

C -3.291837000 2.581012000 -0.292350000

C -3.954289000 1.496867000 1.768102000

C -3.960132000 3.737266000 0.101349000

H -2.766730000 2.560646000 -1.240864000

C -4.614027000 2.662758000 2.163160000

H -3.966434000 0.629976000 2.419398000

C -4.621364000 3.782234000 1.331465000

H -3.953760000 4.607662000 -0.547945000

H -5.127121000 2.689269000 3.120197000

H -5.137463000 4.687050000 1.639584000

C -3.077415000 -0.359415000 -1.695956000

C -2.279320000 -0.951337000 -2.686025000

C -4.404656000 -0.009816000 -2.002480000

C -2.804395000 -1.197038000 -3.955651000

H -1.257947000 -1.234008000 -2.459359000

C -4.924495000 -0.261820000 -3.271516000

H -5.026870000 0.469268000 -1.253564000

C -4.124933000 -0.855837000 -4.250831000

H -2.176582000 -1.656801000 -4.713237000

H -5.951726000 0.011003000 -3.495541000

H -4.529600000 -1.047618000 -5.240547000

Br 0.043044000 2.549160000 0.277115000

===============================

**^1^K-I**

===============================

Pd 0.001963000 -0.003082000 -0.142956000

P 2.379848000 -0.056515000 -0.049890000

C 3.264569000 -1.276817000 -1.099814000

C 2.654011000 -1.767829000 -2.260360000

C 4.598404000 -1.615978000 -0.825323000

C 3.364219000 -2.597004000 -3.129254000

H 1.619383000 -1.520050000 -2.471263000

C 5.299751000 -2.457385000 -1.687693000

H 5.088093000 -1.224739000 0.061104000

C 4.683886000 -2.948170000 -2.841742000

H 2.879765000 -2.977254000 -4.023657000

H 6.327925000 -2.724588000 -1.461262000

H 5.232527000 -3.601190000 -3.514411000

C 3.331613000 1.455634000 -0.486711000

C 4.479062000 1.842979000 0.217025000

C 2.958639000 2.162746000 -1.638421000

C 5.236598000 2.930580000 -0.222257000

H 4.780762000 1.305989000 1.110019000

C 3.722915000 3.240370000 -2.079842000

H 2.057086000 1.882892000 -2.173975000

C 4.861966000 3.629173000 -1.370278000

H 6.119900000 3.229338000 0.335000000

H 3.420168000 3.785923000 -2.968648000

H 5.452007000 4.475700000 -1.709540000

C 2.791483000 -0.422505000 1.693282000

C 3.117514000 -1.718037000 2.117584000

C 2.634943000 0.600791000 2.643740000

C 3.312806000 -1.978518000 3.474562000

H 3.201362000 -2.521247000 1.394546000

C 2.833127000 0.332691000 3.997176000

H 2.349837000 1.597973000 2.322321000

C 3.174887000 -0.956275000 4.414961000

H 3.565528000 -2.984873000 3.795217000

H 2.715695000 1.130554000 4.724755000

H 3.326211000 -1.163988000 5.470410000

I -0.000775000 2.699506000 0.348254000

P -2.396042000 0.049633000 -0.125086000

C -3.037245000 1.439469000 -1.144207000

C -4.159973000 2.195296000 -0.789328000

C -2.375031000 1.716349000 -2.350518000

C -4.617584000 3.208100000 -1.634593000

H -4.669566000 2.013334000 0.150092000

C -2.843695000 2.716923000 -3.199104000

H -1.477906000 1.161611000 -2.610581000

C -3.966428000 3.467096000 -2.840979000

H -5.482670000 3.797519000 -1.344682000

H -2.322049000 2.922801000 -4.129129000

H -4.325206000 4.256442000 -3.495145000

C -3.320688000 -1.395174000 -0.795531000

C -3.563996000 -2.508481000 0.025896000

C -3.745595000 -1.428608000 -2.131277000

C -4.233397000 -3.622146000 -0.476842000

H -3.228993000 -2.505383000 1.056743000

C -4.406614000 -2.550857000 -2.632909000

H -3.576288000 -0.578058000 -2.782158000

C -4.654616000 -3.648200000 -1.807841000

H -4.416133000 -4.474083000 0.171326000

H -4.733843000 -2.560584000 -3.668617000

H -5.172387000 -4.519232000 -2.199151000

C -3.071743000 0.233947000 1.569652000

C -2.211925000 0.542410000 2.633066000

C -4.442169000 0.048343000 1.825772000

C -2.717547000 0.681190000 3.926494000

H -1.152116000 0.677372000 2.449889000

C -4.943139000 0.195255000 3.118232000

H -5.112681000 -0.228674000 1.018287000

C -4.081007000 0.513884000 4.170647000

H -2.040924000 0.918275000 4.742001000

H -6.003742000 0.052876000 3.304021000

H -4.471415000 0.622568000 5.178512000

I 0.012743000 -2.729122000 0.032293000

===============================

**^1^C-anion**

===============================

Pd -0.053258000 0.102609000 -0.574228000

P 1.881966000 1.335471000 0.030415000

P 0.208334000 -2.162531000 -0.007899000

P -2.153330000 0.917911000 0.004574000

C -1.373175000 -3.140249000 0.048920000

C -1.676016000 -4.153113000 0.970105000

C -2.331487000 -2.803590000 -0.919510000

C -2.925118000 -4.778231000 0.951042000

H -0.945427000 -4.438615000 1.720955000

C -3.574122000 -3.436892000 -0.951153000

H -2.106506000 -2.012406000 -1.626764000

C -3.879776000 -4.418194000 -0.004356000

H -3.153486000 -5.547356000 1.685394000

H -4.306771000 -3.135988000 -1.695149000

H -4.854732000 -4.899376000 -0.009314000

C 0.886709000 -2.298288000 1.701462000

C 2.278581000 -2.287019000 1.907859000

C 0.047595000 -2.151452000 2.821597000

C 2.808358000 -2.156497000 3.191343000

H 2.954405000 -2.356688000 1.062011000

C 0.581591000 -2.030553000 4.104179000

H -1.027762000 -2.104334000 2.694097000

C 1.964982000 -2.034594000 4.297801000

H 3.887471000 -2.132528000 3.317456000

H -0.093634000 -1.911071000 4.947725000

H 2.380950000 -1.922557000 5.295633000

C 1.253468000 -3.350979000 -0.980336000

C 1.874947000 -4.486390000 -0.438511000

C 1.396552000 -3.083518000 -2.350474000

C 2.643298000 -5.326738000 -1.244847000

H 1.771724000 -4.704557000 0.620407000

C 2.153544000 -3.935415000 -3.158277000

H 0.938810000 -2.191959000 -2.777102000

C 2.784539000 -5.053426000 -2.608513000

H 3.129706000 -6.196780000 -0.809628000

H 2.258913000 -3.710516000 -4.216479000

H 3.384387000 -5.708394000 -3.236138000

C -3.627151000 0.502790000 -1.051183000

C -3.364929000 0.200457000 -2.396665000

C -4.953665000 0.477717000 -0.593734000

C -4.410940000 -0.135753000 -3.259631000

H -2.337534000 0.218532000 -2.760765000

C -5.995351000 0.129950000 -1.454775000

H -5.172899000 0.709034000 0.444543000

C -5.725896000 -0.180627000 -2.790840000

H -4.189764000 -0.370704000 -4.297440000

H -7.017157000 0.101166000 -1.083241000

H -6.537593000 -0.454114000 -3.460918000

C -2.363390000 2.760734000 0.124340000

C -1.471285000 3.544338000 -0.620633000

C -3.366075000 3.397281000 0.872525000

C -1.580613000 4.937186000 -0.620397000

H -0.689611000 3.052405000 -1.193936000

C -3.467263000 4.788512000 0.883184000

H -4.060295000 2.802779000 1.459488000

C -2.574944000 5.561934000 0.133911000

H -0.873968000 5.524121000 -1.199879000

H -4.242608000 5.270122000 1.474672000

H -2.654624000 6.646420000 0.143121000

C -2.677543000 0.347124000 1.685602000

C -2.208968000 1.018028000 2.829963000

C -3.361392000 -0.869106000 1.866843000

C -2.418905000 0.495825000 4.105903000

H -1.661276000 1.948449000 2.720025000

C -3.574764000 -1.388448000 3.144744000

H -3.714175000 -1.426130000 1.006723000

C -3.104577000 -0.710750000 4.272452000

H -2.040304000 1.033784000 4.971573000

H -4.095524000 -2.336543000 3.250135000

H -3.264821000 -1.119502000 5.266811000

C 2.026204000 1.886679000 1.795003000

C 1.207460000 1.231034000 2.724433000

C 2.913552000 2.872635000 2.260559000

C 1.270384000 1.545624000 4.082777000

H 0.520517000 0.468594000 2.373998000

C 2.969429000 3.196946000 3.616954000

H 3.556341000 3.392994000 1.556945000

C 2.147845000 2.532819000 4.532921000

H 0.630270000 1.011268000 4.778569000

H 3.656812000 3.966826000 3.959656000

H 2.193403000 2.786317000 5.589385000

C 3.407392000 0.285941000 -0.156578000

C 3.435470000 -0.587367000 -1.258900000

C 4.438303000 0.204644000 0.791352000

C 4.464184000 -1.518190000 -1.404215000

H 2.622587000 -0.556716000 -1.980790000

C 5.463634000 -0.733905000 0.648349000

H 4.429550000 0.849691000 1.663162000

C 5.478097000 -1.601159000 -0.446064000

H 4.443851000 -2.204786000 -2.245437000

H 6.244594000 -0.794464000 1.403138000

H 6.265709000 -2.344122000 -0.545280000

C 2.309128000 2.865330000 -0.915412000

C 2.926352000 2.768101000 -2.173819000

C 1.852582000 4.127008000 -0.494243000

C 3.112448000 3.901021000 -2.965076000

H 3.243579000 1.801715000 -2.547551000

C 2.044117000 5.259907000 -1.286116000

H 1.333900000 4.226702000 0.453552000

C 2.679348000 5.153597000 -2.525171000

H 3.587492000 3.798932000 -3.937222000

H 1.690244000 6.225581000 -0.932316000

H 2.824102000 6.034379000 -3.145631000

Br 0.331714000 0.359180000 -3.483721000

===============================

**^2^K-Br-anion**

===============================

Pd -0.000101000 0.000275000 0.990344000

P 2.161453000 0.064472000 -0.017289000

C 2.494840000 -1.405787000 -1.087034000

C 1.397068000 -2.118822000 -1.590205000

C 3.790148000 -1.808685000 -1.444424000

C 1.588272000 -3.200463000 -2.450404000

H 0.393745000 -1.840172000 -1.290666000

C 3.981358000 -2.896854000 -2.297757000

H 4.649679000 -1.273133000 -1.052000000

C 2.880509000 -3.591991000 -2.805619000

H 0.721385000 -3.738371000 -2.823618000

H 4.990270000 -3.202820000 -2.563923000

H 3.031780000 -4.440929000 -3.468098000

C 2.368690000 1.465511000 -1.203804000

C 2.192655000 2.771209000 -0.709474000

C 2.574939000 1.285937000 -2.580619000

C 2.233452000 3.864801000 -1.573171000

H 1.970902000 2.923770000 0.342338000

C 2.606711000 2.384557000 -3.443051000

H 2.703879000 0.286880000 -2.983154000

C 2.438801000 3.677157000 -2.943552000

H 2.082845000 4.863888000 -1.173381000

H 2.761026000 2.226222000 -4.507609000

H 2.460296000 4.530975000 -3.616381000

C 3.676409000 0.156970000 1.029033000

C 4.810258000 0.916073000 0.705014000

C 3.663552000 -0.587048000 2.221032000

C 5.918306000 0.927791000 1.555591000

H 4.823139000 1.503869000 -0.207944000

C 4.777030000 -0.580012000 3.060745000

H 2.779464000 -1.166298000 2.479513000

C 5.905147000 0.177374000 2.732722000

H 6.790152000 1.524287000 1.297718000

H 4.757757000 -1.159992000 3.979624000

H 6.767088000 0.188197000 3.395394000

Br -0.280962000 2.571000000 1.903548000

P -2.161611000 -0.064329000 -0.017186000

C -2.495050000 1.405734000 -1.087151000

C -3.790360000 1.807901000 -1.445406000

C -1.397355000 2.119243000 -1.589755000

C -3.981642000 2.895773000 -2.299085000

H -4.649846000 1.271935000 -1.053434000

C -1.588614000 3.200606000 -2.450305000

H -0.394081000 1.841169000 -1.289544000

C -2.880821000 3.591373000 -2.806419000

H -4.990546000 3.201136000 -2.565965000

H -0.721785000 3.738885000 -2.823129000

H -3.032115000 4.440092000 -3.469173000

C -2.368815000 -1.465508000 -1.203463000

C -2.193181000 -2.771113000 -0.708718000

C -2.574477000 -1.286223000 -2.580406000

C -2.233828000 -3.864912000 -1.572144000

H -1.971849000 -2.923399000 0.343225000

C -2.606102000 -2.385069000 -3.442558000

H -2.703039000 -0.287234000 -2.983239000

C -2.438619000 -3.677566000 -2.942655000

H -2.083547000 -4.863929000 -1.172058000

H -2.759933000 -2.226999000 -4.507223000

H -2.460011000 -4.531557000 -3.615269000

C -3.676534000 -0.156713000 1.029205000

C -3.663559000 0.587227000 2.221256000

C -4.810495000 -0.915653000 0.705201000

C -4.777003000 0.580286000 3.061014000

H -2.779381000 1.166335000 2.479731000

C -5.918522000 -0.927277000 1.555807000

H -4.823533000 -1.503380000 -0.207797000

C -5.905229000 -0.176935000 2.732982000

H -4.757631000 1.160204000 3.979929000

H -6.790429000 -1.523676000 1.297922000

H -6.767143000 -0.187668000 3.395693000

Br 0.281557000 -2.571185000 1.903002000

===============================

**E-cat**

===============================

C 1.886182000 1.238092000 -0.354477000

C 0.824893000 1.245299000 0.515432000

C 0.210311000 -0.000039000 1.001839000

C 0.824917000 -1.245336000 0.515349000

C 1.886210000 -1.238053000 -0.354555000

C 2.404476000 0.000039000 -0.790423000

H 2.327157000 2.163069000 -0.709475000

H 0.404562000 2.183902000 0.867736000

H 2.327204000 -2.162998000 -0.709613000

H 3.240888000 0.000070000 -1.485265000

C -1.373121000 -0.000063000 0.857968000

H -1.746552000 -0.880627000 1.390420000

H -1.746587000 0.880415000 1.390538000

C -1.848090000 0.000035000 -0.599025000

H -1.444794000 0.879929000 -1.115952000

H -1.444780000 -0.879780000 -1.116076000

C -3.376630000 0.000025000 -0.684495000

H -3.801652000 0.885649000 -0.201255000

H -3.703536000 0.000102000 -1.727690000

H -3.801632000 -0.885681000 -0.201389000

H 0.350231000 -0.000076000 2.104767000

H 0.404592000 -2.183969000 0.867581000
